# Genome-wide quantification of polycistronic transcription in *Leishmania major*

**DOI:** 10.1128/mbio.02241-24

**Published:** 2024-11-25

**Authors:** Janne Grünebast, Stephan Lorenzen, Joachim Clos

**Affiliations:** 1 Leishmania Genetics Group, Bernhard Nocht Institute for Tropical Medicine, Hamburg, Germany; 2 Department of Infection Epidemiology, Bernhard Nocht Institute for Tropical Medicine, Hamburg, Germany; The George Washington University Milken Institute of Public Health, Washington, DC, USA

**Keywords:** nuclear run-on, strand switch regions, PRO-seq, RNA polymerase pausing, transcription initiation

## Abstract

*Leishmania major* is a human-pathogenic, obligate parasite and the etiological agent of the most prevalent, cutaneous form of leishmaniasis, which is an important neglected, tropical disease with ~1.2 million new infections per year. *Leishmania*, and the whole order Trypanosomatida, are early eukaryotes with highly diverged gene expression and regulation pathways, setting them apart from their mammalian hosts and from most other eukaryotes. Using precision run-on sequence analysis, we performed a genome-wide mapping and density analysis of RNA polymerases in isolated nuclei of the protozoan parasite *Leishmania major*. We map transcription initiation sites at divergent strand switch regions and head-tail regions within the chromosomes and correlate them with known sites of chromatin modifications. We confirm continuous, polycistronic RNA synthesis in all RNA polymerase II-dependent gene arrays but find small varying RNA polymerase activities in polycistronic transcription units (PTUs), excluding gene-specific transcription regulation, but not PTU-specific variations. Lastly, we find evidence for transcriptional pausing of all three RNA polymerase classes, hinting at a possible mechanism of transcriptional regulation.

**IMPORTANCE:**

*Leishmania* spp. are pathogens of humans and animals and cause one of the most important neglected tropical diseases. Regulation of gene expression in *Leishmania* but also in the related *Trypanosoma* is radically different from all eukaryotic model organisms, dispensing with regulated, gene-specific transcription, and relying instead on highly regulated translation. Our work sheds light on the initiation, elongation, and termination of transcription, maps unidirectional, polycistronic transcription units, provides evidence for transcriptional pausing at or near starting points of RNA synthesis, and quantifies the varying transcription rates of the polycistronic transcription units. Our results will further the understanding of these important pathogens and should provide a valuable resource for researchers in the field of eukaryotic microbiology.

## INTRODUCTION

The eukaryotic genus *Leishmania* comprises a large number of parasitic protozoa which cause morbidity and mortality in humans and in a range of domestic and wild animals. The three main clinical forms of human *Leishmania* infections are cutaneous, mucocutaneous, and visceral leishmaniasis. The latter form is lethal if left untreated. With 1.6 million new infections per year and an estimated 20,000 fatalities (1), leishmaniasis is ranked second among the neglected tropical diseases in terms of human suffering and mortality (2). There are no vaccines for human use, and the available chemotherapeutics are expensive and fraught with severe side effects (3).

The order Trypanosomatida, to which the sub-family Leishmaniiae belongs, is an early branch of the eukaryotic tree, set apart from the crown group of Eukaryota by their peculiar gene regulation mechanisms. All protein-coding genes are arranged in so-called polycistronic transcription units (PTUs), large unidirectional arrays of functionally non-related genes (4, 5), separated by converging or diverging strand switch regions (cSSRs or dSSRs, respectively) which, according to the current view, are the places of transcription termination or initiation, respectively. Protein-coding genes of *Leishmania* have no distinct promoters, and the genomes harbor no genes for canonical transcription factors (6, 7). However, recent studies in *Trypanosoma brucei* suggest the presence of sequence-specific motifs that act as core promoters in unidirectional PTUs (8, 9). As the name implies, polycistronic transcription units are thought to be transcribed in a continuous manner. The resulting pre-mRNAs are processed by polyadenylation and trans-splicing into monocistronic mRNAs, which are translocated to the cytoplasm for translation (7). Each *Leishmania* mRNA therefore carries the same 39-nt spliced leader RNA sequence at the 5′ end which also bears a unique cap structure.

Polycistronic transcription was first investigated for loci and gene arrays by nuclear run-on transcription analysis using radioactive labeling of nascent RNA and hybridization to immobilized gene sequences, followed by autoradiography (10–13). The results supported the concept of a polycistronic transcription and ruled out an inducible transcription of the investigated genes, mostly heat shock genes. The same methodology was also applied to extended gene arrays (14) and to small *Leishmania* chromosomes (15–17), also supporting the concept of polycistronic transcription.

Epigenetic factors affecting transcription were analyzed, too. Increased appearance of a modified thymine base, BaseJ, maps to presumed regions of transcription termination, i.e., cSSRs (18), while acetylated histone 3 (H3ac) is found predominantly in nucleosomes near transcription start regions, i.e., dSSRs (19). The combination of BaseJ followed by a region of H3ac also marks potential internal transcription termination and initiation sites within PTUs, so-called head-tail regions (18, 20, 21). Moreover, chromatin density was found to fluctuate at dSSRs and 5′-telomeric sites, regions of transcription initiation, during *in vitro* stage conversion of *Leishmania donovani* (22).

We therefore decided to investigate RNA synthesis in *Leishmania* on a genome-wide scale, applying Precision Run-On sequencing (PRO-seq) (23). By performing a nuclear run-on reaction in the presence of biotinylated CTP, we at once labeled and terminated nascent RNAs in isolated nuclei, thereby mapping the positions of RNA polymerase elongation complexes. We show that transcription is truly polycistronic and unidirectional in all PTUs in the *L. major* genome, but find that PTUs are transcribed at different rates suggesting either an underlying mechanism of transcriptional control or varying efficacies of transcription initiation sites. We verify dSSRs and cSSRs as sites of transcription initiation and termination, respectively, and establish a correlation between chromatin modifications and transcription initiation/termination, but also find that BaseJ and H3ac do not always cause termination and reinitiation of transcription within PTUs. Furthermore, we detect a higher abundance of paused RNA polymerase complexes within transcription initiation sites in dSSRs and 5′-telomeric transcription start sites, but also in RNA polymerase I- and III-dependent genes, suggesting pausing of RNA polymerases as a new mechanism of RNA synthesis modulation.

## MATERIALS AND METHODS

### Cell culture of *L. major*

*Leishmania major* strain Friedlin was cultured at 25°C in M199+ medium (1× M199, 20% FCS [heat-inactivated], 40 mM HEPES, 10 mg/L hemin, 0.1 mM adenine, 5 µM 6-biopterin, 2 mM L-glutamine, 100 U penicillin, 100 µg/mL streptomycin). The parasites were diluted every 3–4 days to a cell count of 1 × 10^5^.

### Nuclei isolation from *L. major*

From a logarithmically growing culture (2 × 10^7^ cells/mL), 2 × 10^9^ cells were taken and sedimented at 4°C. The pellet was then washed with ice-cold 1× PBS (4°C, 15 min, 1,000 × *g*), and the cells were lysed in 5 mL ice-cold lysis buffer (10 mM Tris-HCl [pH 8.0], 10 mM sodium chloride, 5 mM DTT, 1.5 mM magnesium chloride, 1 mM spermidine, 1 mM EDTA, 0.1 mM PMSF) containing 0.5% (vol/vol) Tergitol 15-S-9 (Sigma-Aldrich) for 5 min on ice. After centrifugation (4°C, 15 min, 1,000 × *g*), the cytosolic supernatant was carefully decanted, and the nuclei were washed in 5 mL of ice-cold lysis buffer without Tergitol. The nuclei were then resuspended in 200 µL of lysis buffer without Tergitol, and an equal volume of 2× nuclei storage buffer (80% [vol/vol] glycerin, 10 mM Tris-HCl [pH 8.3], 0.2 mM EDTA) was added.

### Nuclear run-on and RNA isolation

The nuclear run-on protocol follows a protocol by Mahat et al. (23) with slight adaptions to *Leishmania* gene expression mechanisms. Individual run-ons with biotinylated CTP were performed. First, the 2× run-on mix (2× NRO) was prepared as follows: 40 µL 5× nuclear run-on buffer (80 mM Tris-HCL [pH 7.5], 50 mM sodium chloride, 50 mM potassium chloride, 8 mM magnesium chloride, 0.5 mM spermidine [Sigma-Aldrich]), 2.5 µL ATP (10 mM), 2.5 µL GTP (10 mM), 2.5 µL UTP (10 mM), 1 µL CTP (0.05 mM), 5 µL biotin-11-CTP (1 mM) (Jena Bioscience), 0.8 µL DTT (0.5 M) 40 U SUPERase-InTM RNase Inhibitor (Ambion) and 43.7 µL 2% Sarkosyl (Sigma-Aldrich) for run-ons with sarkosyl, or nuclease-free water for run-ons without sarkosyl. For the nuclear run-on reaction, 100 µL of nuclei suspension was added to 100 µL of prewarmed 2× NRO using a wide bore pipette tip. The reaction was incubated for 10 min at 37°C and stopped by the addition of 500 µL Trizol LS (Invitrogen), followed by vigorous mixing. A volume of 130 µL chloroform was added, and the sample was shaken and then centrifuged at 14,000 × *g* for 5 min at 4°C. The aqueous phase was transferred to a new tube and mixed with 2.5 volumes 100% (vol/vol) ethanol. After incubation for 10 min at room temperature, the RNA was sedimented at 14,000 × *g* for 20 min at 4°C. The supernatant was completely removed, and the pellet was washed once with 75% (vol/vol) ethanol. The pellet was dried and dissolved in 20 uL of nuclease-free water.

### PRO-seq library preparation

The library was prepared as in Mahat et al. (23), but without hydroxyl repair of the 5′ end due to the different cap structure in trypanosomatidic spliced leader RNA. Briefly, RNA fragmentation was performed by base hydrolysis with 5 µL 1 N NaOH. The sample was neutralized by 25 µL 1 M Tris-HCl (pH 6.8), and a buffer exchange was performed using a BioSpin P-30 column (Bio-Rad) according to the manufacturer’s instructions.

Biotin-labeled RNAs were enriched using streptavidin M280 beads (Sigma-Aldrich). For each library, 90 µL beads were washed in preparation buffer (0.1 N NaOH, 50 mM NaCl) and then twice in 100 nM NaCl. The beads were then resuspended in 50 µL binding buffer (10 mM Tris-HCl [pH 7.4], 300 mM NaCl, 0.1% [vol/vol] Tergitol 15-S-9) per reaction and incubated with 50 µL of nuclear run-on RNA for 20 min at room temperature on a rotating wheel (8 rpm). Beads were washed twice with high salt buffer (50 mM Tris-HCl [pH 7.4], 2 M NaCl, 1 mM EDTA, 0.5% [vol/vol] Tergitol 15-S-9) and once with low salt buffer (5 mM Tris-HCl [pH 7.4], 1 mM EDTA, 0.1% [vol/vol] Tergitol 15-S-9). Beads were resuspended in 300 µL Trizol, and after vortexing and a 3 min incubation, 60 µL chloroform was added. This was followed by a centrifugation step at 14,000 × *g* for 5 min at 4°C. The upper, aqueous phase was transferred to a new tube, and the organic phase was discarded. The same beads were extracted a second time using Trizol and chloroform as previously described. The aqueous phases were combined and extracted with 1 volume of chloroform followed by centrifugation (14,000 × *g*, 5 min, 4°C). To the aqueous phase, 1 µL GlycoBlue (Invitrogen) and 900 µL 100% ethanol were added and thoroughly mixed. After incubation (10 min, RT), the RNA was precipitated (14,000 × *g*, 20 min, 4°C). The pellet was washed in 75% (vol/vol) ethanol (14,000 × *g*, 5 min, 4°C) and dried for 5–10 min.

For 3′ adapter ligation, the pellet was resuspended in 12.5 µM VRA3 Adapter ordered via IDT (/5Phos/GAUCGUCGGACUGUAGAACUCUGAAC/inverted dT/), incubated at 65°C for 20 s and placed immediately on ice. A ligation mix was added and the mixture was incubated for 4 h at 20°C. Subsequently, a second biotin enrichment was performed (see above). This was followed by a 5′ adapter ligation with VRA5 (CCUUGGCACCCGAGAAUUCCA) and a third biotin enrichment. The adapter-ligated RNA was reversely transcribed and a test PCR amplification for optimizing the cycle number was performed. After final amplification, the library was purified from an 8% polyacrylamide gel to remove adapter dimers. Concentrations were determined using the KAPA Library Quantification Kit (Roche) and libraries were sequenced on a NextSeqTM 550 system (Illumina) using a NextSeq 500/550 High Output Kit v2.5 (150 Cycles) single-end according to the manufacturer’s instructions.

The quality of the libraries was checked by the length distribution of the reads. Approximately 20 bases are protected from degradation by RNA polymerase between the RNA polymerase exit channel and the 3′ RNA end, so libraries below 20 nucleotides indicate degradation of the RNA after the nuclear run-on. A fragment size of 20–30 nucleotides is still considered as partial degradation (24). No RNA degradation could be detected in our libraries (Fig. S1).

### Bioinformatical analysis

PRO-seq reads were trimmed using cutadapt (25), which removes adapter sequences and short reads below 16 bp and aligned to *L. major* Friedlin2021 version 63 (26) using Bowtie2 (27). Positions were extracted using Bedtools (28). Counts were normalized by aligned nucleotides per sample. To incorporate and analyze the acetylation at Histone 3, the ChIP-on-chip data from (19) was used. From this experiment, positions for H3ac were determined by aligning the sequences derived from the microarray from replicate 2 of reference 19 to the Friedlin2021 genome using Bowtie2 (29) with parameter -k 5. BaseJ reads from reference 18 were aligned to the Friedlin genome using Bowtie (29) with parameter -k 5 and read coverage was calculated using in-house scripts. BaseJ and H3ac alignments were smoothed using a running average with a window of 1,000 bases. To incorporate the position of the centromere in *L. major,* the ChIP-seq data from reference 30 was used. Reads from the KKT1 (centromere) and the control sample were aligned to the reference genome of Friedlin2021 version 63 (26) using Bowtie2. The sam file was then sorted and converted into a bam file using samtools (31). Peaks were called using MACS2 (32) and the proportion of the pileup between sample and control plotted with R. PTUs were defined as stretches from one ATG to the last stop codon of consecutive genes in the same direction. dSSRs and cSSRs were defined as the regions between two divergent or convergent genes, respectively. 5′-Telomeres are the regions 5′ of a terminal gene to the end of the chromosome and 3′-telomeres are regions from 3′ end of a gene to the end of the chromosome. Head tail regions were defined by peaks of BaseJ and H3ac present within a PTU. Positions with Ns in the reference sequence and positions covered by RNA Pol I or RNA Pol III transcribed genes were not counted for PTUs, SSRs, and telomeric regions. Normalized counts of three biological replicates were summed up and averaged. Read counts in Fig. 2, 3, 5, and 6K through M and Fig. S2 to S6 are plotted logarithmically with pseudocounts.

## RESULTS

**Fig 1:**
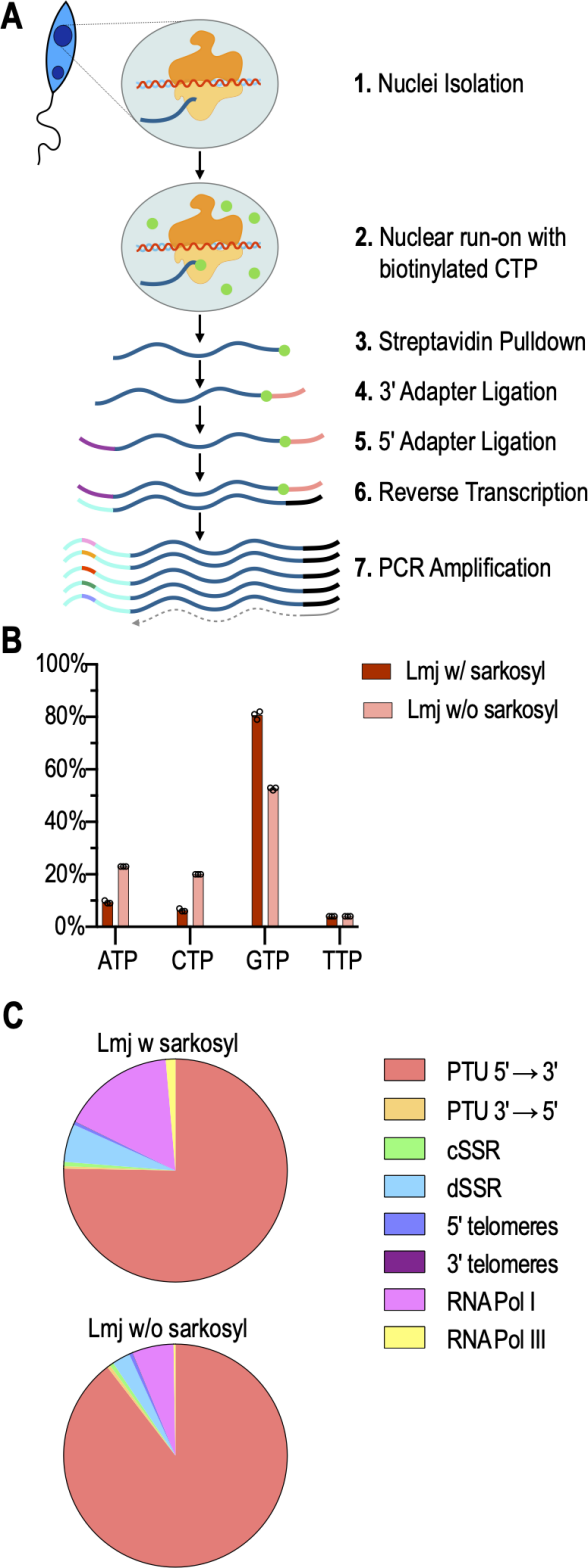
(A) Overview of PRO-seq: nuclei are isolated and an *in vitro* nuclear run-on reaction with biotinylated CTP is performed. After subsequent RNA isolation, the native RNA is purified with streptavidin beads. This is followed by 3′ and 5′ adapter ligation and reverse transcription. The libraries are then individually indexed by barcode PCR and sequenced with Illumina. (B) Percentage of bases at position 1 of reads in *L. major* with sarkosyl (Lmj w/ sarkosyl, dark red) and without sarkosyl (Lmj w/o sarkosyl, light red), *n* = 3. (C) The overall distribution of reads of *L. major* with and without sarkosyl in PTUs, SSRs, telomeres, and in RNA Pol I and III genes, *n* = 3.

The PRO-seq nuclear run-on transcription protocol as described in reference 23 allows a genome-wide mapping of RNA polymerase elongation complexes in the intact chromatin of isolated nuclei, reflecting positions, and densities of elongation complexes at the time of cell fractionation. To summarize the PRO-seq protocol, *L. major* promastigote cells were ruptured according to our established protocol (13), but replacing Triton X-100 with Tergitol. Aliquots of cryogenically stored nuclei were then added to a nuclear run-on mix, with or without the detergent sarkosyl, and containing ATP, GTP, UTP, and biotinylated CTP (Fig. 1A). This allowed RNA elongation to continue until the next GTP moiety in the non-coding strand DNA was encountered, at once labeling the nascent RNA with a 3′-terminal biotin tag and blocking further elongation. The nuclear run-on reaction was performed in the presence or absence of sarkosyl, an anionic detergent, that causes restarting of paused transcription complexes but not of terminated complexes (33, 34). Furthermore, only transcription-competent complexes can be detected with PRO-seq as the run-on reaction is dependent on the incorporation of nucleotides (34).

The nuclear run-on transcripts were then enriched by a streptavidin pull-down and further processed for Illumina sequencing by 3′- and 5′-adapter ligation, reverse transcription, and PCR amplification (Fig. 1A). The resulting libraries were subjected to NextSeq 550 (high output, 150 cycles) sequencing.

We performed PRO-seq with *L. major* logarithmic phase promastigotes in biological triplicates with or without sarkosyl. All libraries yielded comparable raw read numbers; however, 66% of reads derived from sarkosyl-treated nuclei remained after trimming with cutadapt, compared with 40% of reads from untreated nuclei (see Table S1). Moreover, when we analyzed the 5′ dNTPs of the sequencing reads from the sarkosyl libraries (Fig. 1B; Table S1), we found an 80% majority starting with a GTP, the complementary base of the biotin-CTP used to stop elongation, while only 53% of the reads from untreated nuclei started with GTP. This indicates that RNA polymerases are blocked more efficiently by biotin-CTP under sarkosyl, given that libraries originated from streptavidin-selected nascent RNAs.

We next aligned the reads to the *L. major* Friedlin genome (26) and performed a global RNA polymerase density analysis for PTUs in sense (5′→3′) and antisense (3′→5′) direction (according to the TriTrypDB annotation), for cSSRs and dSSRs, 5′- and 3′-telomeric regions, RNA polymerase I and RNA polymerase III genes (Fig. 1C). We find that under sarkosyl, i.e., mapping of all elongation complexes, including paused RNA polymerases, the proportions of elongating RNA polymerase I and III complexes at their dependent genes increases by over twofold compared with minus-sarkosyl reads, i.e., active elongation complexes only. A similar effect is seen for reads aligning to diverging SSRs. We also observe an overwhelming preference for RNA polymerases for the sense strand in the PTUs, confirming the concept of unidirectional, strand-specific transcription in the PTUs on a genome-wide level. The proportion of total aligned reads aligning in antisense to PTUs compared to all reads is in general very small (between 0.0052% and 0.0076%) and only around 0.001% show a high mapping quality and alignment outside of annotated genomic features (Table S3).

**Fig 2:**
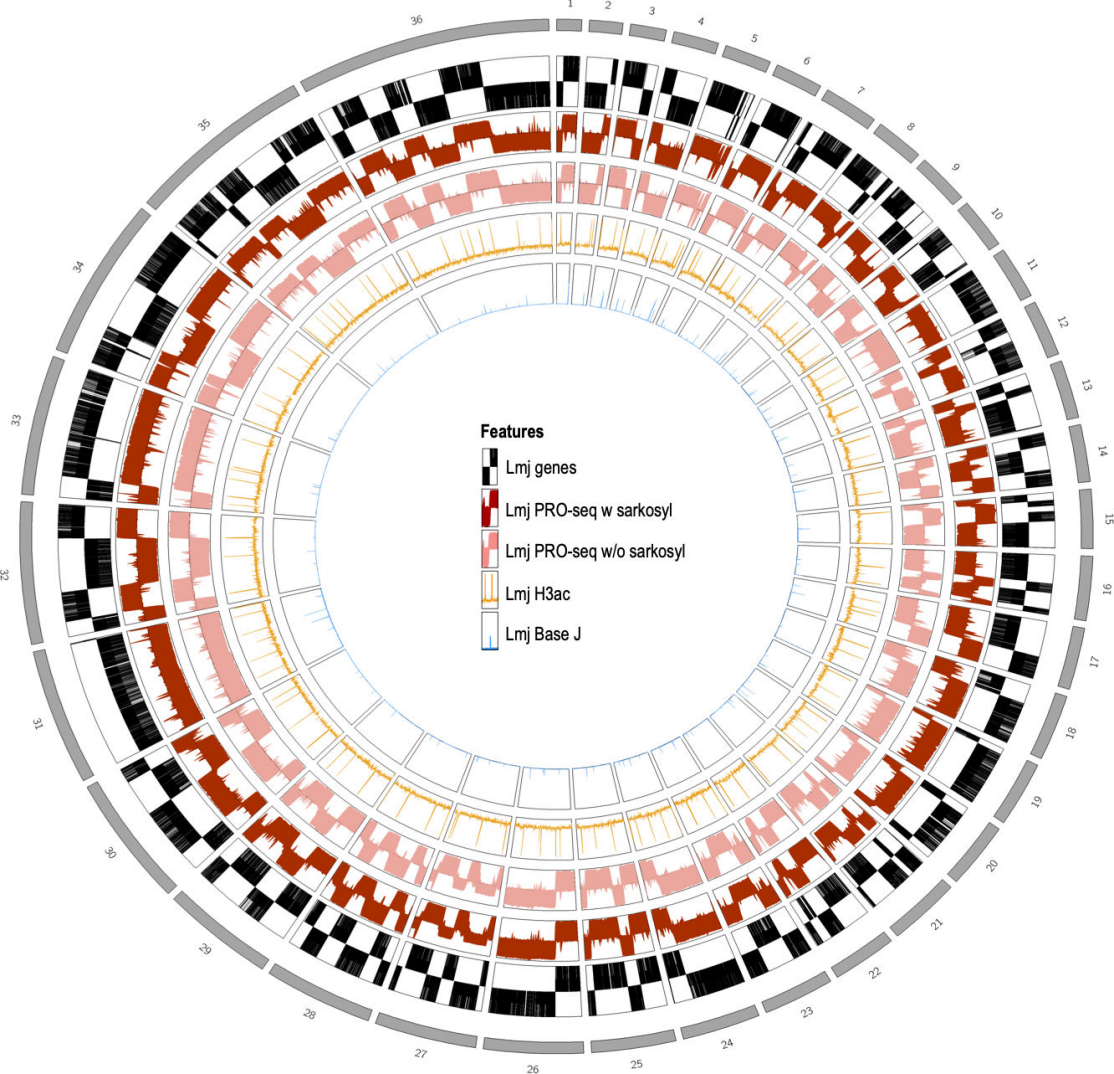
Circos plot. All 36 chromosomes of the *L. major* Friedlin 2021 genome are plotted in the outer ring (gray). Then genes follow, with untranslated regions (UTRs) plotted only halfway up and protein-coding sequences (CDSs) plotted over the entire length (black). Next plotted is a logarithmic representation of the PRO-seq data of *L. major* with sarkosyl (dark red) and without sarkosyl (light red), *n* = 3. ChIP-chip data from acetylated histone H3 (H3ac) (19) follow in orange, and ChIP-seq data from glucosylated hydroxymethyluracil (BaseJ) (18) follow in blue. H3ac and BaseJ were aligned to the *L. major* Friedlin 2021 genome (26).

The mapped positions of the reads are shown in the form of a whole-genome Circos plot for all 36 chromosomes of *L. major* (Fig. 2) and in linear form by chromosomes (Fig. S2). From the outside to the inside, the Circos plot shows the chromosomes, the open reading frames according to the TriTrypDB, the PRO-seq read density with sarkosyl, the PRO-seq read density without sarkosyl, the relative prevalence of H3ac, taken from reference 19, and the relative prevalence of BaseJ nucleotides, taken from reference 18. The latter was added to analyze the impact of both chromatin modifications on transcription initiation and termination. Again, the highly selective, unidirectional transcription of PTUs is confirmed.

**Fig 3:**
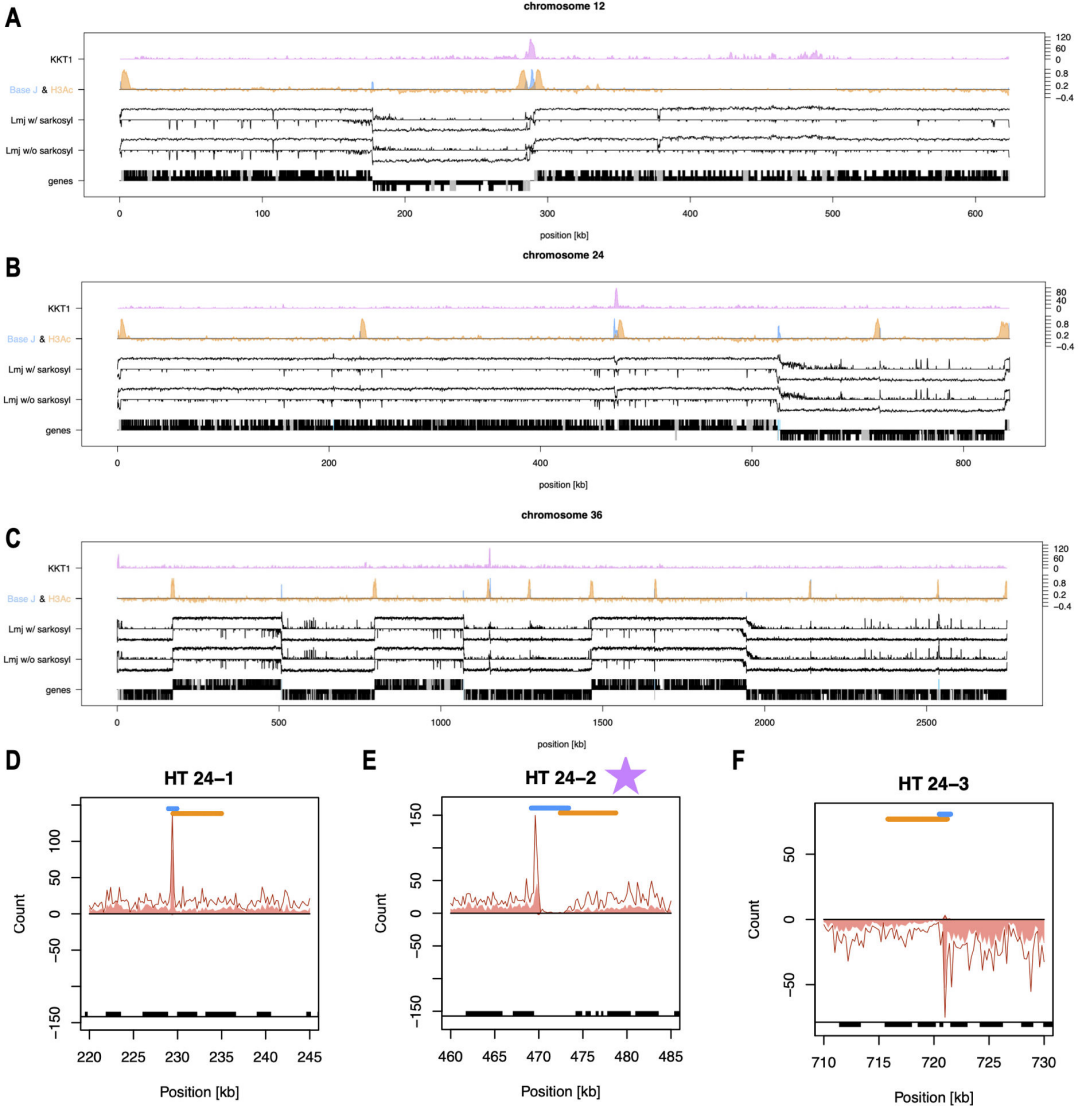
Chromosome-wide representation of PRO-seq data of *L. major* with sarkosyl (Lmj w/ sarkosyl) and without sarkosyl (Lmj w/o sarkosyl) on selected examples. PRO-seq data were plotted strand specifically and displayed logarithmically, *n* = 3. ChIP-Seq data from centromeres (KKT1) (30) is plotted in purple, ChIP-chip data from acetylated histone H3 (19) is plotted in orange, and ChIP-seq data from base J (18) is plotted in blue, each normalized within chromosomes. The bottom row shows genes (black), with UTRs plotted half-length and CDS plotted full-length. Non-coding RNAs are plotted in gray and RNA Pol III genes are in turquoise. Examples shown are chromosome 12 (A), chromosome 24 (B), and chromosome 36 (C). (D, E, F) Three head-tail regions on chromosome 24 are shown. Distribution of normalized PRO-seq reads (mean of three replicates) of samples with sarkosyl are plotted as a dark red line and samples without sarkosyl are plotted as a light red background. BaseJ peaks are shown in blue and H3ac peaks in orange. Genes are plotted as a black box at the bottom of the plots. The purple star indicates a centromere is present.

For a better understanding of transcription on a genome-wide scale, we displayed the PRO-seq reads in a higher resolution (Fig. 3; Fig. S2), allowing us to analyze interrupted transcription in head-tail regions marked by BaseJ (18) and H3ac (19). We further included the position of the centromeres from Garcia-Silva et al. to analyze transcription patterns in those (30).

In chromosome 12 (Fig. 3A), transcription initiation in dSSRs matches H3ac peaks at a 5′ telomere and, in a double peak, at a divergent SSR. Between the twin H3ac peaks, two peaks of BaseJ abundance can be observed, likely in conjunction with the centromere that is located in that dSSR (purple). Chromosome 24 consists of two PTUs and shows three PTU internal BaseJ peaks followed each by H3ac peaks, which is the marker for head-tail regions (Fig. 3B). To analyze the head-tail regions in more detail, we displayed them in a higher resolution in Fig. S11. We observe a concomitant drop of RNA polymerase density in our PRO-seq data at position ~470 k on chromosome 24, which overlaps with the centromere (Fig. 3B and E). On chromosome 24, there are two more PTU internal peaks of BaseJ and H3ac, but RNA polymerase density does not decrease or decrease only slightly (but does not reach zero over a longer region as in centromeric head-tail regions) (Fig. 3D and F). However, we do observe a high, sharp peak of RNA polymerase surrounded by low RNA polymerase density at these non-centromeric head-tail regions (and sometimes at centromeric head-tail regions), which could indicate that BaseJ and H3ac act as exit and entry points for RNA polymerases. On chromosome 36, BaseJ and H3ac spikes are again only associated with localized interruptions of unidirectional transcription at the centromere and otherwise correspond to spikes of polymerase activity on the opposite strand of a non-coding RNA, tRNA, or not annotated features (Fig. 3C). We conclude that indeed, chromatin modifications correlate with transcription initiation and termination at SSRs and that previously mapped PTUs contain short regions of termination and reinitiation at centromeres. However, the presence of BaseJ and H3ac only results in zero RNA polymerase occupancy within a PTU when a centromere is present. In a non-centromeric head-tail region, RNA polymerase occupancy does not seem to stop completely but we do observe a sharp accumulation of RNA polymerase that could indicate termination and reinitiation of transcription in these regions. This observation is true for all 36 chromosomes (Fig. S2 and S11).

**Fig 4:**
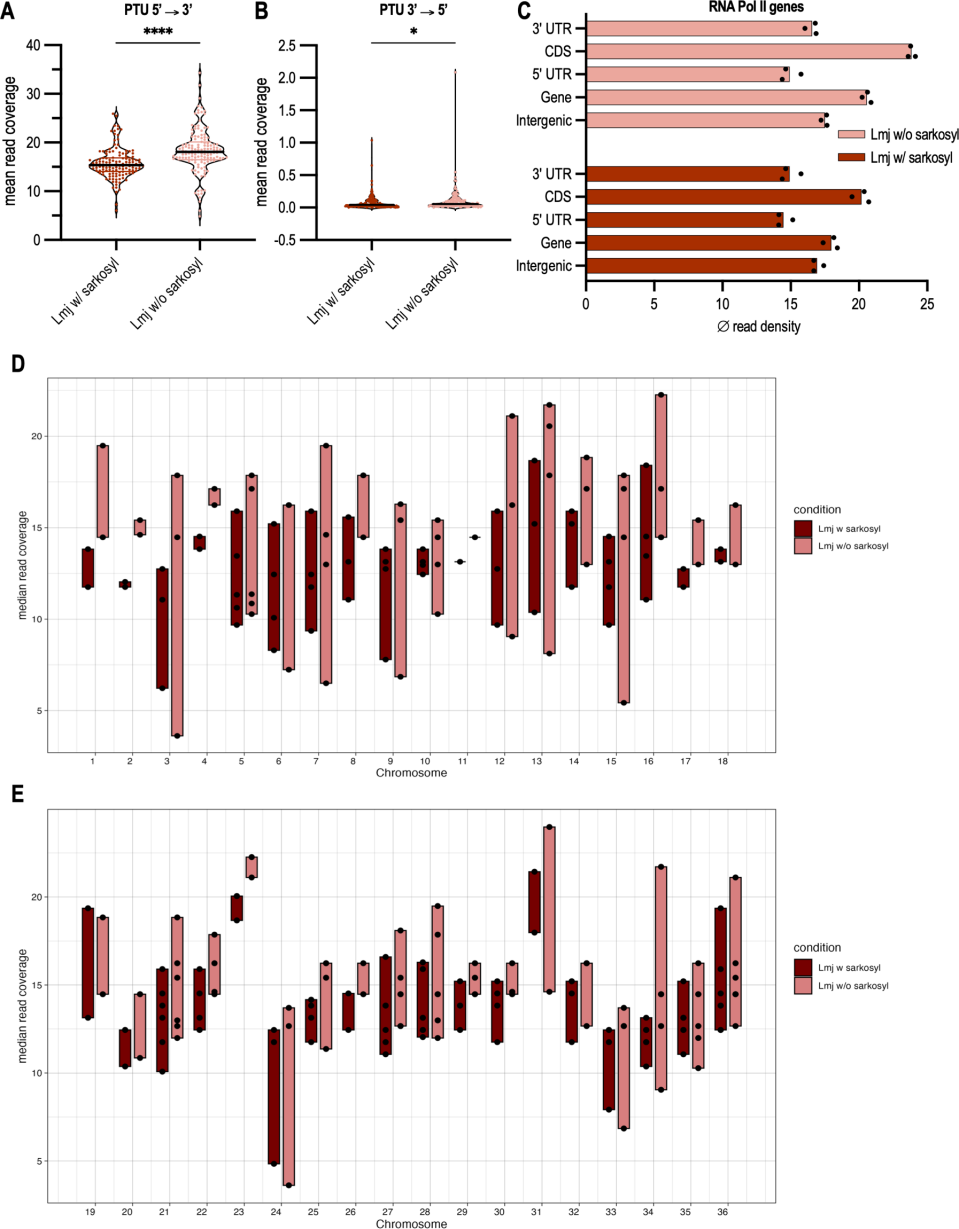
Read occupancy at PTUs. (A and B) The average occupancy of normalized PRO-seq reads within PTUs (*n* = 132) in sense (5′→3′) (A) and antisense (3′→5′) (B) direction. Each dot represents the mean read coverage over one PTU of three biological replicates of either *L. major* with sarkosyl (Lmj w/ sarkosyl, dark red) or without sarkosyl (Lmj w/o sarkosyl, light red). Significance was tested using a paired Student’s *t*-test; ****, *P* ≤ 0.0001; *, *P* ≤ 0.05. (C) Mean read coverage of normalized PRO-seq reads in RNA Pol II transcribed PTUs split by genes (UTR + CDS), 5′ UTRs, CDS, 3′ UTRs, and intergenic regions within PTUs. The regions were summed and the median over the entire regions in three biological replicates of *L. major* with sarkosyl (dark red) and without sarkosyl (light red) is shown. Each dot represents one biological replicate. (D and E) Read density of PRO-seq reads in PTUs across all 36 chromosomes of *L. major*. Each dot represents the median read coverage of one PTU on that chromosome, *n* = 3. Dark red boxes display samples with sarkosyl and light red boxes without sarkosyl. Chromosomes 1–18 are shown in panel D, and chromosomes 19–36 are shown in panel E.

Overall, the sense reads outnumber the antisense reads by over two orders of magnitude, both with and without sarkosyl (Fig. 4A and B). The PTUs show a slightly different level of RNA polymerase occupancy with a median sense read count between 3.5 and 21 for samples with sarkosyl and 1 and 24 for samples without sarkosyl (Fig. 4A). These differences are consistent within all three biological replicates (Table S4). A closer look at single PTUs reveals that on one and the same chromosome, small variations in RNA synthesis rates can be observed (Fig. 4D and E). However, the highest difference for the most varying PTUs is around 15. We displayed the PTUs by chromosome, as *Leishmania* often shows chromosomal aneuploidies, e.g., chromosome 31 being tetraploid. Yet, even on chromosome 31, the PTUs are transcribed at different levels. In general, it can be observed that some chromosomes transcribe their PTUs at similar levels, e.g., chromosomes 2, 4, 17, 18, 20, 23, 25, 26, 29, and 30, while some chromosomes show a small difference in read density at the PTUs, e.g., 3, 7, 12, 15, and 24. We also tested whether the length of a PTU or the number of genes inside a PTU has an influence on the PRO-seq coverage. PTUs with a low PRO-seq read count generally also show a low number of genes with the 11 lowest PTUs all containing less than four genes (Fig. S7). However, PTU 05–5, 10–1, and 31–2 also have three or four genes and do not differ from the average PTU median read coverage (Table S4). Also, the location of the PTU, if next to a centromere or telomere could have an influence on the mean read coverage of PRO-seq reads. We therefore analyzed if a PTU is next to a telomere or if a centromere was occurring within the PTU, or the subsequent cSSR or dSSR, and if this influences the transcription. Telomeric PTUs generally contain less than three genes and often but not always show a low RNA polymerase occupancy in the neighboring PTU. A centromeric location of the PTU does not influence the level of transcription on the following PTU (Fig. S7).

We also compared PRO-seq read alignment between the 5′ and 3′ untranslated regions (UTRs) and the protein-coding sequences (CDS), but also between mRNA coding regions (genes) and intergenic regions. The results (Fig. 4C) confirm the visual impression that transcription proceeds through intergenic regions with little if any drop in polymerase density. However, a ~25% drop in alignment frequency between protein-coding sequences and the UTRs was observed. As the PRO-seq data only covers sequences ending with a C, we analyzed the GC content in CDS and UTRs. We observe a slight decrease in GC content, with an average GC content of 62.5% in CDS and 56.6% in UTRs. Nevertheless, the difference we see in RNA polymerase occupancy is still much higher with a 25% difference. Further analysis on an individual gene level shows a few outliers, but the RNA polymerase occupation is, in general, higher at CDS (Fig. S8; Table S5). The top two outliers are proteophosphoglycan *ppg1* and *ppg3,* which are repeat-containing genes (35). Yet, not all genes show a higher RNA polymerase density at CDS, some genes also show a higher read density at UTRs.

**Fig 5:**
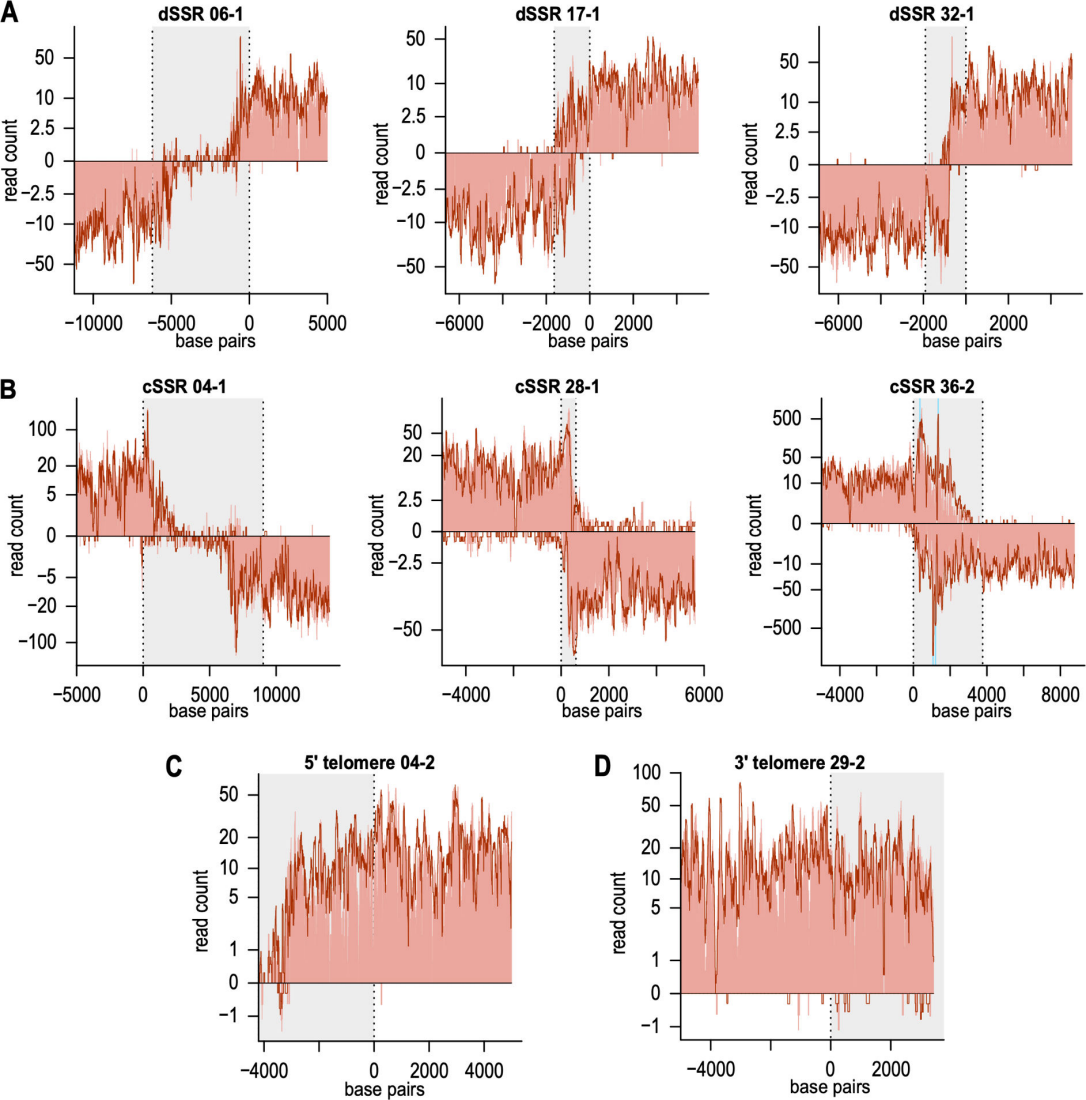
Distribution of normalized PRO-seq reads of *L. major* with and without sarkosyl at transcription initiation and termination sites. (A) Transcription initiation in divergent strand switch regions (dSSR 06-1, 17-1, and 32-1). dSSRs are highlighted in gray with the dashed lines representing the start of the PTUs. Normalized PRO-seq reads of *L. major* with sarkosyl are plotted logarithmically as a dark red line and reads of *L. major* without sarkosyl as light red background. The first 5,000 bp of each PTU are shown, *n* = 3. (B) Transcription termination within convergent SSRs (cSSR 04-1, 28-1, 36-2). cSSRs are shown in gray and the dashed lines represent the stop of the PTU with the last 5,000 bp of the PTU shown. The turquoise background shows RNA Pol III genes (cSSR 36-2), *n* = 3. (C and D) Transcription initiation in 5′ telomeres (C) and transcription termination in 3′ telomeres (D) are shown. The gray background represents the telomeric region with the dashed line indicating the start or stop of the PTUs. The first 5,000 bp of the PTUs are plotted, *n* = 3.

Next, we took a closer look at the strand switch regions to localize transcription initiation and termination regions. In Fig. 5A, we present RNA polymerase densities in 3 prototypic of 58 diverging SSRs. While in dSSR6, a central part is virtually devoid of RNA polymerase complexes, occupancy of the two DNA strands overlaps significantly in dSSR22, while in dSSR46, transcription initiates head-to-head. For RNA polymerase occupancy maps of all dSSRs see Fig. S3, information about the positions can be found in Table S2.

Likewise, the occupancy patterns of cSSRs are also diverse. cSSR2 shows a clear gap of polymerase occupancy (Fig. 5B), while PTUs in cSSR25 are tail-to-tail. An occupancy overlap on both DNA strands can be seen in cSSR37. The only exception in transcription termination is cSSR 26, where transcription only terminates after 15–20 kb (Fig. S9). At this cSSR, a BaseJ abundance peak is absent (Fig. S2). The occupancy patterns for cSSRs 1 to 38 can be viewed in Fig. S4.

We further analyzed if a centromere present in a dSSR or cSSR has an influence on RNA polymerase density (Fig. S10; Tables S6 and S7). In cSSRs, a centromere seems to lead to lower PRO-seq read coverage, whereas in dSSR, a centromere has no influence.

We also analyzed fifteen 5′ telomeric transcription start regions (Fig. S5) and 56 3′ telomeric transcription termination regions (Fig. S6). The example of 5′ telomere 3 (Fig. 5C) which shows transcription starting very close to the 5′ end of the chromosome is representative of the rest (Fig. S5). Likewise, transcription proceeds to the very 3′ ends of the chromosomes in 3′ telomere 43 (Fig. 5D), again reflecting a general pattern (Fig. S6).

**Fig 6:**
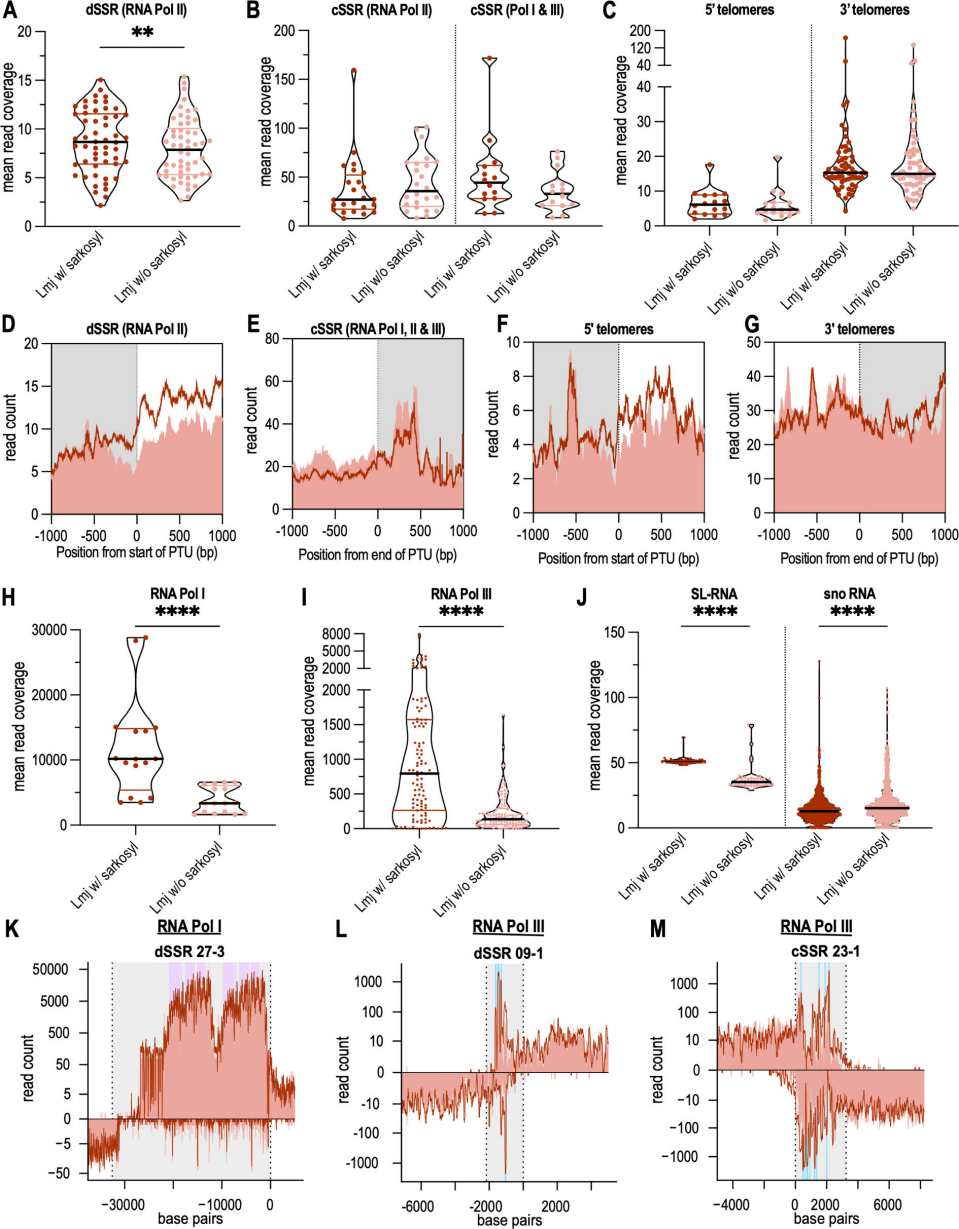
(A–C) Occupancy of dSSRs (A), cSSRs (B), 5′ telomeres, and 3′ telomeres (C) with normalized reads from *L. major* with sarkosyl (dark red) and without sarkosyl (light red). Each dot shows the mean read coverage in a dSSRs, cSSRs, 5′ telomeres, or 3′ telomeres, *n* = 3. dSSRs were only counted if there was no RNA Pol I or III gene locus within the dSSR (RNA Pol II, 55 out of 58 dSSRs). cSSRs were differentiated into cSSRs in which RNA Pol I and III gene loci are located (RNA Pol I and III, *n* = 14) and in which no additional gene loci are located and only RNA Pol II transcription stops here (RNA Pol II, *n* = 24). No additional RNA Pol I and III gene loci are located in 5′ telomeres (*n* = 16) and 3′ telomeres (*n* = 56). Significance was tested using a paired Student’s *t*-test; **, *P* ≤ 0.01; ns, *P* > 0.05. (D–G) Mean read coverages of normalized PRO-seq reads at the start of a PTU in dSSRs (D) and 5′ telomeres (F) and at the end of a PTU in cSSRs (E) and 3′ telomeres (G). The first and the last 1,000 bp around the start/end of a PTU are shown. *L. major* with sarkosyl is plotted as a dark red line and reads of *L. major* without sarkosyl as a light red background, *n* = 3. (H–J) Distribution of normalized PRO-seq reads within RNA Pol I and RNA Pol III transcribed genes and within non-coding RNAs. Occupancy of PRO-seq reads in RNA Pol I (*n* = 16) (H) and RNA Pol III (*n* = 107) (I) regions. Each dot shows the mean read coverage of three biological replicates from *L. major* with sarcosyl (dark red) and without sarcosyl (light red) in an RNA Pol I or III transcribed region. Significance was tested using a paired Student’s *t*-test; ****, *P* ≤ 0.0001. (J) Occupancy of the spliced leader RNAs (SL-RNA, *n* = 53) and small nucleolar RNAs (sno RNA, *n* = 1128) with PRO-seq reads. Significance was tested using a paired Student’s *t*-test; ****, *P* ≤ 0.0001; *n* = 3. (K) RNA Pol I gene locus of 5.8S, 18S, 28S rRNA within dSSR 28-1. The SSR is shown in gray and the dashed lines represent the start of the PTU with the first 5,000 bp of the PTU shown. The purple background shows RNA Pol I genes. Normalized PRO-seq reads of *L. major* with sarkosyl are plotted logarithmically as a dark red line and reads of *L. major* without sarkosyl as a light red background, *n* = 3. (L and M) RNA Pol III genes are shown within one dSSR and one cSSR. The plot is the same as in panel K, but for the cSSR the last 5,000 bp of the PTU are shown. The turquoise background shows RNA Pol III genes. Three biological replicates of either *L. major* with or without sarkosyl are shown.

Lastly, we looked at RNA polymerase pausing based on the sarkosyl-induced restarting (Fig. 6). When analyzing PTUs (Fig. 4A, D, and E; Table S4), we generally observed a higher read occupancy in samples without sarkosyl, i.e., active transcription elongation complexes only, compared to run-on reactions with sarkosyl, i.e., paused and active transcription elongation complexes, suggesting that sarkosyl is not in general boosting transcription but rather acting specifically in *Leishmania* as previously described in other eukaryotes (33, 36). Looking at dSSRs in general, RNA polymerase II complexes show a moderate but significant occupancy increase of reads in nuclear run-on reactions with sarkosyl (Fig. 6A). At higher resolution, RNA synthesis in the absence of sarkosyl is markedly lower at the start of the PTUs (Fig. 6D), suggesting paused RNA polymerase complexes at transcription initiation sites. The same is observed at the border region and the beginning of PTUs downstream of 5′-telomeric start sites (Fig. 6F), but no significant reduction was noticed within the 5′-telomeres (Fig. 6C). However, there is no distinct peak of paused RNA polymerase complexes in dSSRs and 5′ telomeres; rather, longer stretches are affected. In regions of transcription termination, i.e., cSSRs, and 3′ telomeres, no RNA polymerase II pausing is observed (Fig. 6B, E, and G).

Paused RNA polymerases can be seen in genes transcribed by RNA polymerase I, i.e., large ribosomal RNA genes (28S, 18S, and 5.8S rRNA; Fig. 6H and K), or RNA polymerase III, (tRNAs, 5S rRNA; Fig. 6I, L, and M). Spliced leader RNA (SL-RNA) genes, transcribed by RNA polymerase II, are also occupied by a sizable percentage of paused transcription complexes, while the functionally related small nucleolar RNA genes are not sites of paused RNA polymerases (Fig. 6J). The products of these genes, ribosomal RNAs and tRNAs, spliced leader RNAs, and snoRNAs are all known to be crucial for protein synthesis and cell proliferation.

## DISCUSSION

### PRO-seq to analyze active transcription in Leishmania

In this study, we provide a genome-wide view of transcription in *L. major* using PRO-seq analysis. Our aim was to monitor ongoing active transcription by mapping RNA polymerase elongation complexes within PTUs, telomeres, and strand switch regions and to identify the transcription initiation and termination sites on all 36 chromosomes. The information derived from PRO-seq pertains to an earlier stage of gene expression than the commonly applied RNA-seq analyses, which analyze the mRNA steady-state levels which are the result of transcription followed by RNA splicing minus RNA degradation (6) and are therefore not a measure of transcription.

To gain insight into active transcription there are a number of sequencing methods available. PRO-seq is a further development of GRO-seq (Global Run-On sequencing) (37), in which bromouridine-labeled nascent RNAs are purified via affinity chromatography and then sequenced. However, GRO-seq does not offer a nucleotide-precise resolution like PRO-seq, but only an accuracy of about ±20 nt. Another alternative to PRO-seq is Native Elongating Transcript sequencing, in which the nascent RNAs are purified and sequenced via immunoprecipitation of the RNA Pol II elongation complex (38). Nevertheless, immunoprecipitation can also lead to precipitation of other complexes due to non-specific antibody binding, and, in addition, the nascent RNAs must be at least 18 nt in length. Since we are interested in transcription by all RNA polymerase classes, we opted for PRO-seq. To our knowledge, this study presents the first genome-wide PRO-seq analysis of transcription in Trypanosomatida.

PRO-seq was first used to analyze transcription initiation and pausing around the *Drosophila melanogaster* Hsp70 promoter (39) and had to be adapted for use in *Leishmania*. In *Leishmania*, capping of mRNA occurs during the process of trans-splicing (6), therefore no cap is present during the elongation of nascent RNA, making the decapping step in the original protocol unnecessary (Fig. 1A). The nuclear run-on reaction was performed with a single biotin-labeled NTP, as the main purpose was to map and quantify RNA polymerase elongation complexes for which single-nucleotide accuracy was not needed. We picked biotinylated CTP due to the GC richness (>60%) of the *Leishmania* genome.

Since the insertion of a biotinylated NTP causes termination of transcription, the start of each read should be a guanine base, as sequencing was performed from the 3′ end making the reads reverse complementary. Kwak et al. (39) were able to show in their experiment that the substrate-specific base was incorporated by 67% to 88% in the presence of sarkosyl. In our analysis, we achieved 80% and 53% incorporation of a biotinylated CTP performing the run-on with or without sarkosyl, respectively (Fig. 1B). We conclude that sarkosyl not only leads to the restarting of transcription by paused RNA polymerases but also prevents read-through transcription over incorporated biotin-CTPs.

### Transcription of polycistronic transcription units

The first supporting evidence for polycistronic transcription in trypanosomes came from northern blot analyses when RNA species encoded by intergenic regions of two different gene families were detected (40–42). Nuclear run-on analyses of gene loci (11–13, 43), a part of a chromosome (14), or full chromosomes 1 and 3 (15–17) also showed transcription in *Leishmania* to be polycistronic in the investigated loci and chromosomes. Wedel et al. provided in 2017 the first sequencing-based proof of polycistronic transcription in *T. brucei* by applying 5′-triphosphate-containing small RNA sequencing reflecting primary transcripts (8). Our PRO-seq data provide final proof for polycistronic transcription in *Leishmania* on a genome-wide scale (Fig. 2 and 3). PRO-seq reads are distributed within PTUs in a unidirectional, strand-specific manner on all 36 chromosomes of *L. major*. Coverage of reads in the sense direction is two orders of magnitude higher compared with the antisense direction (Fig. 4A and B). The first nuclear run-on analysis in *Leishmania,* which focussed on the beta-tubulin gene cluster, already showed a strand-specific transcription (44). Later, strand-specific transcription was confirmed in other nuclear run-on analyses (15, 17) and through strand-specific RNA-seq (20).

Since there are no defined gene-specific promoters for the genes transcribed by RNA Pol II in trypanosomes (with the exception of the SL gene locus), it was assumed for a long time that regulation takes place exclusively at the post-transcriptional level and that transcription is not regulated at the level of transcription initiation. Transcription elongation rates of PTUs remained unknown as sequencing-based methods mostly analyze mRNA abundance levels (6, 45). A newer study from 2022, however, used ChIP-seq with an antibody binding to RNA Pol II of *T. brucei* and identified potential promotor regions of polycistronic transcription initiation (9). It was also shown that sequence-specific promoters lead to different luciferase reporter activity in transient transfection assays, which was also found in a study by Wedel et al. analyzing GT-rich promoter sequences in reporter assays (8). Our PRO-seq analysis revealed some genome-wide differences in the occupancy of some PTUs with transcriptional elongation complexes (Fig. 4D and E; Table S4). Due to the aneuploidy for which Leishmania is known (46), we specifically analyzed PTUs located on the same chromosomes and found small differences in transcription levels for some chromosomes. These differences are sometimes correlated with a low number of genes in the PTU which often co-occurs with a telomeric location. A centromere does not influence RNA synthesis of the PTU.

Moreover, we observe a ~25% drop in RNA polymerase density in the 5′ and 3′ untranslated regions (Fig. 4C; Fig. S8). On an individual gene level, genes do not always have a higher RNA polymerase density at protein-coding sequences (Table S5). We have no ready explanation for this finding, but such variations between coding sequences and flanking regions were observed before for the HSP70 gene repeat (11).

### Transcription initiation and termination

Due to the lack of defined promoters and canonical transcription factors in trypanosomes (47) transcription initiation and termination remain poorly understood. As mentioned above, only two studies have identified sequence-specific promoters in *T. brucei*, one being a long GT-rich sequence (8) and the other a 75 bp promoter element (9). Here, we provide a genome-wide view of transcription initiation and termination sites in *L. major*. In prototypic Eukaryotes, initiation and termination of transcription are regulated by trans-acting protein factors, which *Leishmania* lacks, but also by chromatin conformation changes and epigenetic markers. Therefore, we incorporated the peaks of acetylated histone H3 (19) and BaseJ abundance (18), which are associated with transcription initiation and termination, respectively, in our analysis. In the dSSRs and cSSRs, our PRO-seq read alignment starts and stops mostly match the H3ac and BaseJ peaks (Fig. 2 and 3; Fig. S2). One exception is cSSR 26 on chromosome 28, where no BaseJ peak is present and transcription only drops slowly compared to other cSSRs (Fig. S9). This has been shown before by van Luenen et al. (18) where they used small RNA-Seq (18). They further show that the loss of BaseJ leads to read-through transcription, confirming that BaseJ is needed for transcription termination.

However, when a BaseJ peak (stop) followed by an H3ac peak (start) occurs within PTUs, we cannot always observe a complete drop in RNA polymerase density in our PRO-seq data. The only time we observe a complete drop over a longer region is when a centromere (30) is present at this position. At non-centromeric head-tail regions, a peak of RNA polymerase occupancy is present that may indicate a potential exit or entry point and therefore termination and reinitiation of transcription in these regions. If we compare these head-tail regions (Fig. S11) with the termination of transcription at cSSRs (Fig. 5B; Fig. S4) we observe that the stop of transcription in cSSRs does not always happen abruptly but RNA polymerase density is sometimes gradually reduced. Therefore, we can not exclude that at non-centromeric head-tail regions, an overlapping of RNA polymerases for the two potential transcription units occurs (one that is terminating and one that is initiating). Sometimes but not always, H3ac and BaseJ peaks at non-centromeric head-tail regions correlate with spikes on the antisense strand that overlap with RNA Pol III or potential non-coding genes. Some cSSRs also harbor RNA Pol III genes, but our PRO-seq reads cannot differentiate between RNA polymerase classes. To distinguish between those, inhibition of the run-on reaction with different concentrations of α-amanitin (48, 49) may be used in future analyses since discriminative inhibition of *Leishmania* RNA polymerase classes is established (17, 44).

In the 3′ telomere regions, there is not always a BaseJ peak; transcription usually continues until the end of the chromosome, resembling run-off transcription (50) (Fig. S6). In general, a couple of distinct patterns terminate transcription in cSSRs (Fig. S4). The same can be observed for transcription initiation in dSSRs and 5′ telomeres (Fig. S3 and S5).

In general, open chromatin conformation is important for the recruitment of RNA polymerases and other trans-acting protein factors in eukaryotes, with nucleosome positioning also playing a role (21). For *L. donovani* promastigotes, open chromatin could be shown for dSSRs and at RNA Pol I and III gene loci (22). Another study in *L. major* showed a well-positioned nucleosome at transcription initiation sites and around RNA Pol III gene loci, whereas in cSSRs a lower nucleosome occupancy was detected (21). Especially BaseJ and the involvement of a PJW/PP1 complex seem to play a role in the termination as mutations in different proteins that are part of this complex result in readthrough transcription in trypanosomes (20, 51–53). In addition, histone variants have been shown to play an important role. Histone variants cause nucleosomes to become more unstable, resulting in a more dynamic chromatin (54). The histone variant H3.V from *L. major* and *Leishmania tarentolae* co-localizes with BaseJ at transcription termination sites (52, 55), but the loss of H3.V alone did not cause termination of transcription (56). Furthermore, the histone variants H2A.Z and H2B.V co-localize with transcription initiation sites in *L. tarentolae*, whereas nucleosomes within PTUs usually consist of core histones and not histone variants (55). H2A.Z and H2B.V were also shown to overlap with transcription initiation in *T. brucei* (8, 52), while H3.V and H4.V are enriched at termination sites (54).

### RNA polymerase pausing

Promoter-proximal pausing has been ascribed a role in gene expression control and splicing in various eukaryotes and often occurs close to promoter regions and polyadenylation sites (36). It was especially shown to regulate transcription of the *hsp70* gene locus in *Drosophila melanogaster* in response to heat shock as it was proposed that paused RNA polymerase II after initiation can facilitate a faster response to environmental changes (39, 57). In trypanosomatids, there is some suggestion that pausing exists (8, 21, 58), so we decided to analyze pausing using nuclear run-on reactions with and without sarkosyl in our study. Trypanosomes, as early branching eukaryotes, contain all core RNA Pol II subunits but lack two subunits of RNA Pol I and one of RNA Pol III (59). The mechanism of promoter-proximal pausing of RNA Pol II after transcription initiation depends on NELF and DSIF in eukaryotes, but orthologues cannot be identified in trypanosomes (60, 61). Nevertheless, trypanosomes contain orthologues of the transcription elongation factor TFIIS, whose binding site is blocked by NELF so that the RNA polymerase cannot elongate (62). We observe a slightly higher read occupancy in regions of transcription initiation at dSSRs and 5′ telomeres, but no distinct peak was observed (Fig. 6A, D and F). In *Drosophila melanogaster*, some genes have been shown to have a focused peak for pausing, while others have a more dispersed pausing pattern (39). In *T. brucei*, a pronounced peak of 100–200 bp downstream of the transcription start on chromosome 9 was observed using primary transcription sequencing (8). Even if we do not see a distinct peak, we can exclude that sarkosyl generally leads to more transcription, as we observe a higher read occupancy within the PTUs without sarkosyl.

Furthermore, we observe a significant increase of reads within RNA Pol I and III transcribed gene loci in samples with sarkosyl indicating a release of paused RNA polymerase I and III complexes (Fig. 6H and I). Pausing has been described to support processing of rRNA co-transcriptionally via the control of RNA polymerase I progression in yeast as this is important for ribosome assembly (63–65). We conclude that we found further evidence for the existence of RNA polymerase pausing, although the mechanism of RNA polymerase pausing by RNA Pol II and especially for RNA Pol I and III in trypanosomes remains unclear. To further analyze pausing, a single-basepair resolution of the position of RNA polymerase complexes is necessary, which can be achieved by performing four nuclear run-on experiments using different biotinylated nucleoside triphosphates in each reaction. Also, investigating different environmental conditions, e.g., heat shock, would help to observe changes in the pausing pattern as we only analyzed *L. major* promastigotes under logarithmic growth conditions.

## Data Availability

All PRO-seq read files are made available at the Sequence Read Archive of NCBI under the link https://www.ncbi.nlm.nih.gov/bioproject/PRJNA1020779.

## SUPPLEMENTAL MATERIAL

Figure S2 mbio.02241-24-s0001.pdf Linear plot of PRO-seq reads against all 36 chromosomes of the *L. major* Friedlin 2021 genome.

Figure S3 mbio.02241-24-s0002.pdf Transcription starts at dSSRs.

Figure S4 mbio.02241-24-s0003.pdf Transcription stops at cSSRs.

Figure S5 mbio.02241-24-s0004.pdf Transcription starts at 5′-telomere ends.

Figure S6 mbio.02241-24-s0005.pdf Transcription at 3′-telomere ends.

Figure S11 mbio.02241-24-s0006.pdf Head tail regions of all 36 chromosomes.

Figures S1 and S7-S10 mbio.02241-24-s0007.pdf Genome-wide read alignments.

Legends mbio.02241-24-s0008.docx Supplemental figure legends.

Supplemental Tables mbio.02241-24-s0009.xlsx Tables S1–S7.

ASM does not own the copyrights to Supplemental Material that may be linked to, or accessed through, an article. The authors have granted ASM a non-exclusive, world-wide license to publish the Supplemental Material files. Please contact the corresponding author directly for reuse.
