# Supplementary material for: Genome-wide quantification of polycistronic transcription in Leishmania major
Source: mBio. 2024 Nov 25;16(1):e02241-24. doi: 10.1128/mbio.02241-24 (PMC11708010; doi:10.1128/mbio.02241-24)
Supplement: Figure S2 — Linear plot of PRO-seq reads against all 36 chromosomes of the L. major Friedlin 2021 genome. [file mbio.02241-24-s0001.pdf]

Figure S2: Linear plot of PRO-seq reads against all 36 chromosomes of the L. major Friedlin 2021 genome. From top to bottom, the plots show the centromere (purple) (Garcia- Silva et al., 2017) BaseJ (blue) (van Luenen et al., 2012) and H3ac (orange) (Thomas et al., 2009), PRO-seq read densities from nuclei without sarkosyl (upper and lower strands), PRO-seq read densities from nuclei without sarkosyl (upper and lower strands), and L. major genes with UTRs at half-height and CDSs at full height. Genes in grey: non coding RNAs, genes in blue: RNA Pol III transcribed genes.

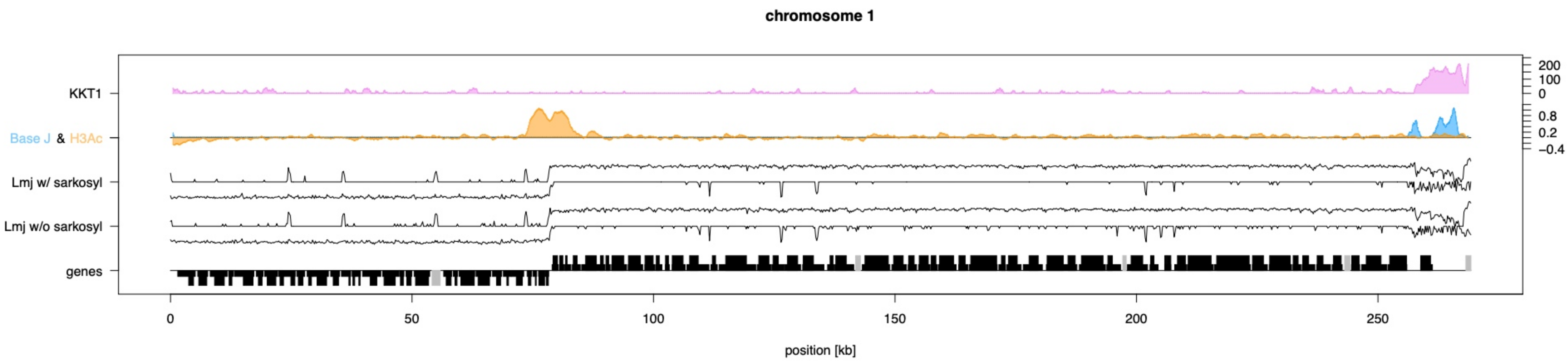

chromosome 1
KKT1
Base J & H3Ac
Lmj w/ sarkosyl
Lmj w/o sarkosyl
genes
200
100
0
0.8
0.2
−0.4
0
50
100
150
200
250
position [kb]

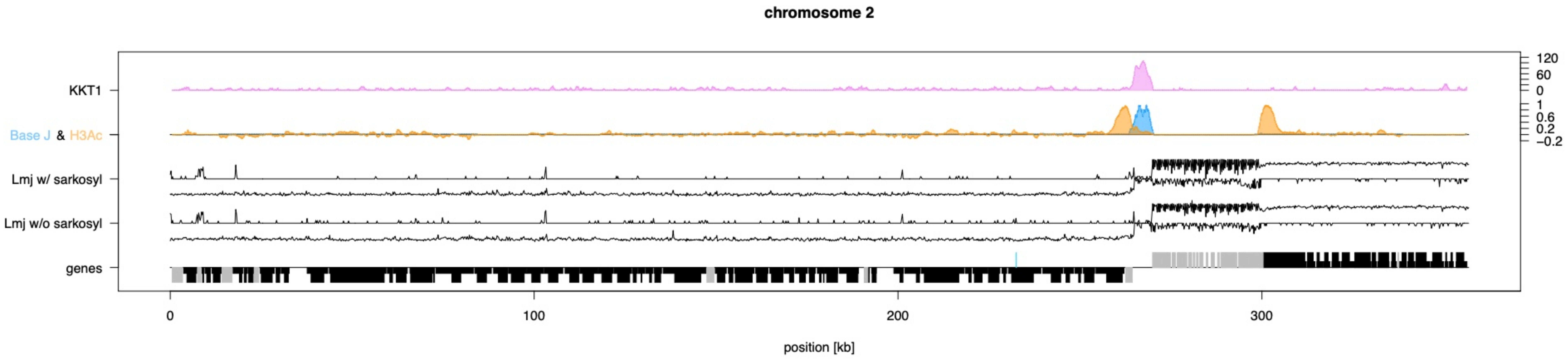

chromosome 2
KKT1
Base J & H3Ac
Lmj w/ sarkosyl
Lmj w/o sarkosyl
genes
120
60
0
1
0.6
0.2
−0.2
0
100
200
300
position [kb]

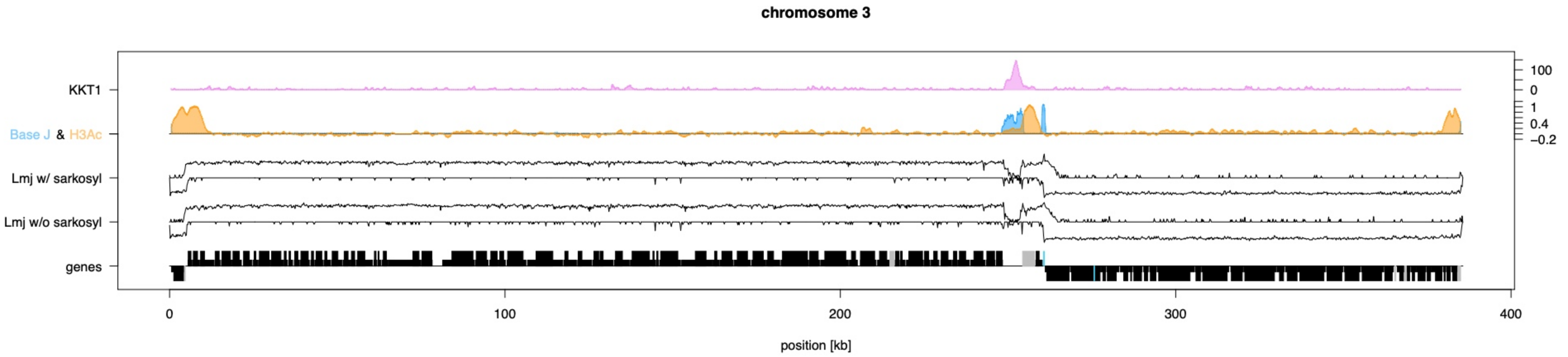
chromosome 3
KKT1
Base J & H3Ac
Lmj w/ sarkosyl
Lmj w/o sarkosyl
genes
100
0
1
0.4
−0.2
0
100
200
300
400
position [kb]

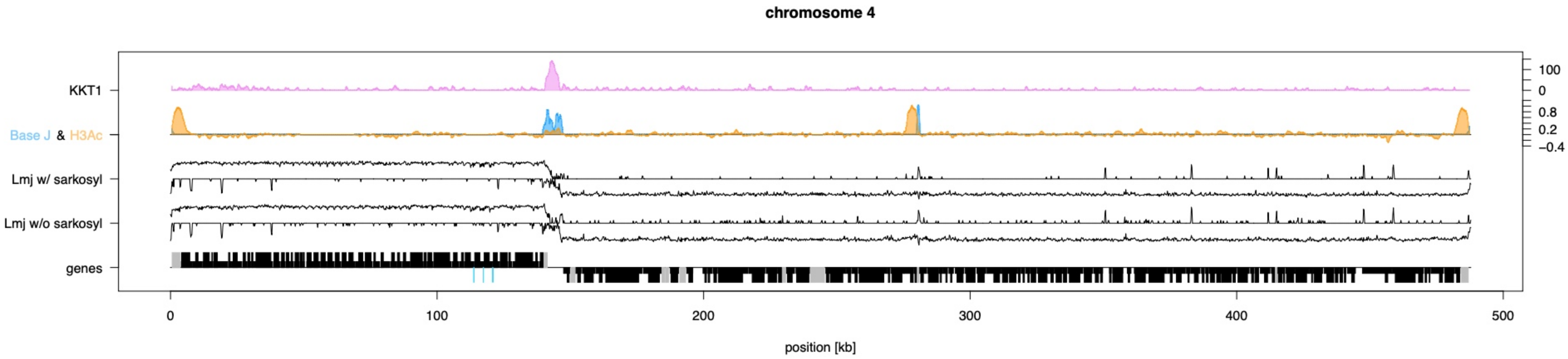
chromosome 4
KKT1
Base J & H3Ac
Lmj w/ sarkosyl
Lmj w/o sarkosyl
genes
100
0
0.8
0.2
−0.4
0
100
200
300
400
500
position [kb]

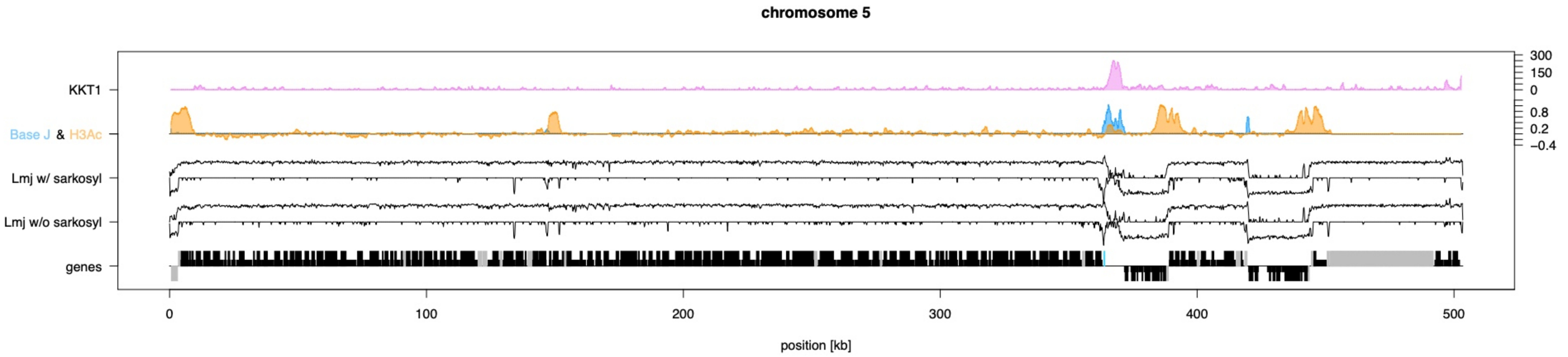

chromosome 5
KKT1
Base J & H3Ac
Lmj w/ sarkosyl
Lmj w/o sarkosyl
genes
300
150
0
0.8
0.2
−0.4
0
100
200
300
400
500
position [kb]

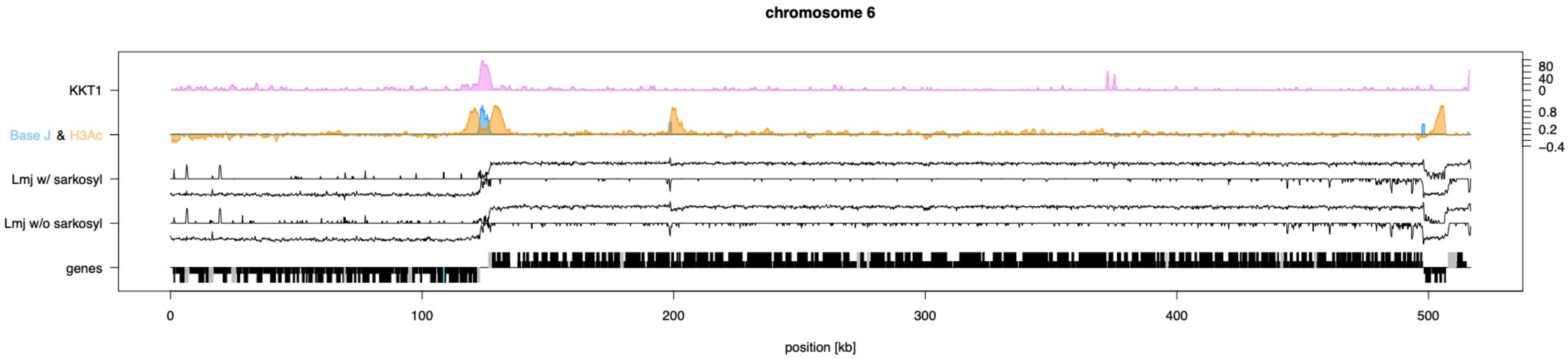

chromosome 6
KKT1
Base J & H3Ac
Lmj w/ sarkosyl
Lmj w/o sarkosyl
genes
80
40
0
0.8
0.2
−0.4
0
100
200
300
400
500
position [kb]

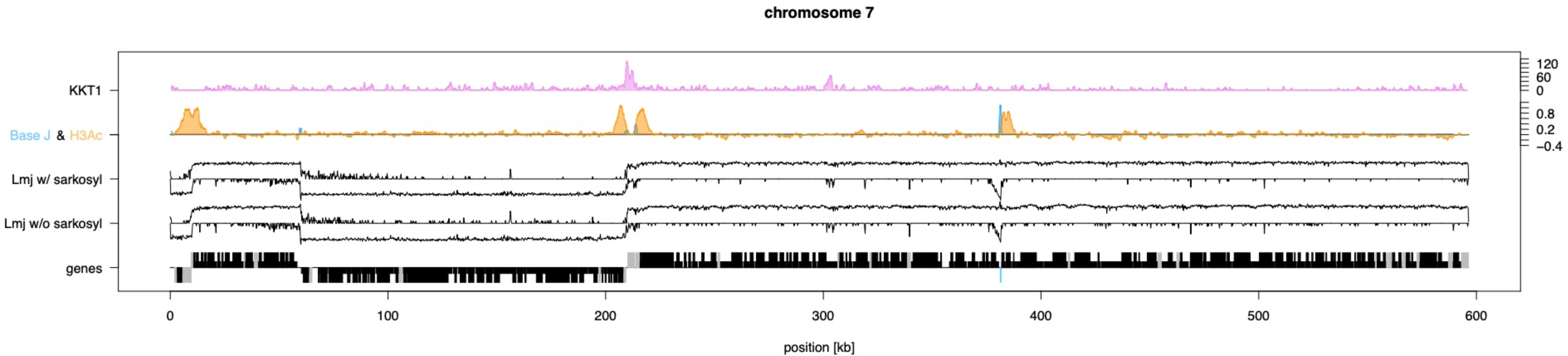

chromosome 7
KKT1
Base J & H3Ac
Lmj w/ sarkosyl
Lmj w/o sarkosyl
genes
120
60
0
0.8
0.2
−0.4
0
100
200
300
400
500
600
position [kb]

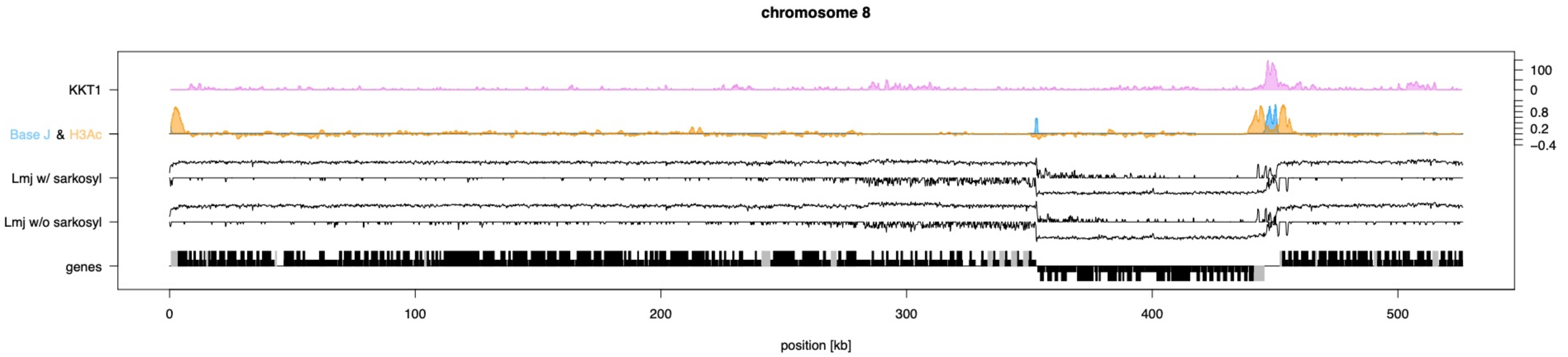

chromosome 8
KKT1
Base J & H3Ac
Lmj w/ sarkosyl
Lmj w/o sarkosyl
genes
100
0
0.8
0.2
−0.4
0
100
200
300
400
500
position [kb]

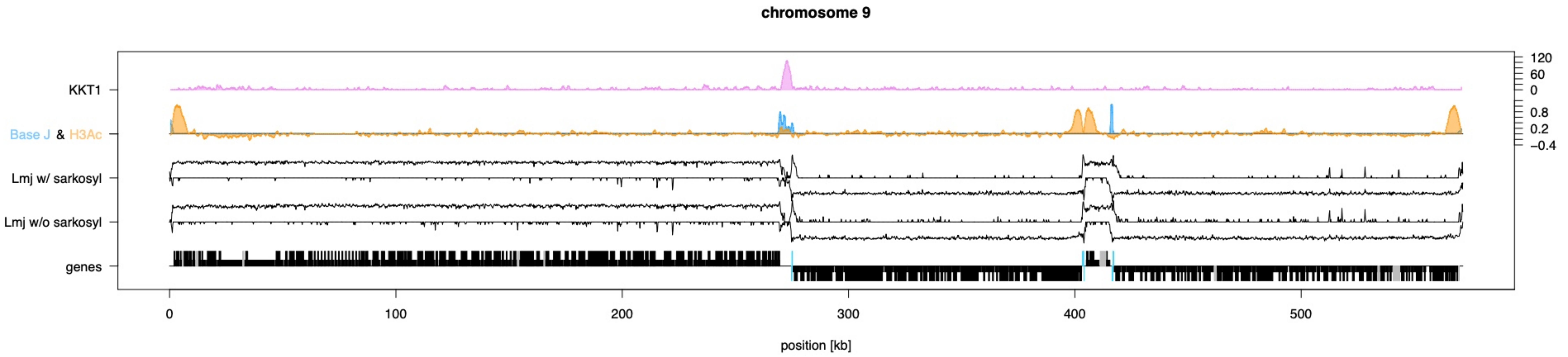
chromosome 9
KKT1
Base J & H3Ac
Lmj w/ sarkosyl
Lmj w/o sarkosyl
genes
120
60
0
0.8
0.2
−0.4
0
100
200
300
400
500
position [kb]

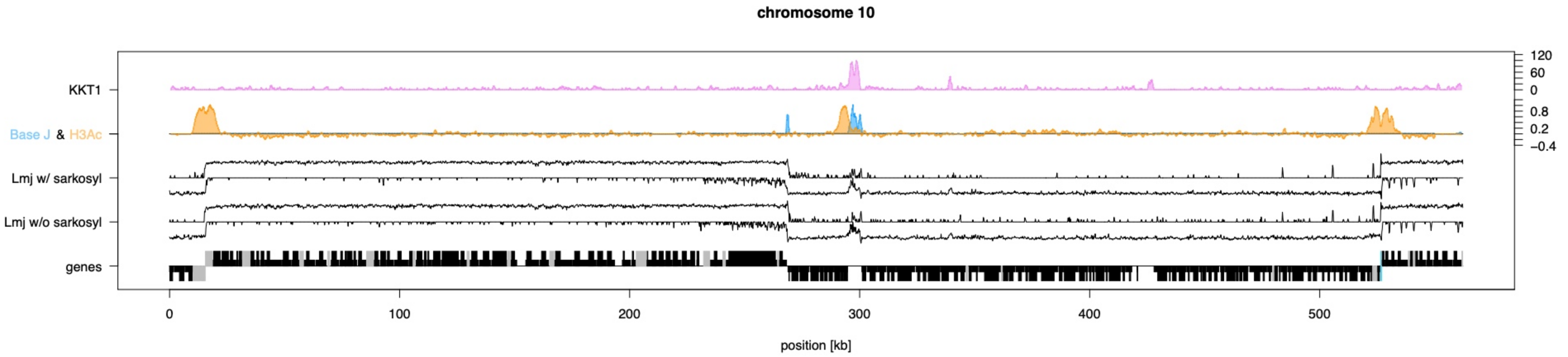

chromosome 10
KKT1
Base J & H3Ac
Lmj w/ sarkosyl
Lmj w/o sarkosyl
genes
120
60
0
0.8
0.2
−0.4
0
100
200
300
400
500
position [kb]

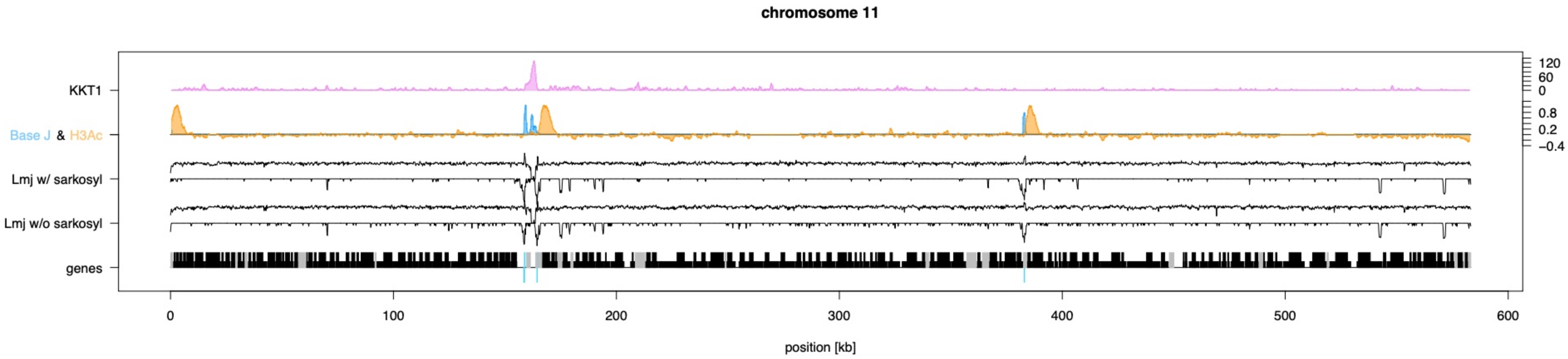
chromosome 11
KKT1
Base J & H3Ac
Lmj w/ sarkosyl
Lmj w/o sarkosyl
genes
120
60
0
0.8
0.2
−0.4
0
100
200
300
400
500
600
position [kb]

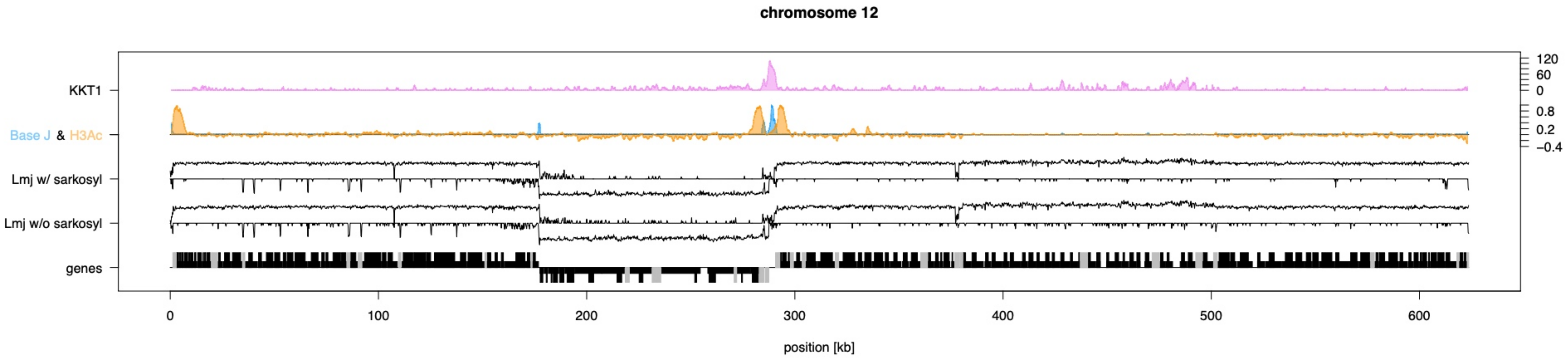

chromosome 12
KKT1
Base J & H3Ac
Lmj w/ sarkosyl
Lmj w/o sarkosyl
genes
120
60
0
0.8
0.2
−0.4
0
100
200
300
400
500
600
position [kb]

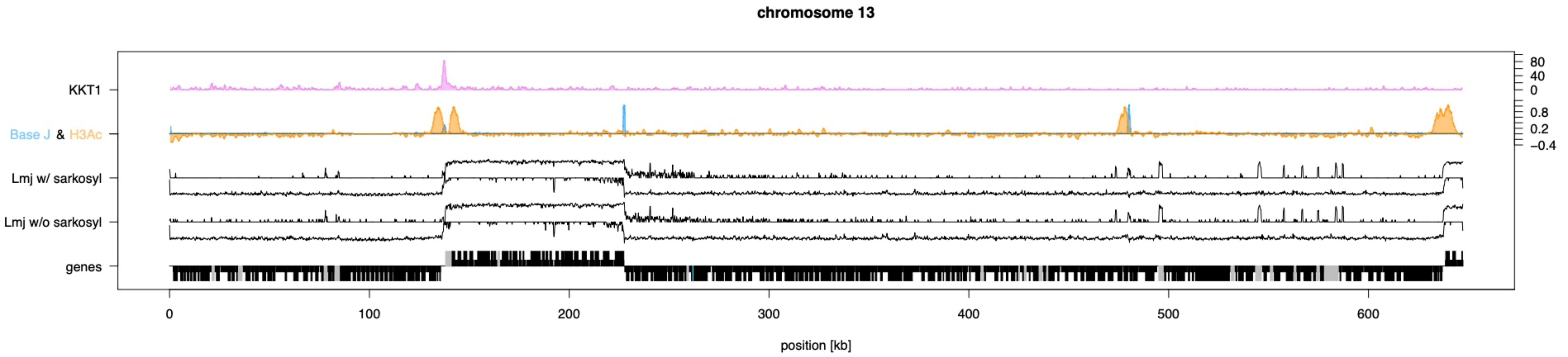
chromosome 13
KKT1
Base J & H3Ac
Lmj w/ sarkosyl
Lmj w/o sarkosyl
genes
80
40
0
0.8
0.2
−0.4
0
100
200
300
400
500
600
position [kb]

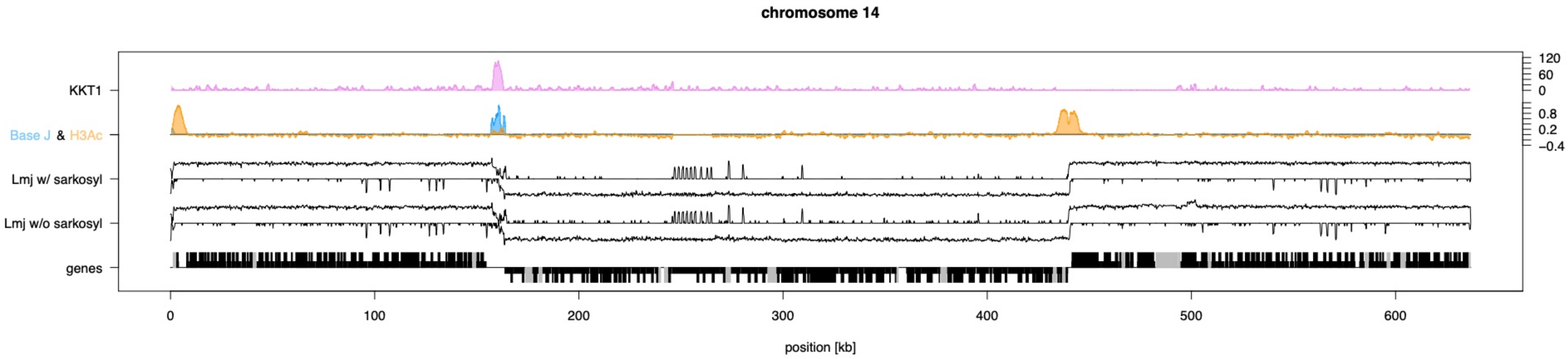

chromosome 14
KKT1
Base J & H3Ac
Lmj w/ sarkosyl
Lmj w/o sarkosyl
genes
120
60
0
0.8
0.2
−0.4
0
100
200
300
400
500
600
position [kb]

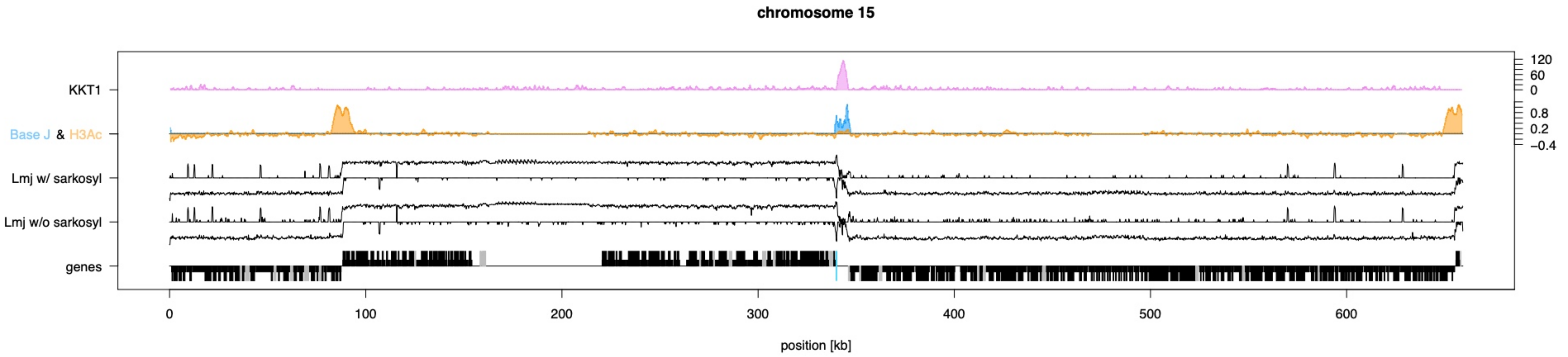

chromosome 15
KKT1
Base J & H3Ac
Lmj w/ sarkosyl
Lmj w/o sarkosyl
genes
120
60
0
0.8
0.2
−0.4
0
100
200
300
400
500
600
position [kb]

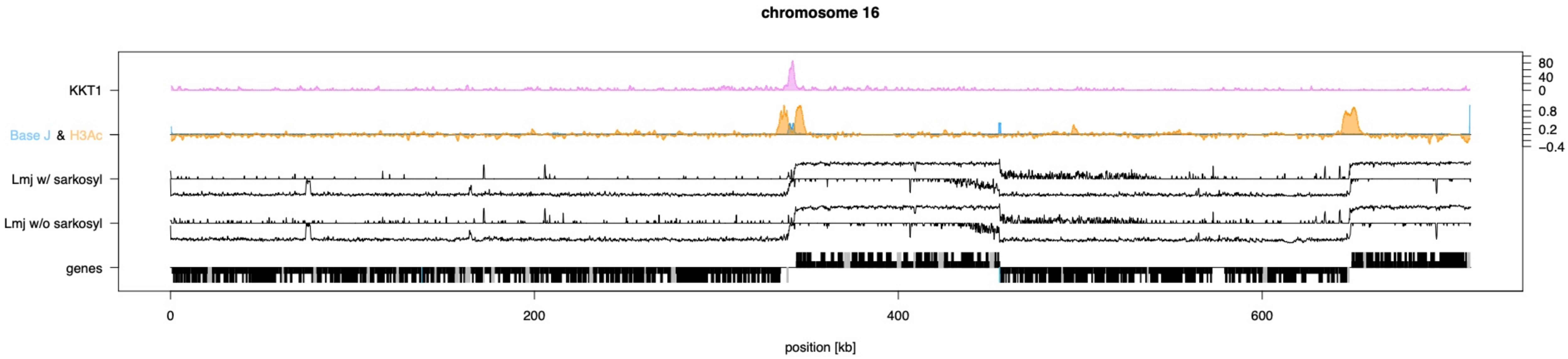

chromosome 16
KKT1
Base J & H3Ac
Lmj w/ sarkosyl
Lmj w/o sarkosyl
genes
80
40
0
0.8
0.2
−0.4
0
200
400
600
position [kb]

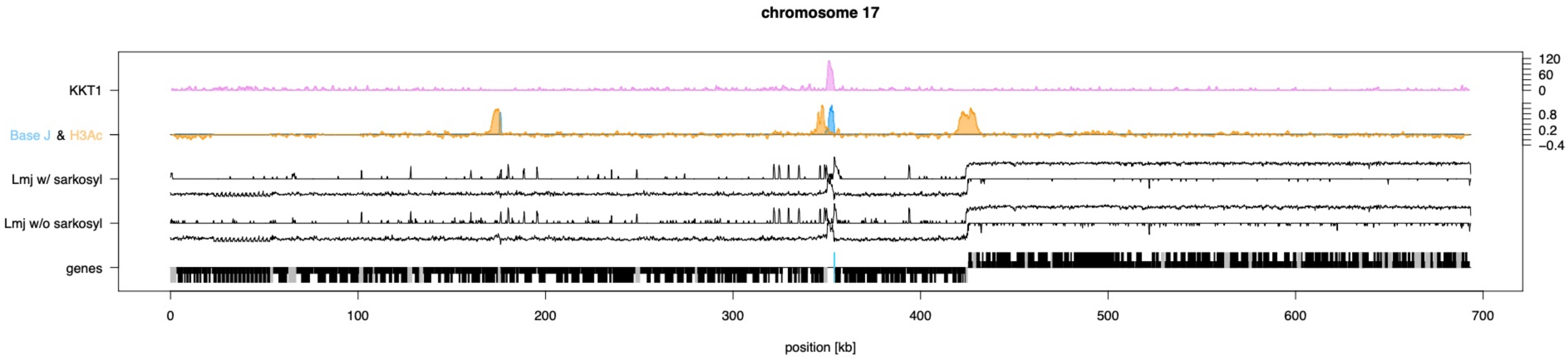

chromosome 17
KKT1
Base J & H3Ac
Lmj w/ sarkosyl
Lmj w/o sarkosyl
genes
120
60
0
0.8
0.2
−0.4
0
100
200
300
400
500
600
700
position [kb]

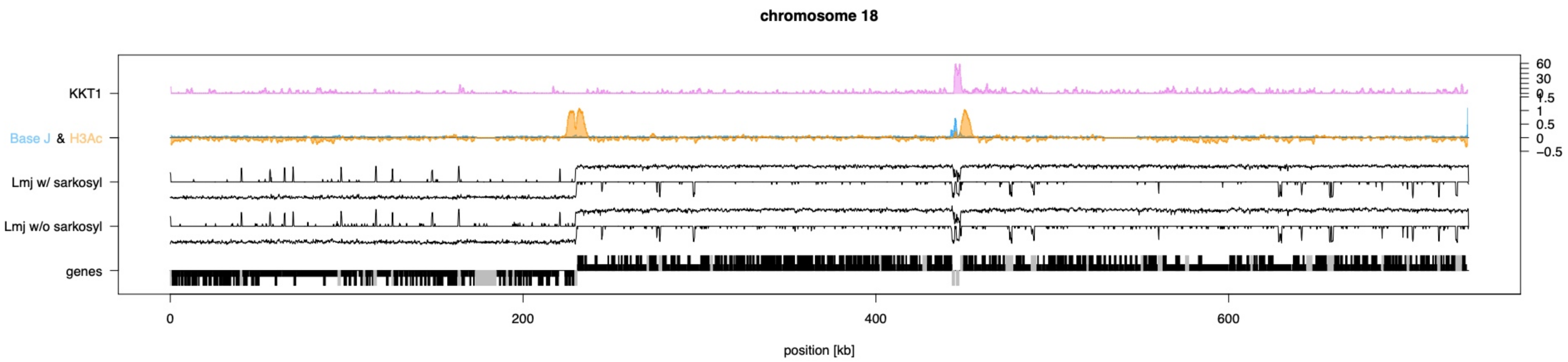
chromosome 18
KKT1
Base J & H3Ac
Lmj w/ sarkosyl
Lmj w/o sarkosyl
genes
60
30
0
1.5
1
0.5
0
−0.5
0
200
400
600
position [kb]

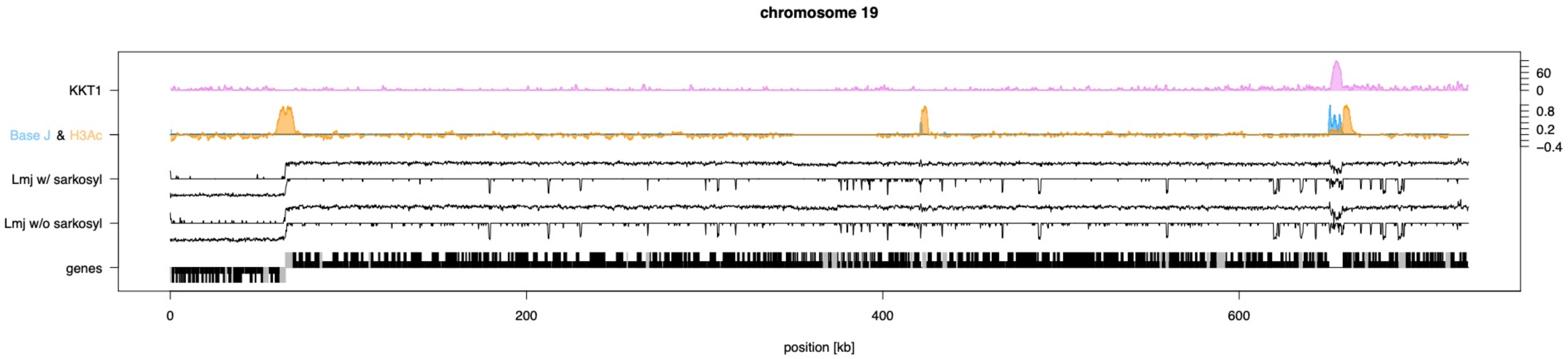
chromosome 19
KKT1
Base J & H3Ac
Lmj w/ sarkosyl
Lmj w/o sarkosyl
genes
60
0
0.8
0.2
−0.4
0
200
400
600
position [kb]

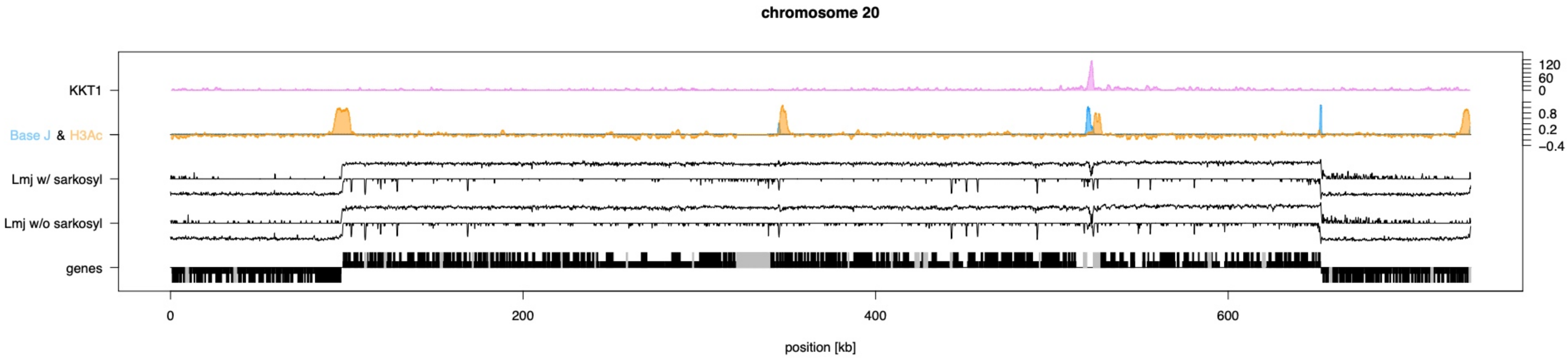
chromosome 20
KKT1
Base J & H3Ac
Lmj w/ sarkosyl
Lmj w/o sarkosyl
genes
120
60
0
0.8
0.2
−0.4
0
200
400
600
position [kb]

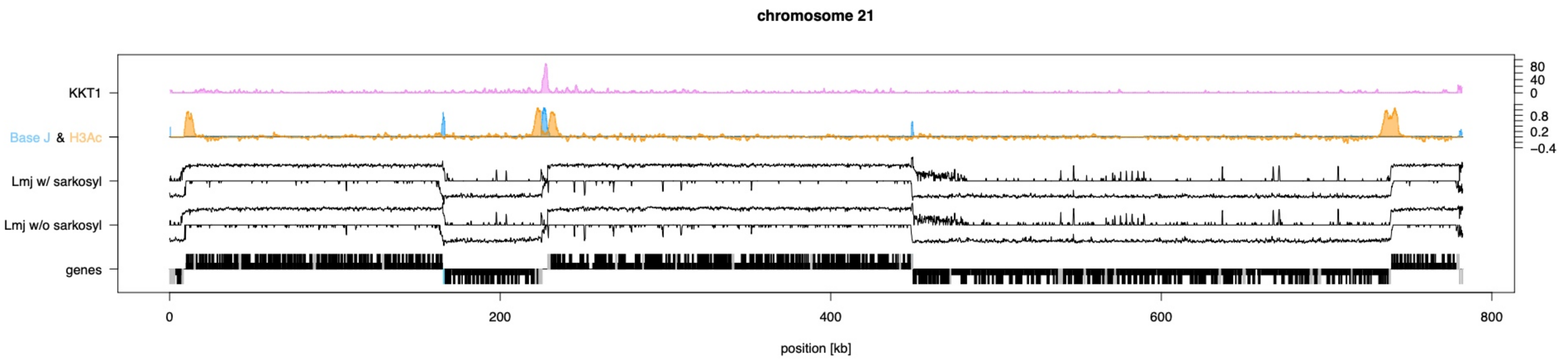

chromosome 21
KKT1
Base J & H3Ac
Lmj w/ sarkosyl
Lmj w/o sarkosyl
genes
80
40
0
0.8
0.2
−0.4
0
200
400
600
800
position [kb]

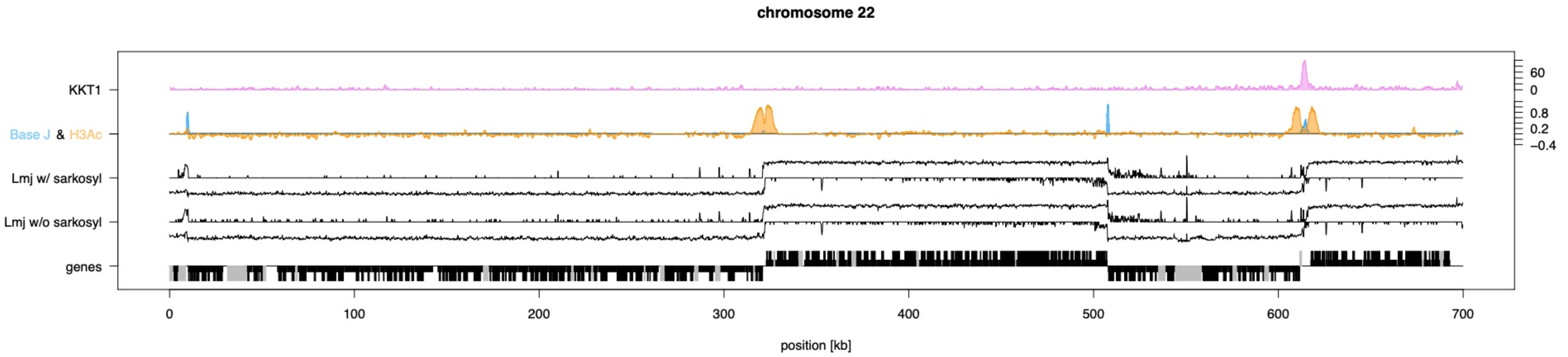

chromosome 22
KKT1
Base J & H3Ac
Lmj w/ sarkosyl
Lmj w/o sarkosyl
genes
60
0
0.8
0.2
−0.4
0
100
200
300
400
500
600
700
position [kb]

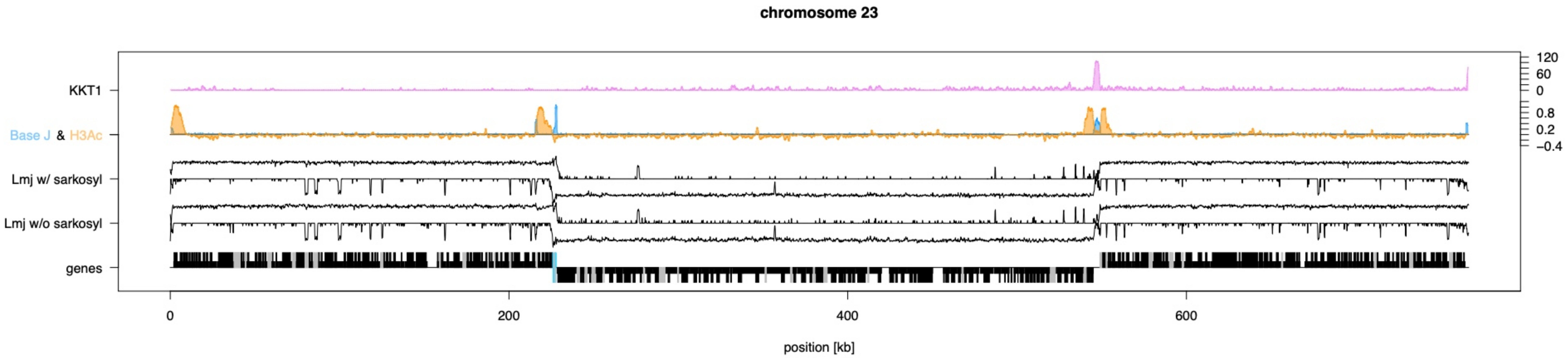

chromosome 23
KKT1
Base J & H3Ac
Lmj w/ sarkosyl
Lmj w/o sarkosyl
genes
120
60
0
0.8
0.2
−0.4
0
200
400
600
position [kb]

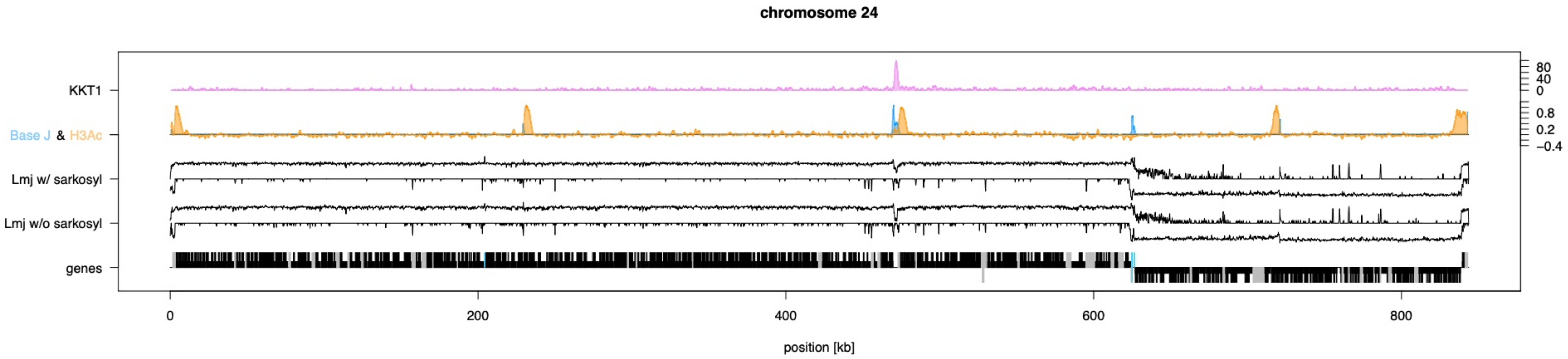

chromosome 24
KKT1
Base J & H3Ac
Lmj w/ sarkosyl
Lmj w/o sarkosyl
genes
80
40
0
0.8
0.2
−0.4
0
200
400
600
800
position [kb]

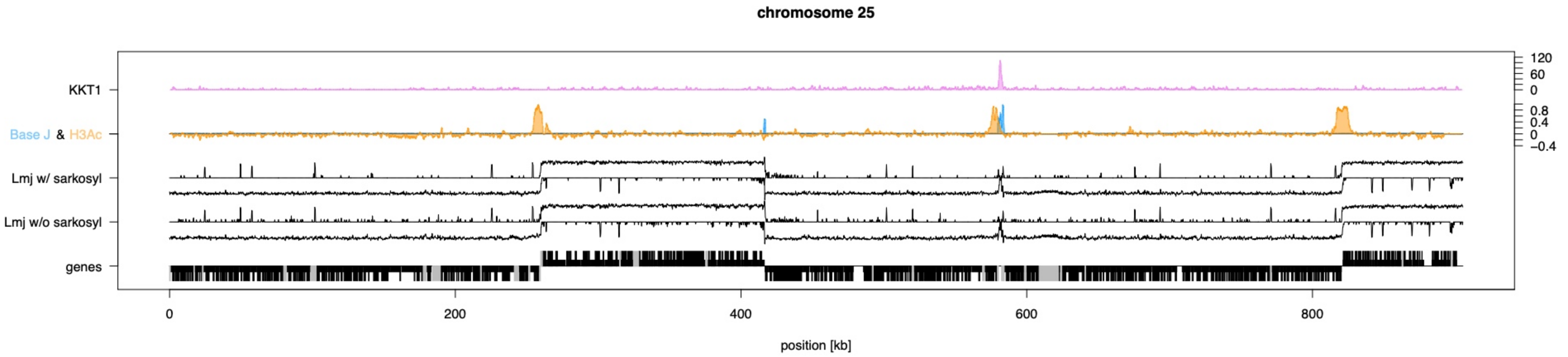
chromosome 25
KKT1
Base J & H3Ac
Lmj w/ sarkosyl
Lmj w/o sarkosyl
genes
120
60
0
0.8
0.4
0
−0.4
0
200
400
600
800
position [kb]

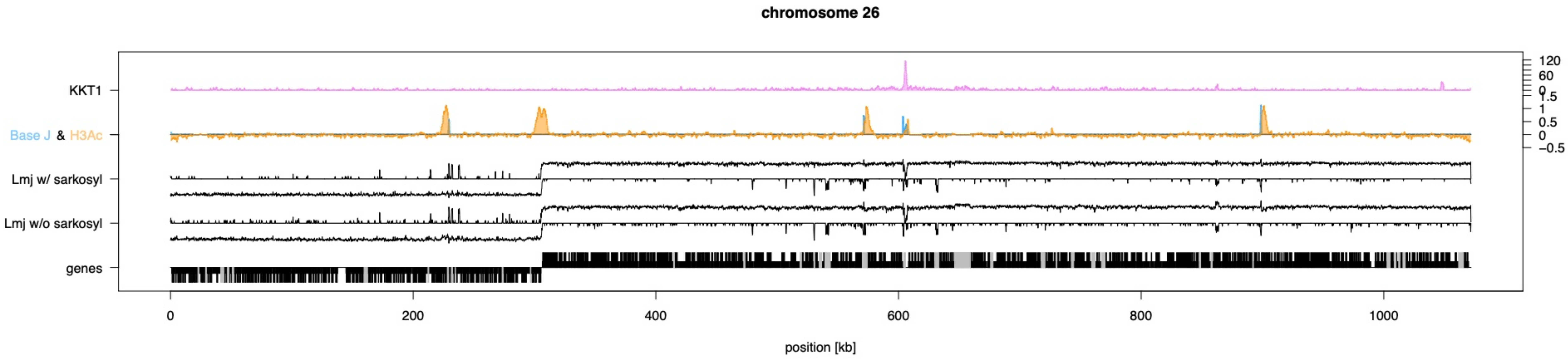
chromosome 26
KKT1
Base J & H3Ac
Lmj w/ sarkosyl
Lmj w/o sarkosyl
genes
120
60
0
1.5
1
0.5
0
−0.5
0
200
400
600
800
1000
position [kb]

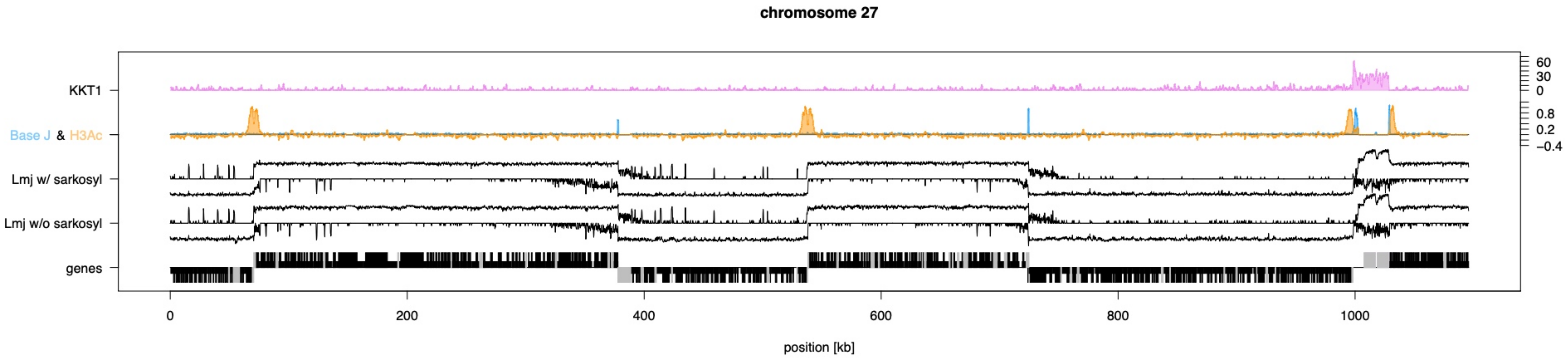

chromosome 27
KKT1
Base J & H3Ac
Lmj w/ sarkosyl
Lmj w/o sarkosyl
genes
60
30
0
0.8
0.2
−0.4
0
200
400
600
800
1000
position [kb]

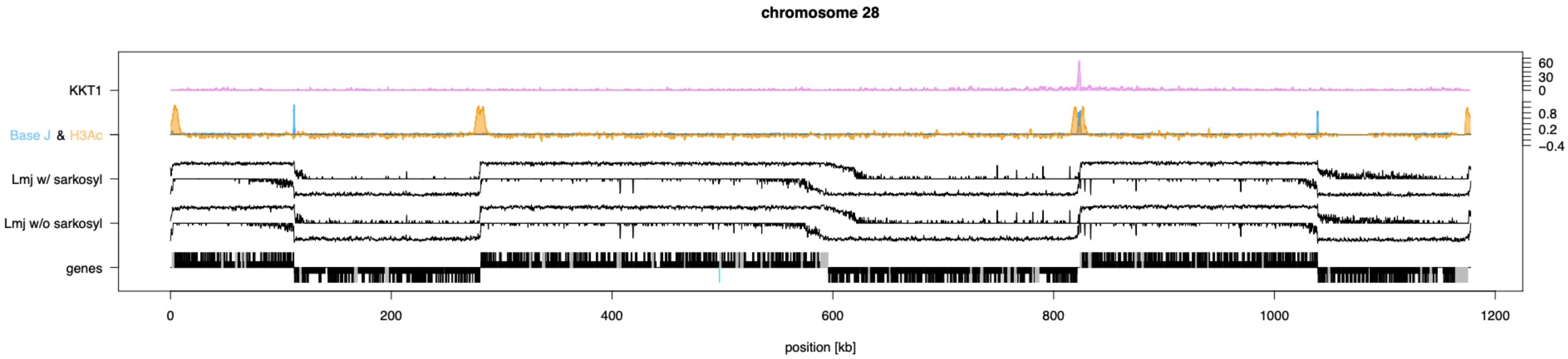

chromosome 28
KKT1
Base J & H3Ac
Lmj w/ sarkosyl
Lmj w/o sarkosyl
genes
60
30
0
0.8
0.2
−0.4
0
200
400
600
800
1000
1200
position [kb]

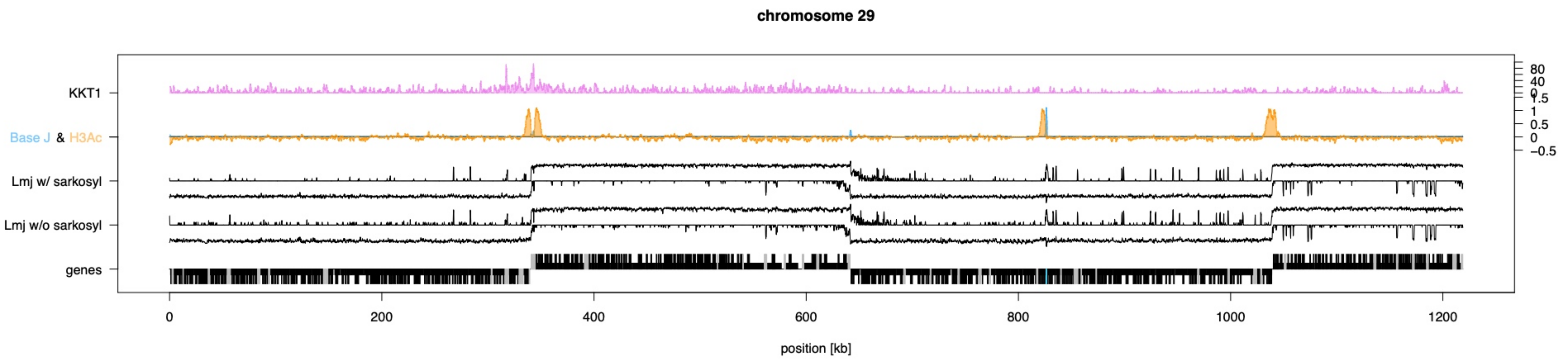
chromosome 29
KKT1
Base J & H3Ac
Lmj w/ sarkosyl
Lmj w/o sarkosyl
genes
80
40
0
1.5
1
0.5
0
−0.5
0
200
400
600
800
1000
1200
position [kb]

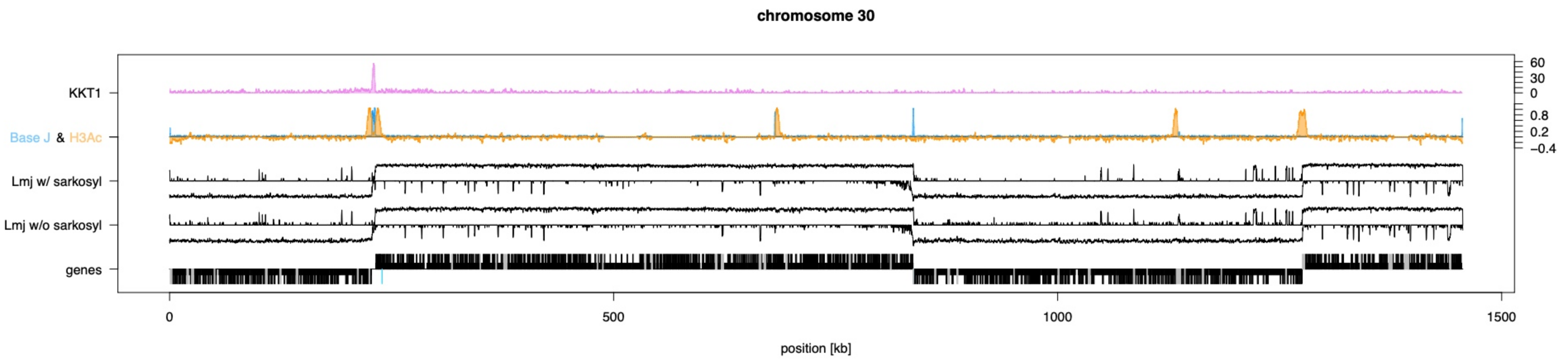

chromosome 30
KKT1
Base J & H3Ac
Lmj w/ sarkosyl
Lmj w/o sarkosyl
genes
60
30
0
0.8
0.2
−0.4
0
500
1000
1500
position [kb]

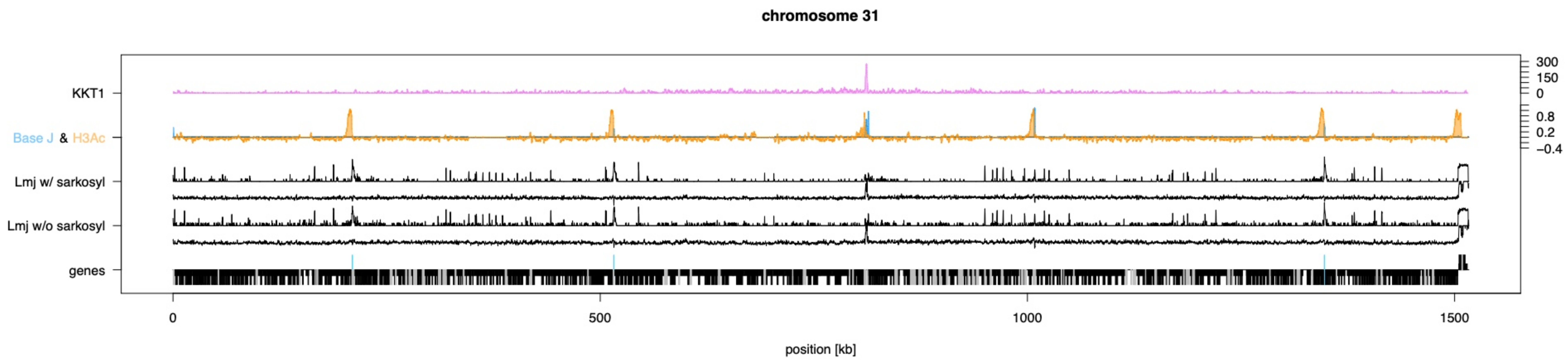
chromosome 31
KKT1
Base J & H3Ac
Lmj w/ sarkosyl
Lmj w/o sarkosyl
genes
300
150
0
0.8
0.2
−0.4
0
500
1000
1500
position [kb]

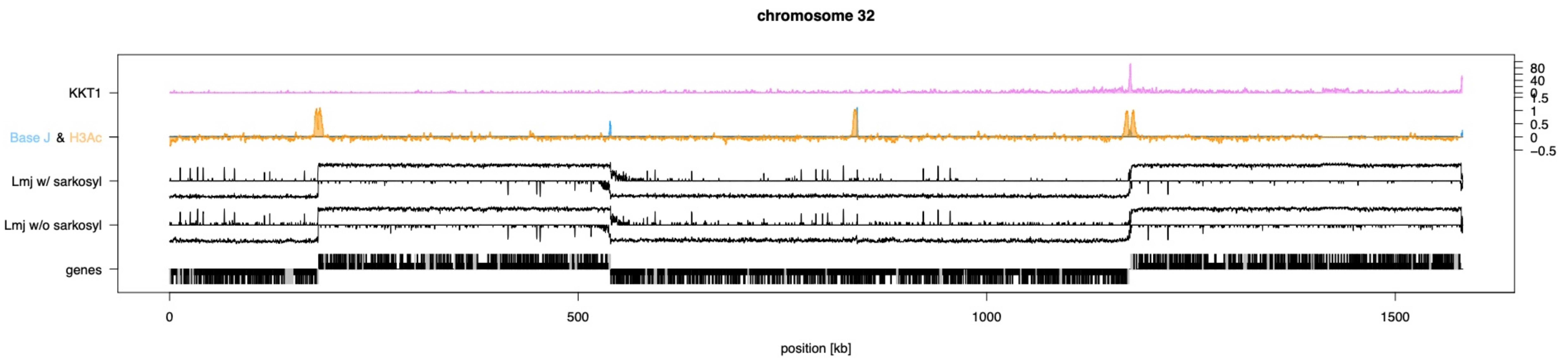

chromosome 32
KKT1
Base J & H3Ac
Lmj w/ sarkosyl
Lmj w/o sarkosyl
genes
80
40
0
1.5
1
0.5
0
−0.5
0
500
1000
1500
position [kb]

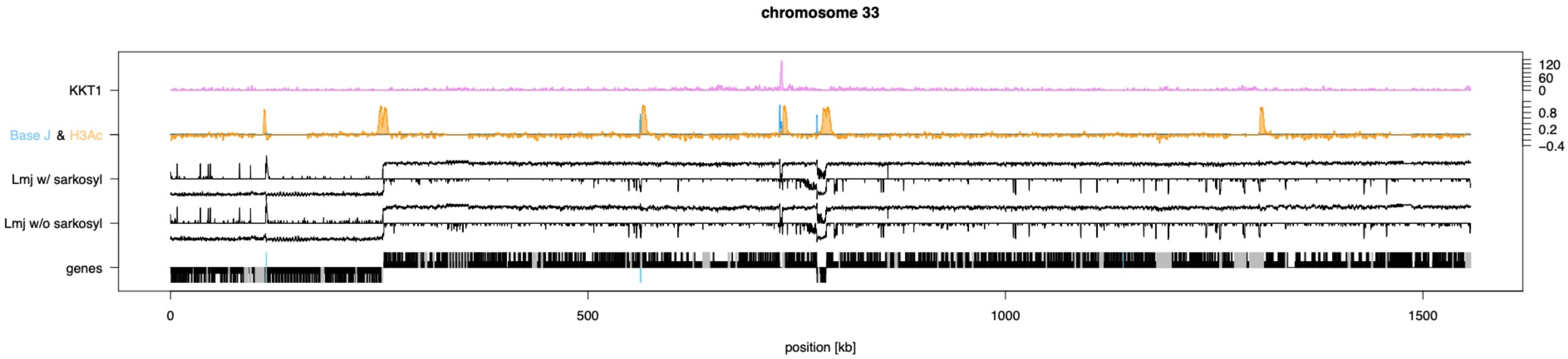
chromosome 33
KKT1
Base J & H3Ac
Lmj w/ sarkosyl
Lmj w/o sarkosyl
genes
120
60
0
0.8
0.2
−0.4
0
500
1000
1500
position [kb]

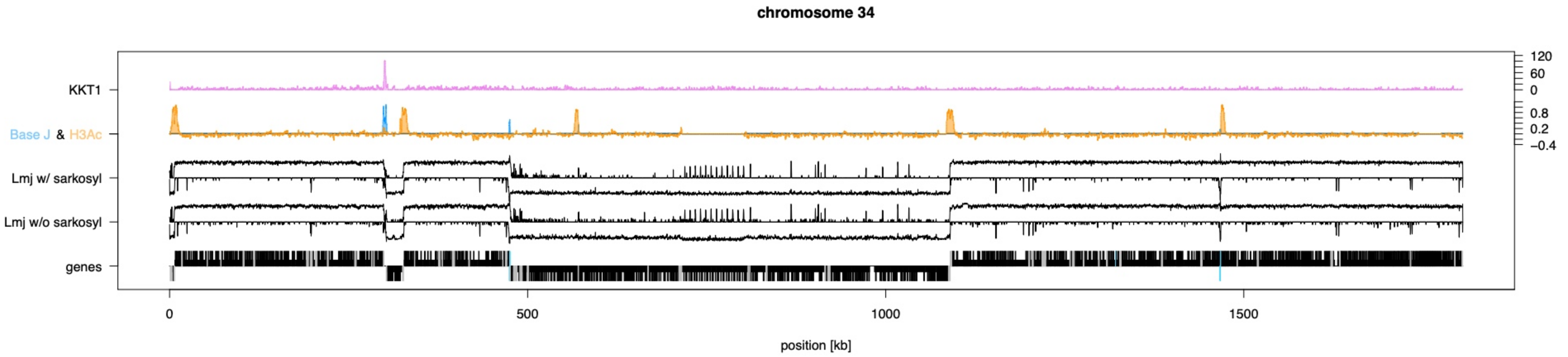
chromosome 34
KKT1
Base J & H3Ac
Lmj w/ sarkosyl
Lmj w/o sarkosyl
genes
120
60
0
0.8
0.2
−0.4
0
500
1000
1500
position [kb]

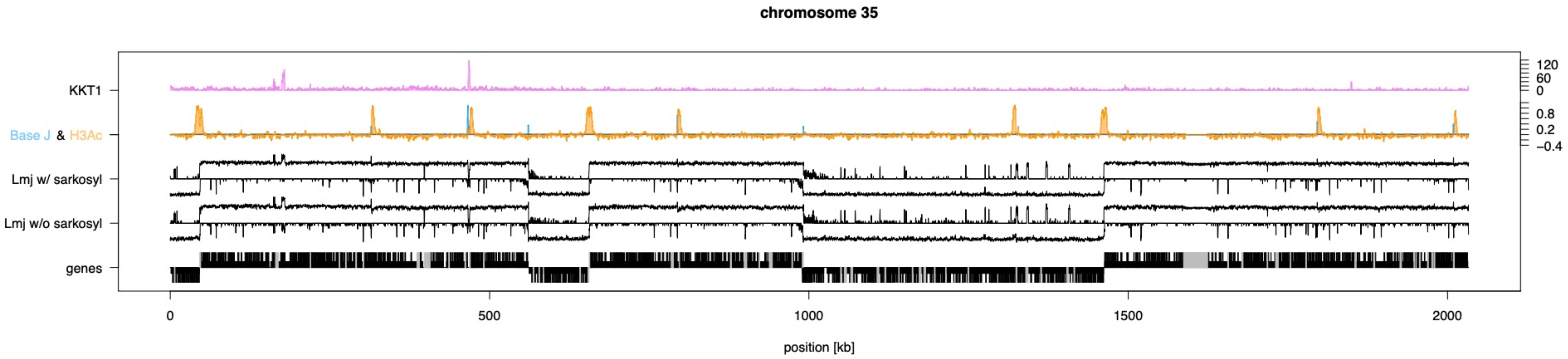

chromosome 35
KKT1
Base J & H3Ac
Lmj w/ sarkosyl
Lmj w/o sarkosyl
genes
120
60
0
0.8
0.2
-0.4
0
500
1000
1500
2000
position [kb]

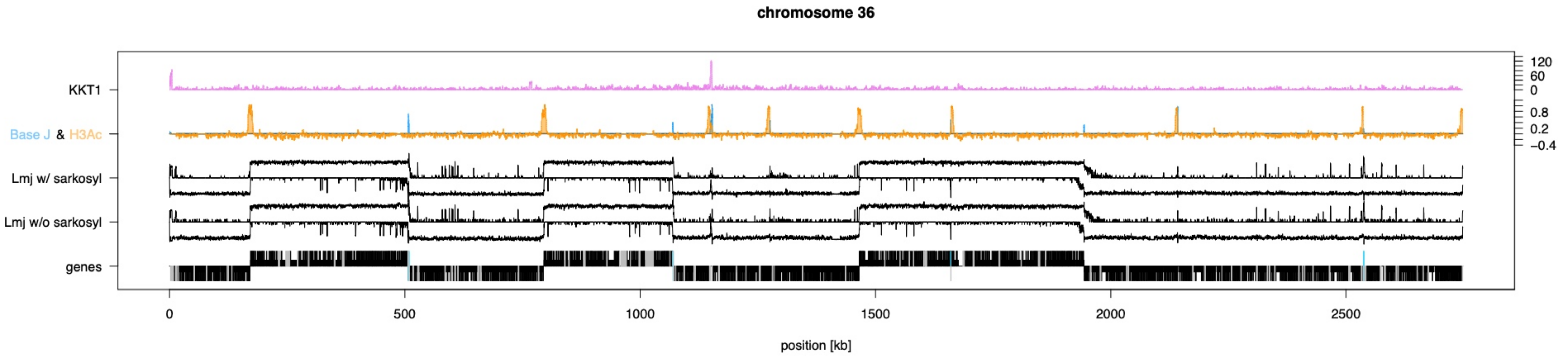
chromosome 36
KKT1
Base J & H3Ac
Lmj w/ sarkosyl
Lmj w/o sarkosyl
genes
120
60
0
0.8
0.2
−0.4
0
500
1000
1500
2000
2500
position [kb]
