# Supplementary material for: Genome-wide quantification of polycistronic transcription in Leishmania major
Source: mBio. 2024 Nov 25;16(1):e02241-24. doi: 10.1128/mbio.02241-24 (PMC11708010; doi:10.1128/mbio.02241-24)
Supplement: Figure S3 — Transcription starts at dSSRs. [file mbio.02241-24-s0002.pdf]

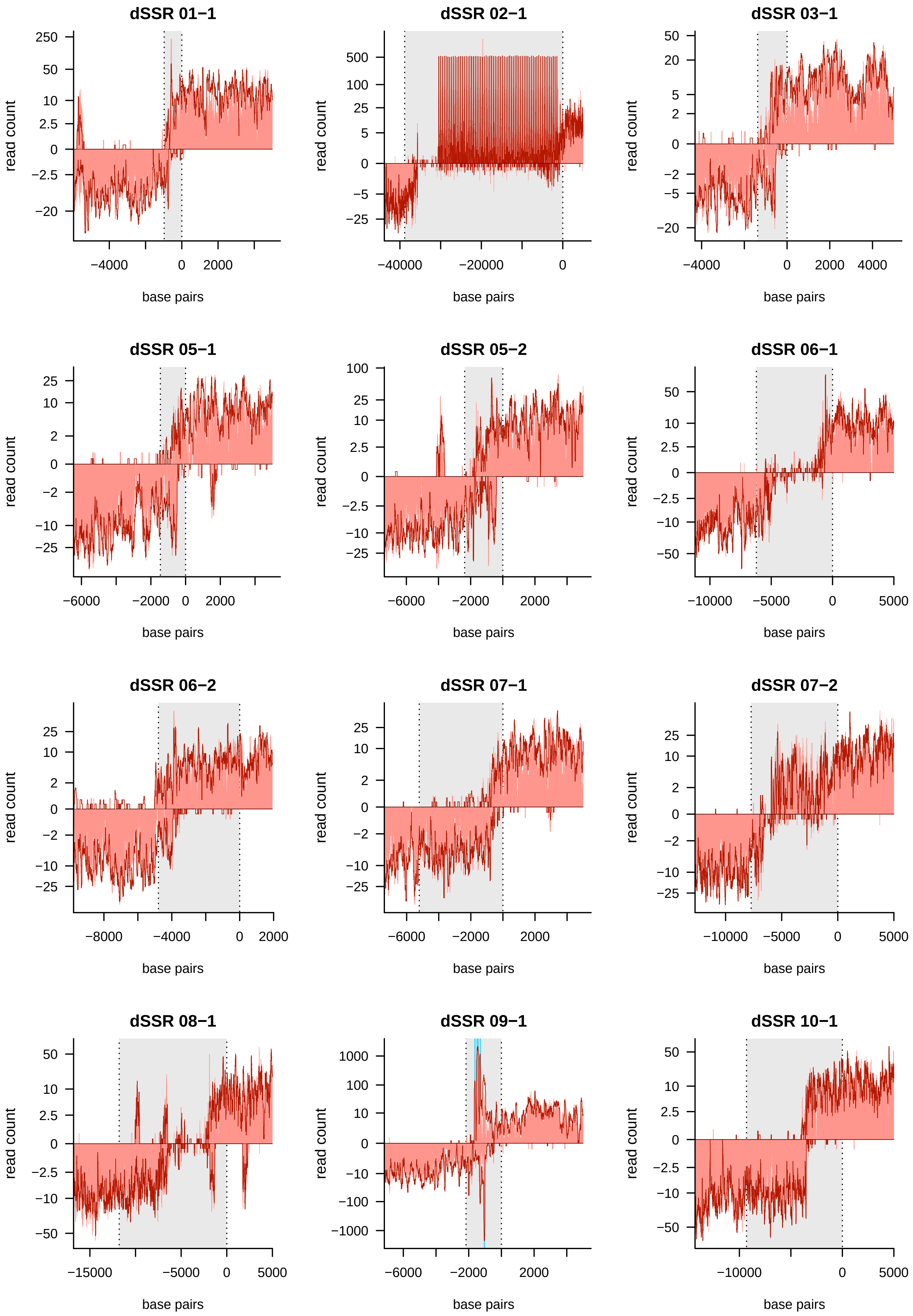

dSSR 01−1
read count
250
50
10
2.5
0
−2.5
−20
−4000
0
2000
base pairs
dSSR 02−1
500
100
25
5
0
−5
−25
−40000
−20000
dSSR 03−1
20
2
−2
−5
4000
dSSR 05−1
−6000
−2000
dSSR 05−2
dSSR 06−1
−10
−50
−10000
−5000
5000
dSSR 06−2
−8000
dSSR 07−1
dSSR 07−2
dSSR 08−1
−15000
dSSR 09−1
1000
−100
−1000
dSSR 10−1

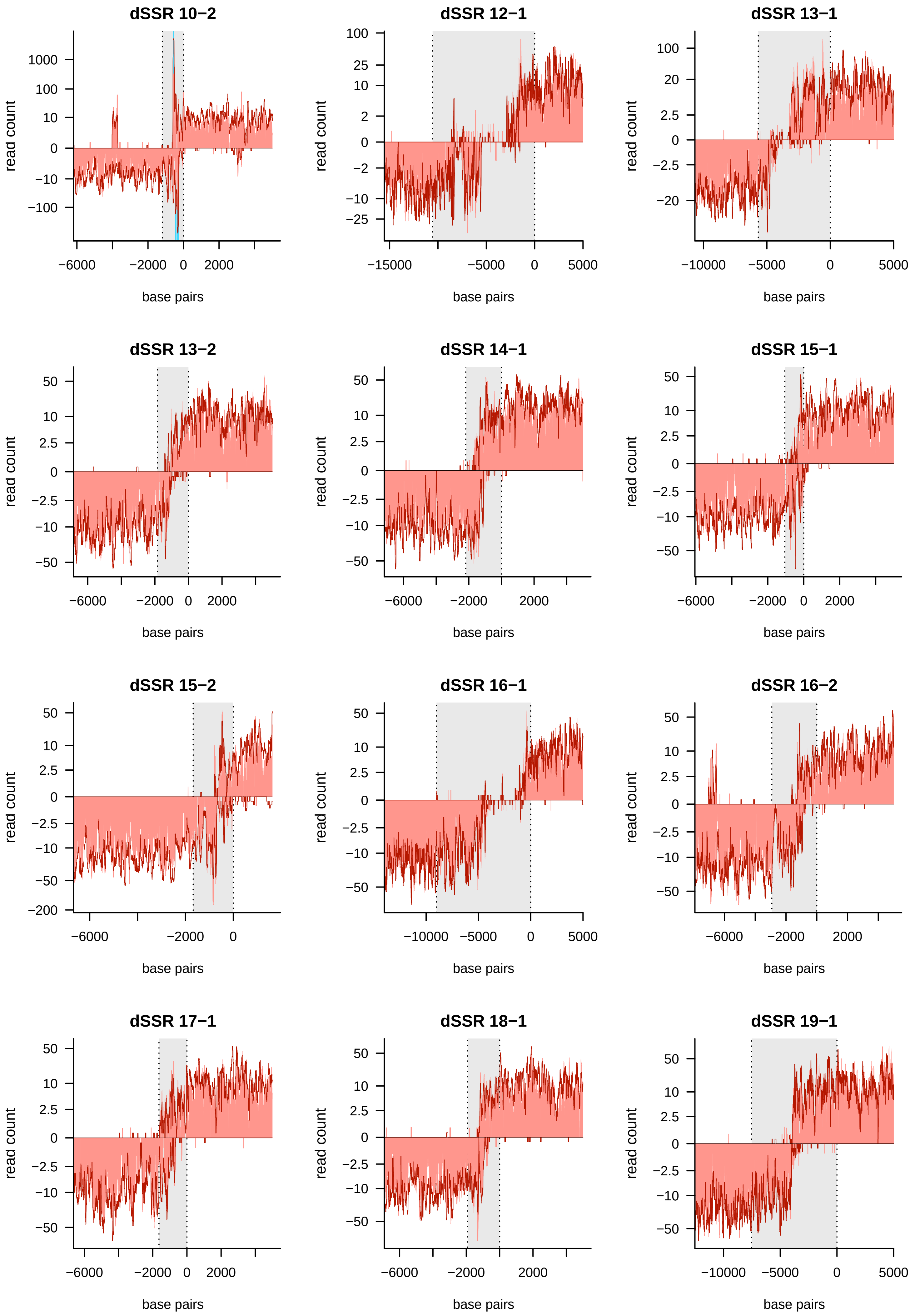
dSSR 10−2
read count
1000
100
10
0
−10
−100
−6000
−2000
0
2000
base pairs
dSSR 12−1
read count
100
25
10
2
0
−2
−10
−25
−15000
−5000
0
5000
base pairs
dSSR 13−1
read count
100
20
2.5
0
−2.5
−20
−10000
−5000
0
5000
base pairs
dSSR 13−2
read count
50
10
2.5
0
−2.5
−10
−50
−6000
−2000
0
2000
base pairs
dSSR 14−1
read count
50
10
2.5
0
−2.5
−10
−50
−6000
−2000
2000
base pairs
dSSR 15−1
read count
50
10
2.5
0
−2.5
−10
−50
−6000
−2000
0
2000
base pairs
dSSR 15−2
read count
50
10
2.5
0
−2.5
−10
−50
−200
−6000
−2000
0
base pairs
dSSR 16−1
read count
50
10
2.5
0
−2.5
−10
−50
−10000
−5000
0
5000
base pairs
dSSR 16−2
read count
50
10
2.5
0
−2.5
−10
−50
−6000
−2000
2000
base pairs
dSSR 17−1
read count
50
10
2.5
0
−2.5
−10
−50
−6000
−2000
0
2000
base pairs
dSSR 18−1
read count
50
10
2.5
0
−2.5
−10
−50
−6000
−2000
2000
base pairs
dSSR 19−1
read count
50
10
2.5
0
−2.5
−10
−50
−10000
−5000
0
5000
base pairs

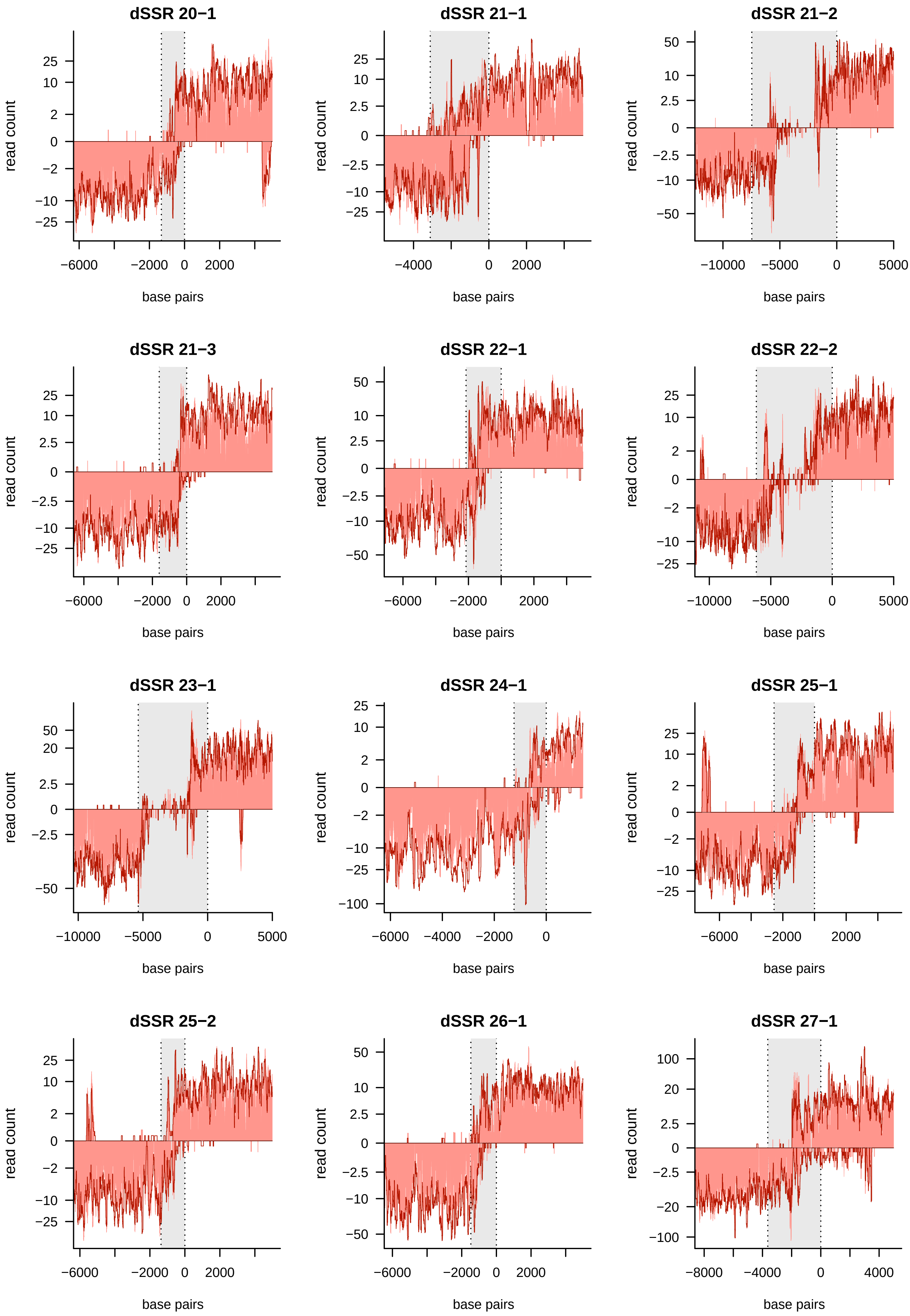
dSSR 20−1
dSSR 21−1
dSSR 21−2
dSSR 21−3
dSSR 22−1
dSSR 22−2
dSSR 23−1
dSSR 24−1
dSSR 25−1
dSSR 25−2
dSSR 26−1
dSSR 27−1
read count
base pairs

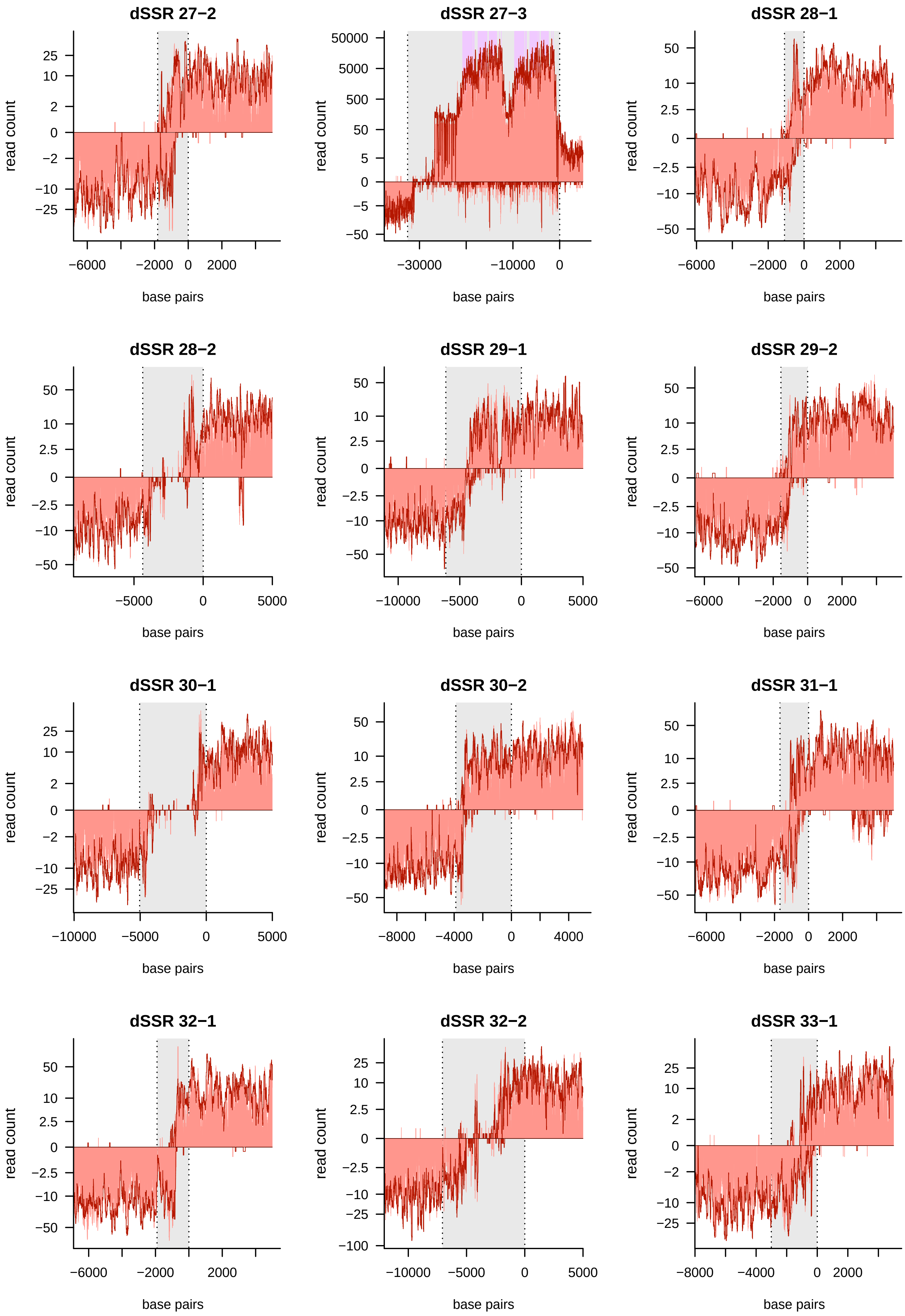
dSSR 27−2
read count
25
10
2
0
−2
−10
−25
−6000
−2000
0
2000
base pairs
dSSR 27−3
50000
5000
500
50
5
0
−5
−50
−30000
−10000
0
dSSR 28−1
50
10
2.5
0
−2.5
−10
−50
dSSR 28−2
−5000
5000
dSSR 29−1
−10000
dSSR 29−2
dSSR 30−1
dSSR 30−2
−8000
−4000
4000
dSSR 31−1
dSSR 32−1
dSSR 32−2
−25
−100
dSSR 33−1

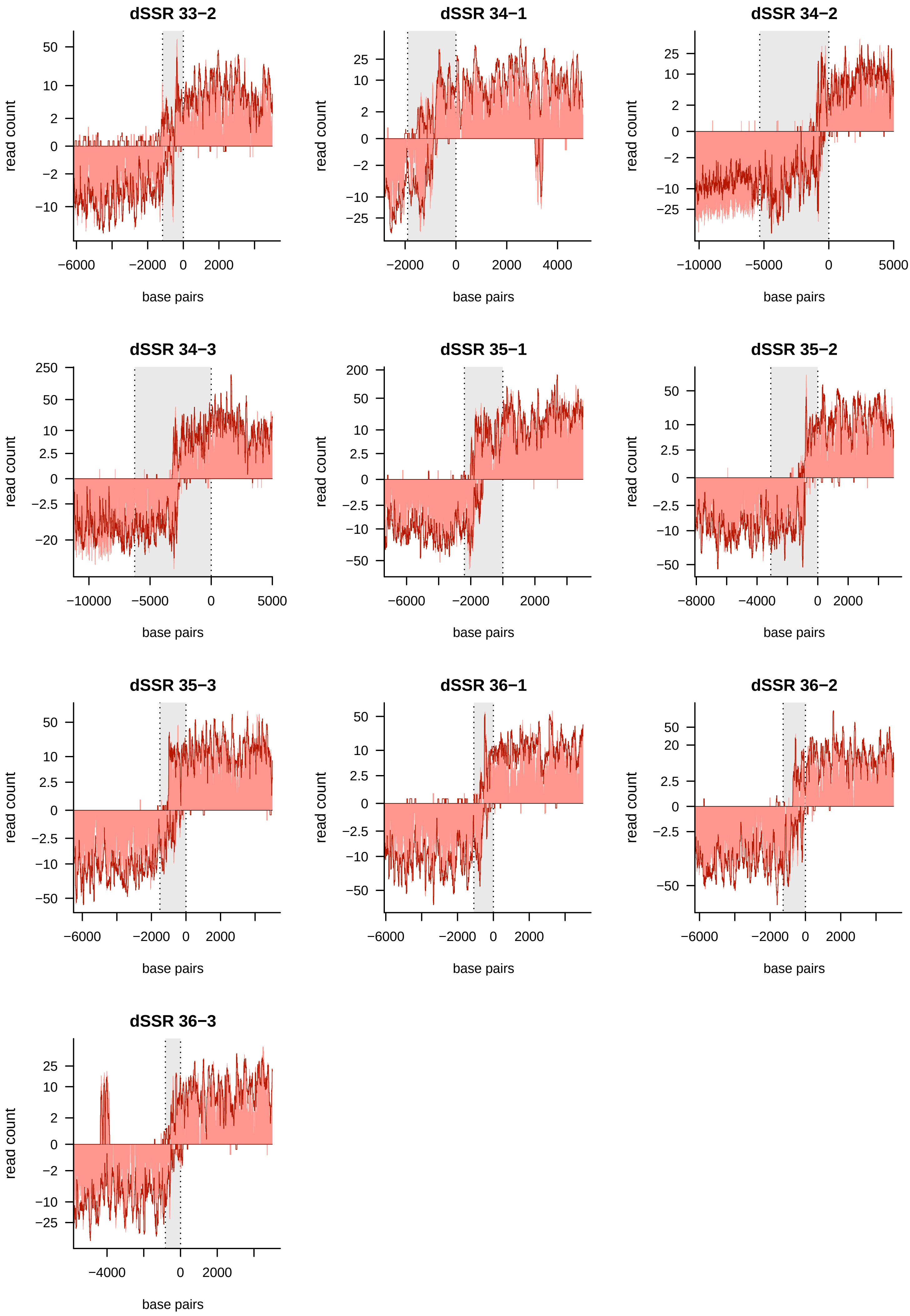
dSSR 33−2
50
10
2
0
−2
−10
read count
−6000
−2000
0
2000
base pairs
dSSR 34−1
25
10
2
0
−2
−10
−25
read count
−2000
0
2000
4000
base pairs
dSSR 34−2
25
10
2
0
−2
−10
−25
read count
−10000
−5000
0
5000
base pairs
dSSR 34−3
250
50
10
2.5
0
−2.5
−20
read count
−10000
−5000
0
5000
base pairs
dSSR 35−1
200
50
10
2.5
0
−2.5
−10
−50
read count
−6000
−2000
2000
base pairs
dSSR 35−2
50
10
2.5
0
−2.5
−10
−50
read count
−8000
−4000
0
2000
base pairs
dSSR 35−3
50
10
2.5
0
−2.5
−10
−50
read count
−6000
−2000
0
2000
base pairs
dSSR 36−1
50
10
2.5
0
−2.5
−10
−50
read count
−6000
−2000
0
2000
base pairs
dSSR 36−2
50
20
2.5
0
−2.5
−50
read count
−6000
−2000
0
2000
base pairs
dSSR 36−3
25
10
2
0
−2
−10
−25
read count
−4000
0
2000
base pairs
