# Supplementary figures and images for: Genome-wide quantification of polycistronic transcription in Leishmania major
Source: mBio. 2024 Nov 25;16(1):e02241-24. doi: 10.1128/mbio.02241-24 (PMC11708010; doi:10.1128/mbio.02241-24)

**cSSR 03-1**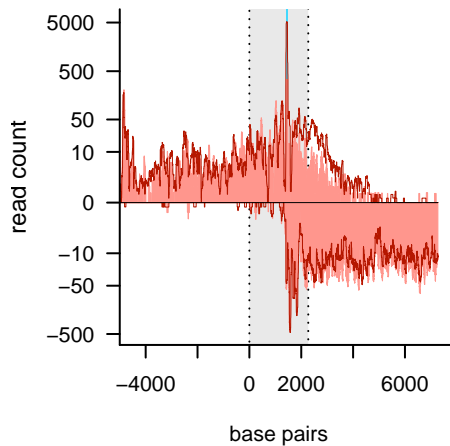**cSSR 04-1**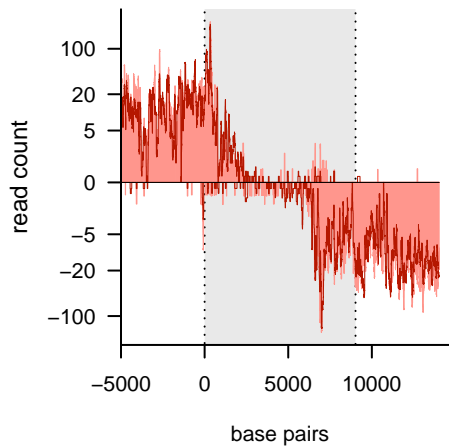**cSSR 05-1**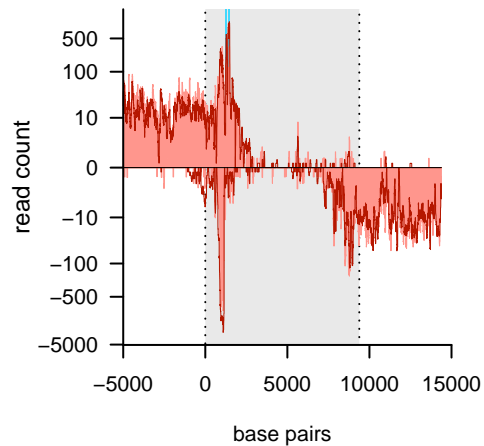**cSSR 05-2**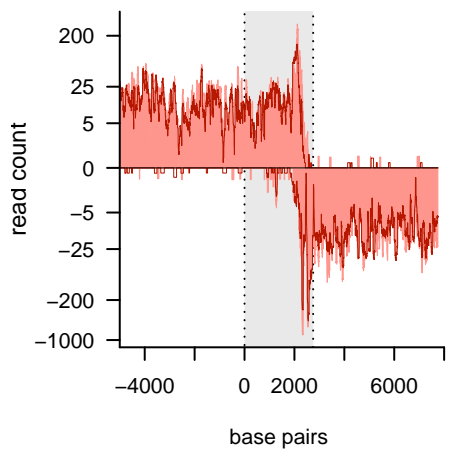**cSSR 06-1**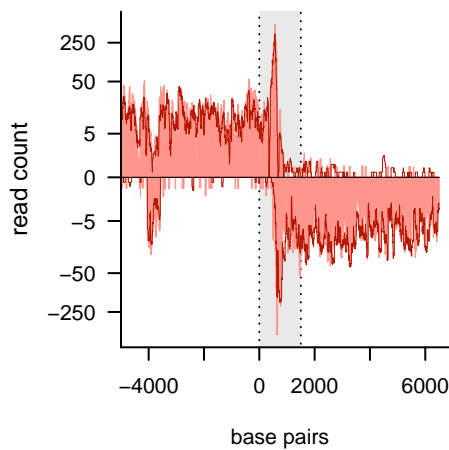**cSSR 07-1**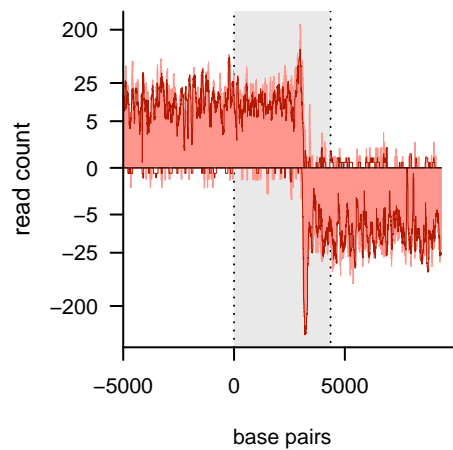**cSSR 08-1**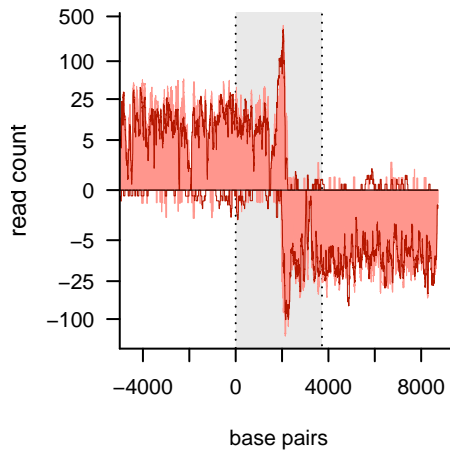**cSSR 09-1**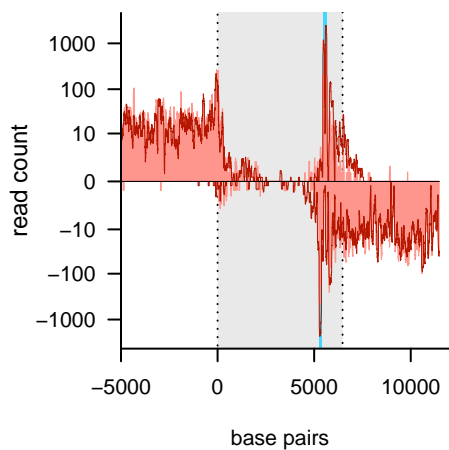**cSSR 09-2**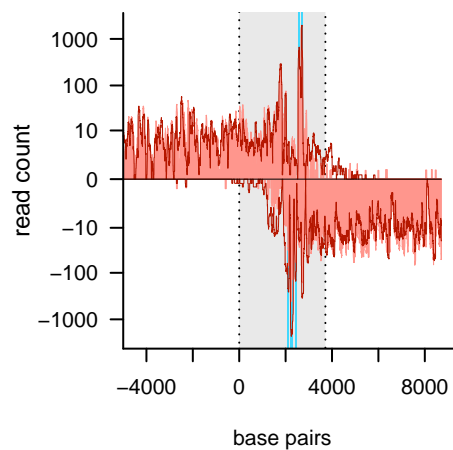**cSSR 10-1**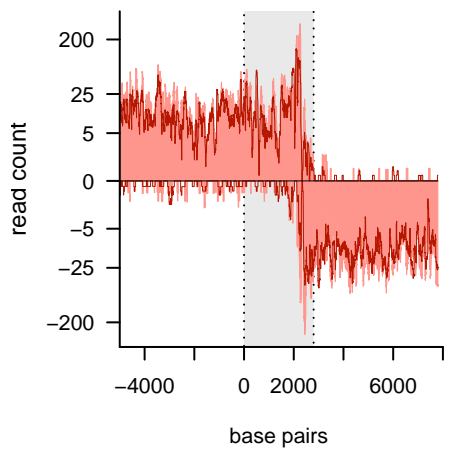**cSSR 12-1**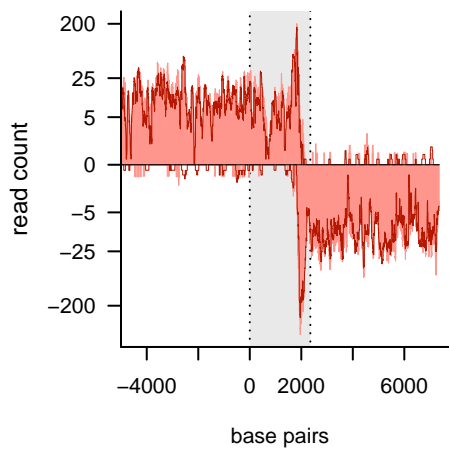**cSSR 13-1**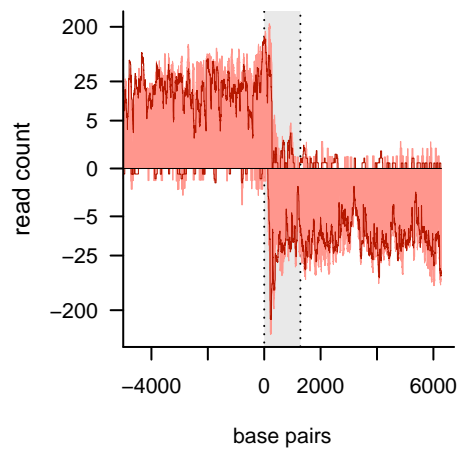

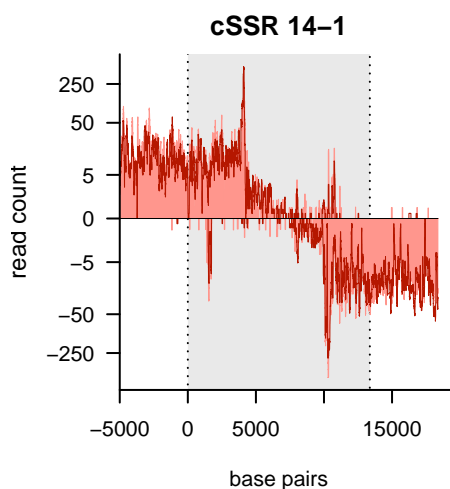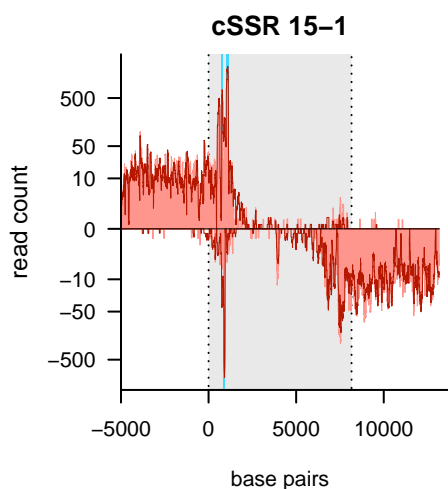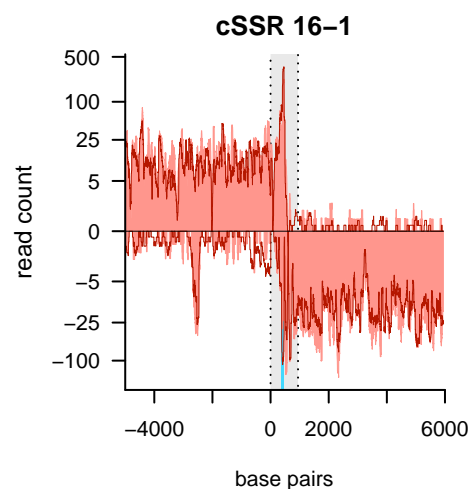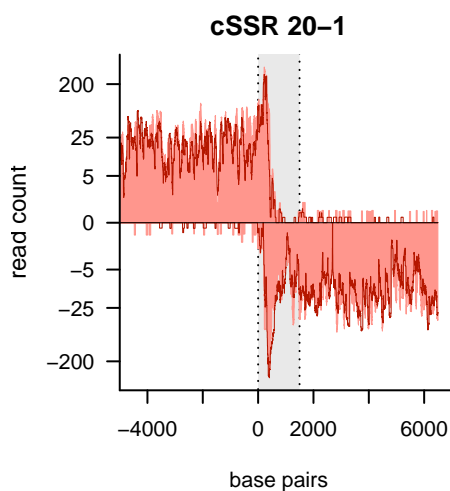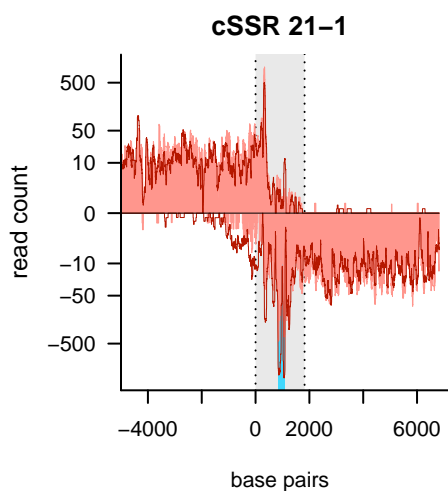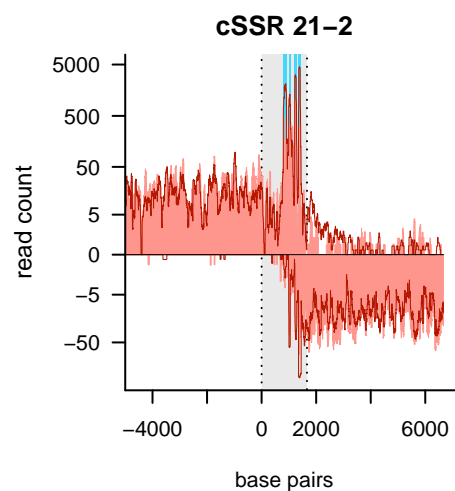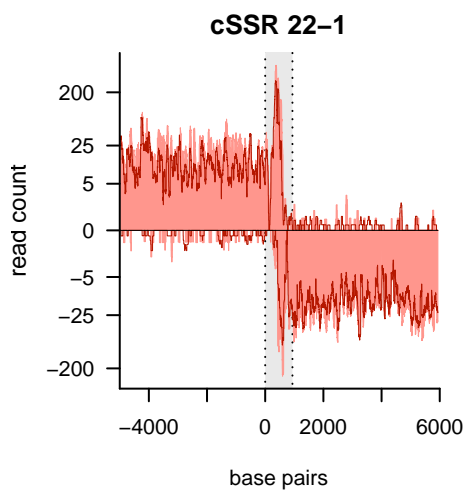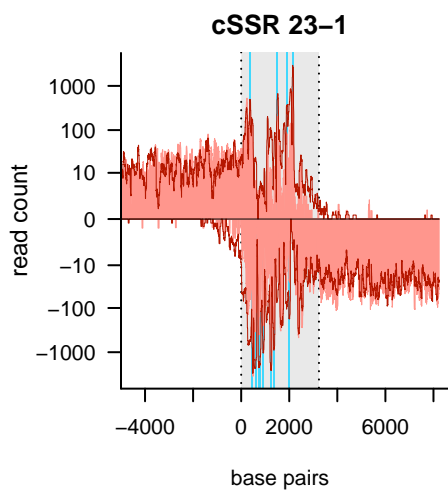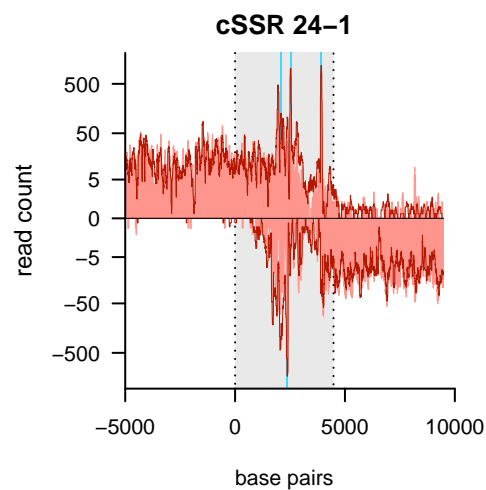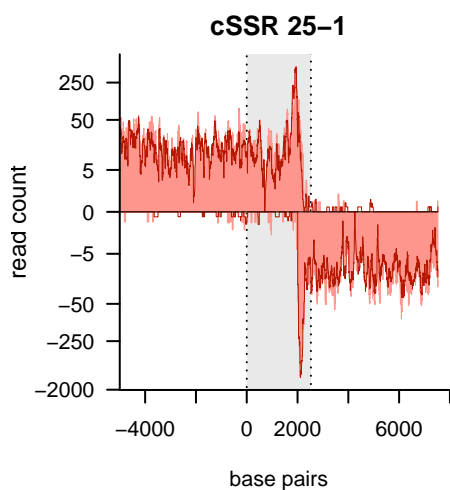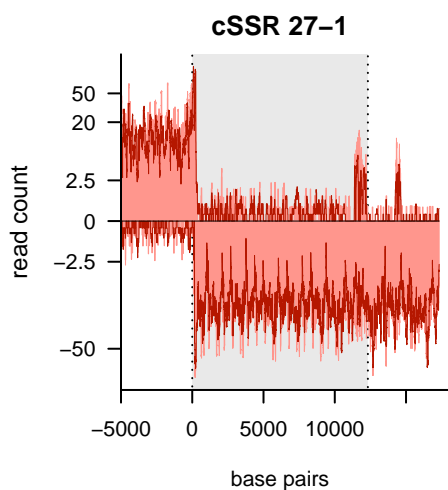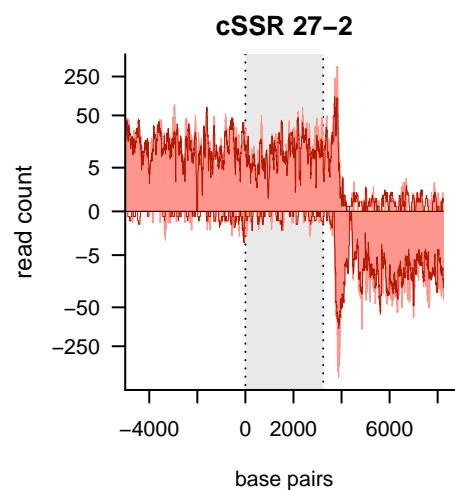

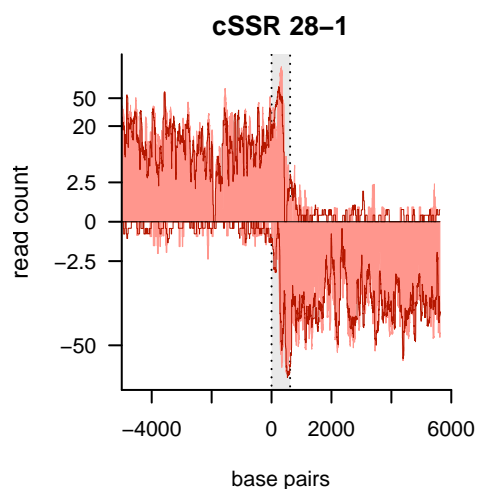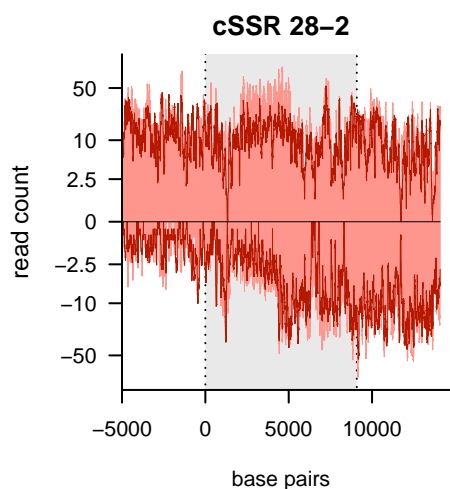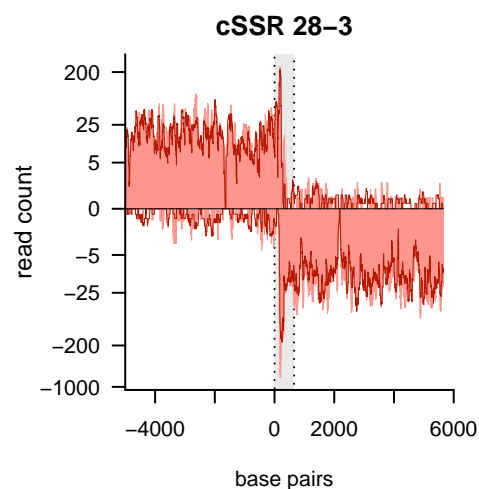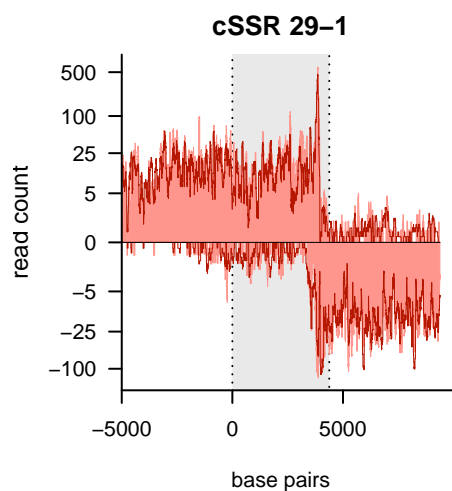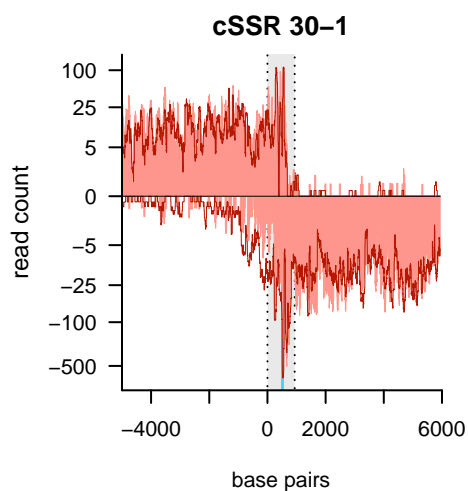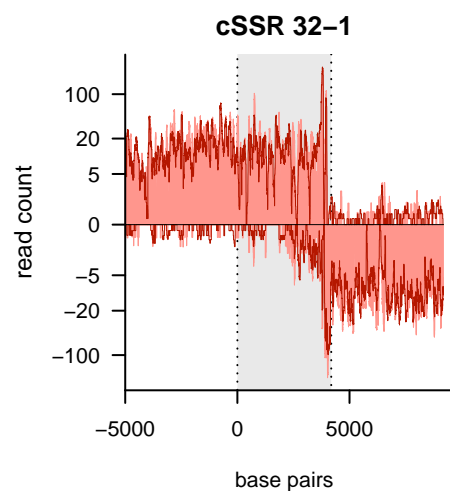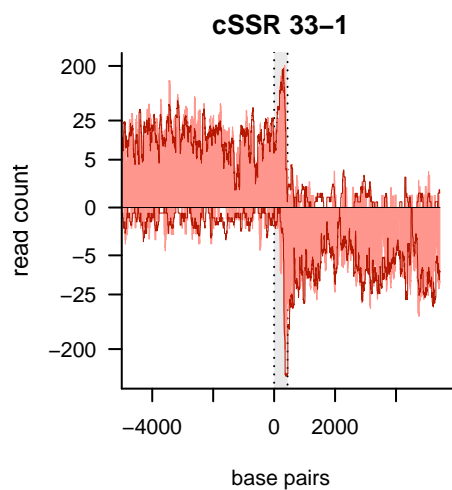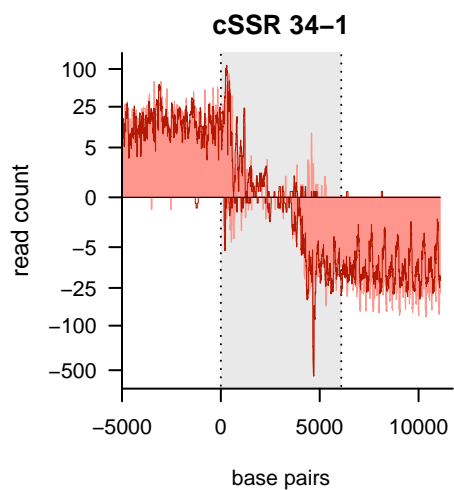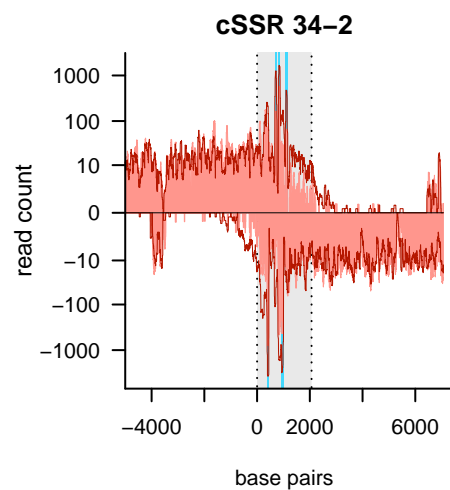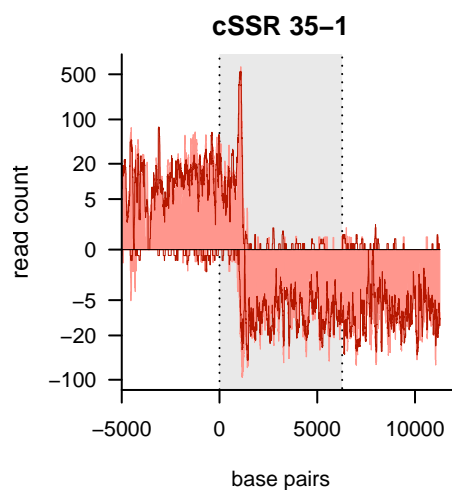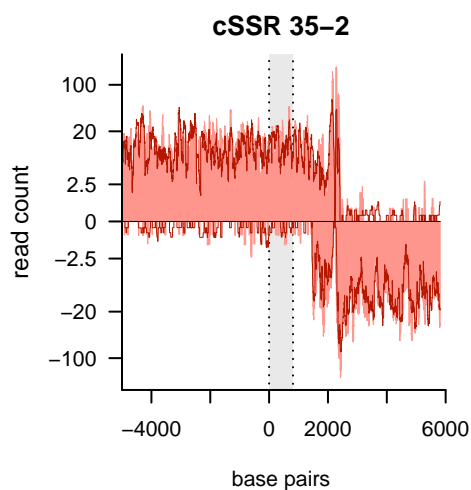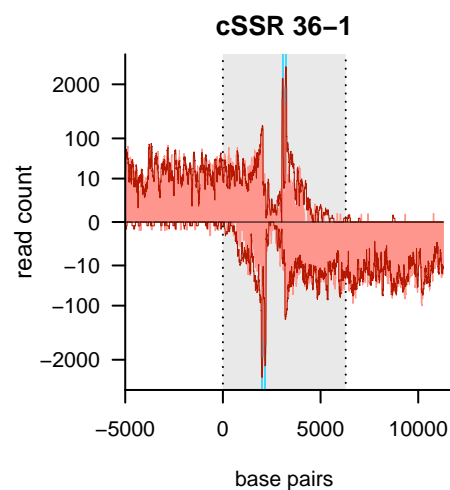

**cSSR 36-2**

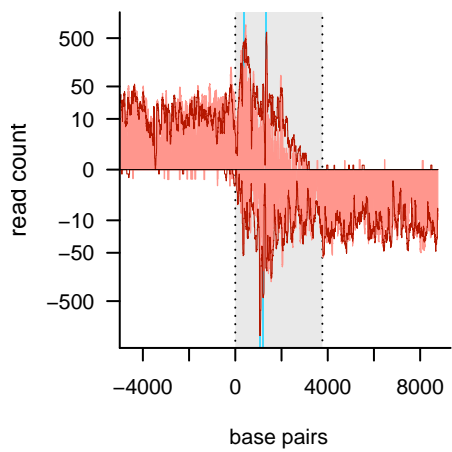

**cSSR 36-3**

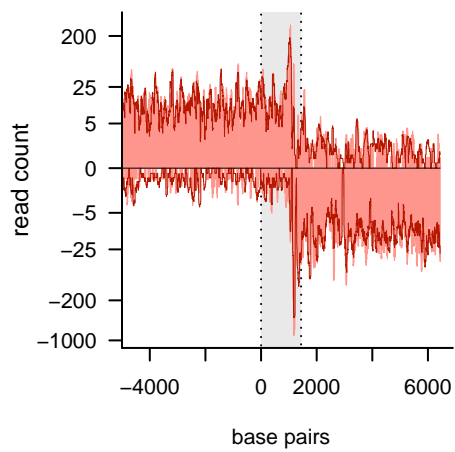

Supplement: Figure S4 — Transcription stops at cSSRs. [file mbio.02241-24-s0003.pdf]

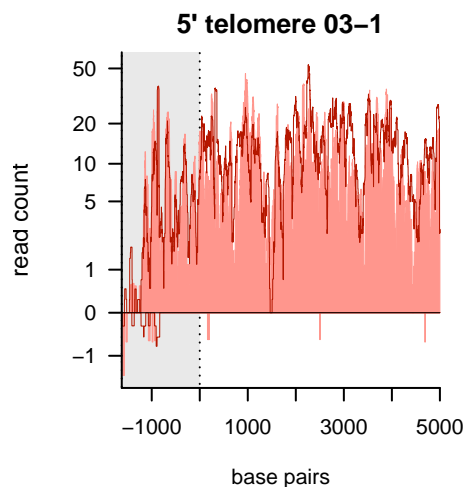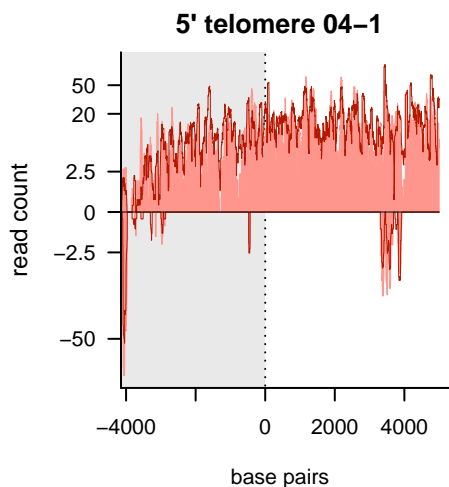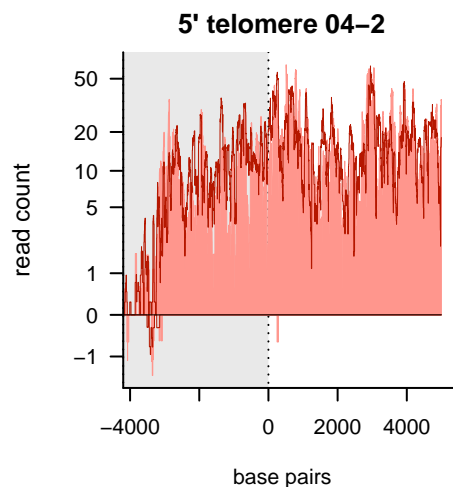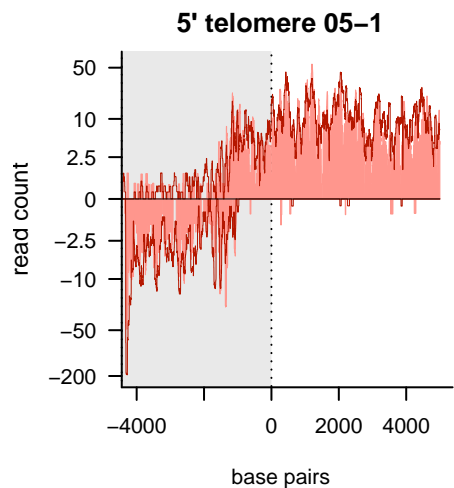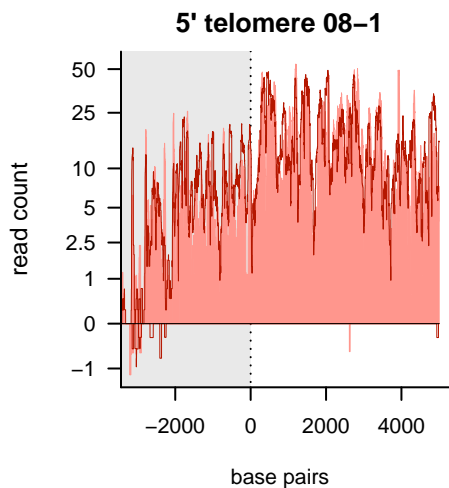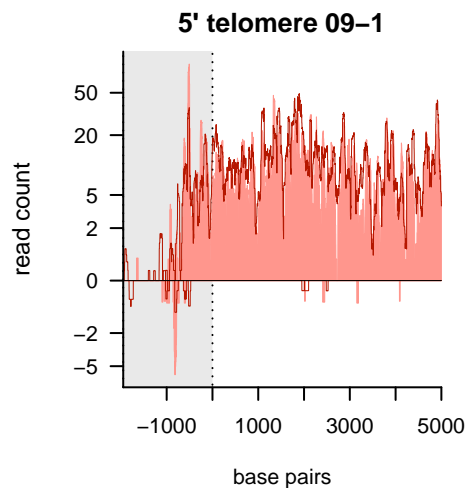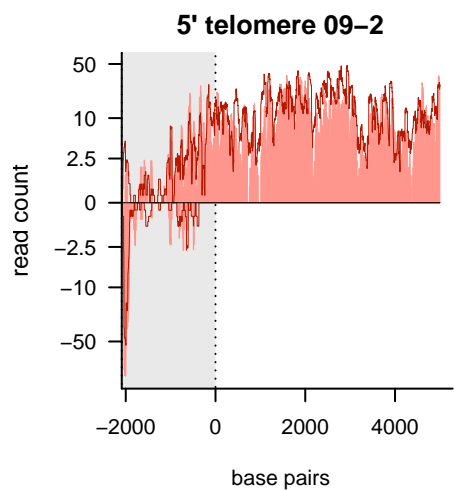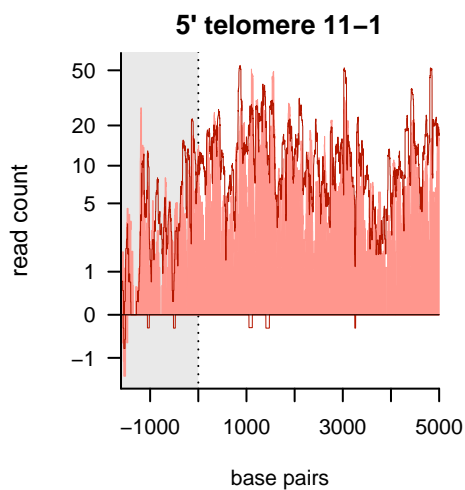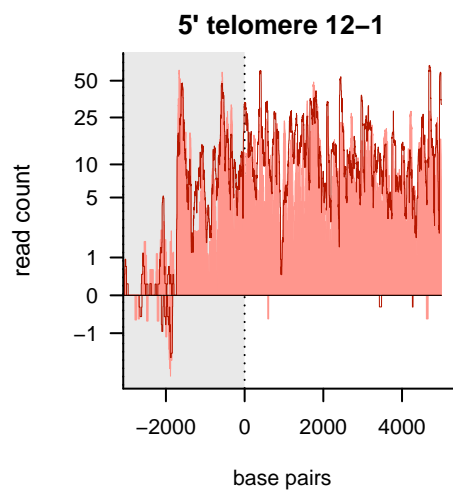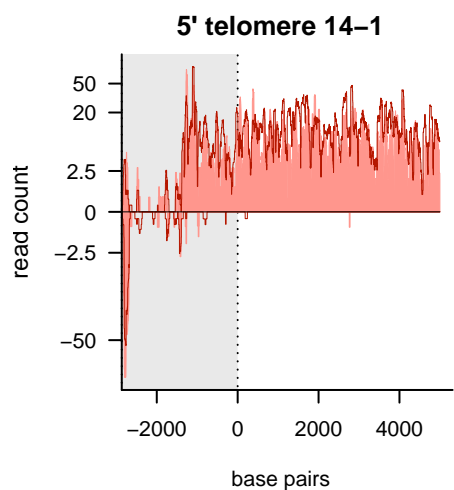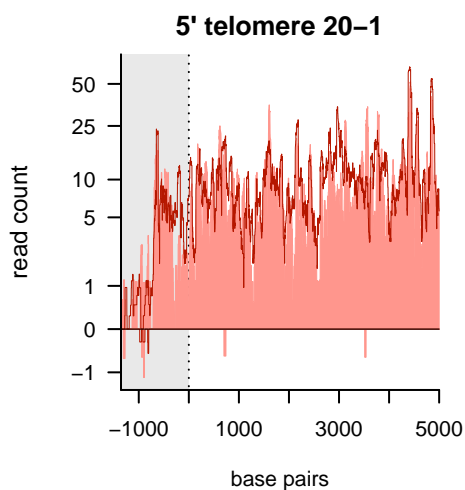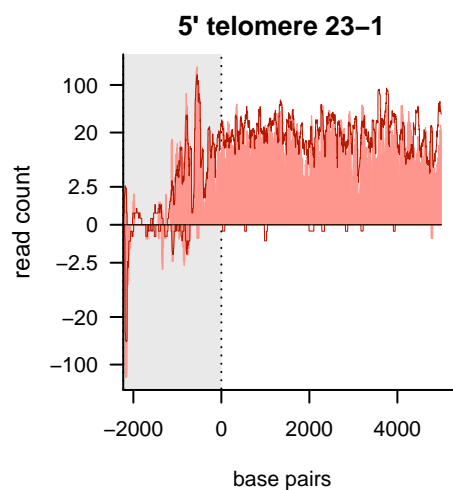

**5' telomere 24-1**

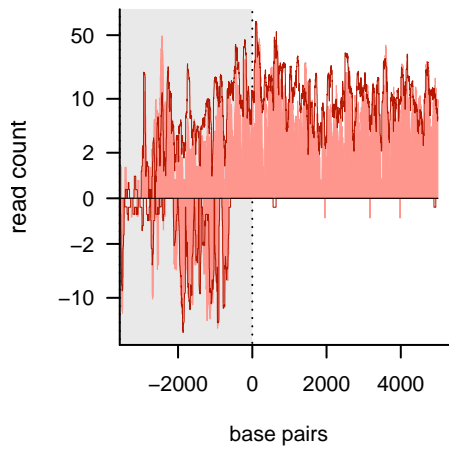

**5' telomere 28-1**

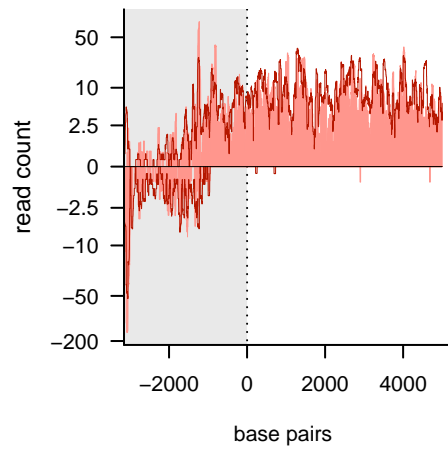

**5' telomere 28-2**

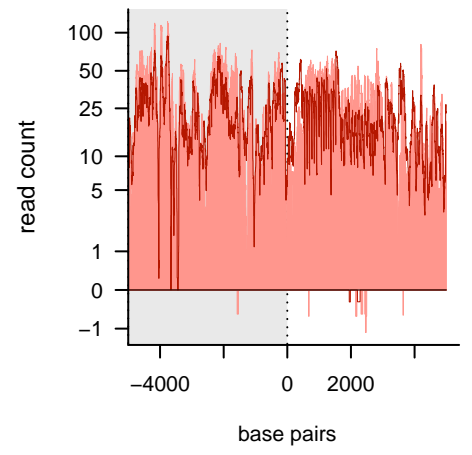

**5' telomere 36-1**

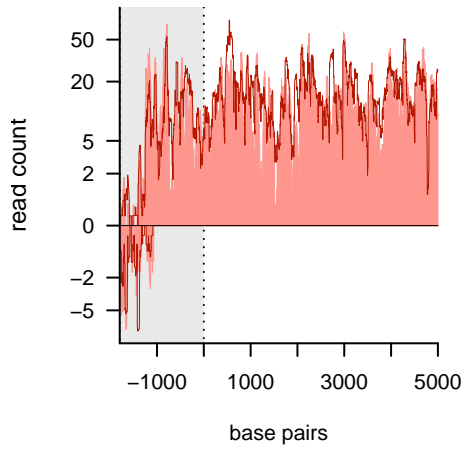

Supplement: Figure S5 — Transcription starts at 5′-telomere ends. [file mbio.02241-24-s0004.pdf]

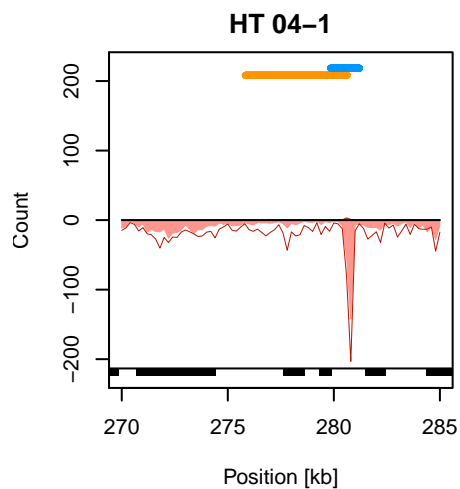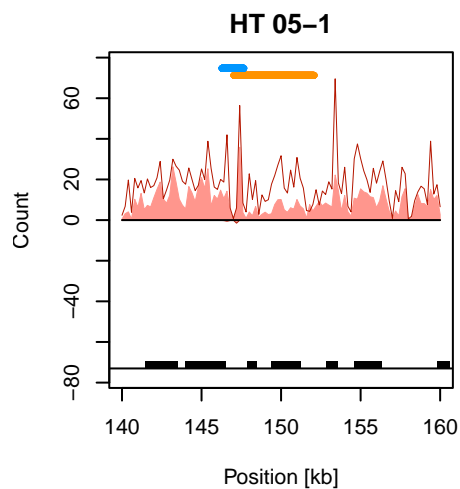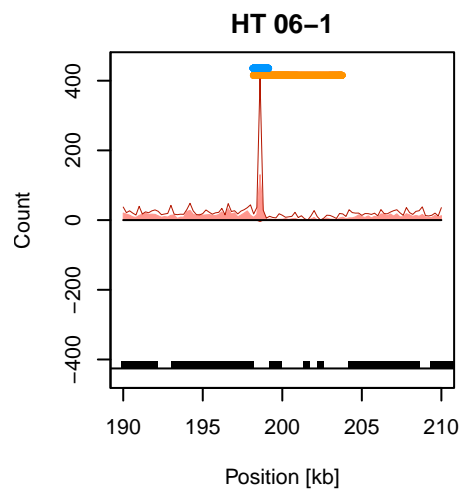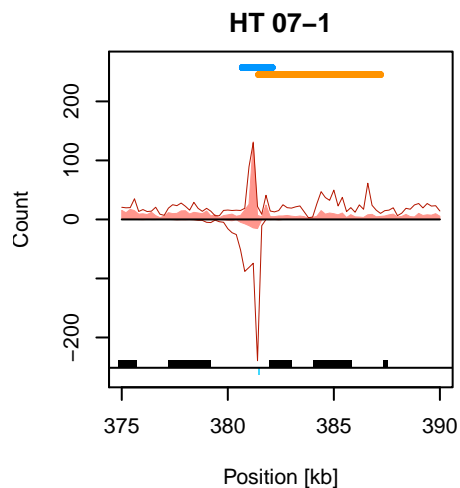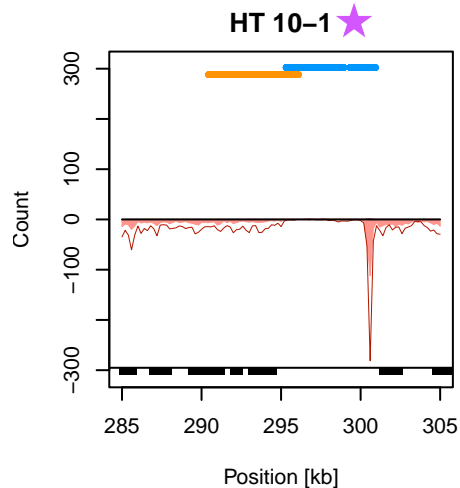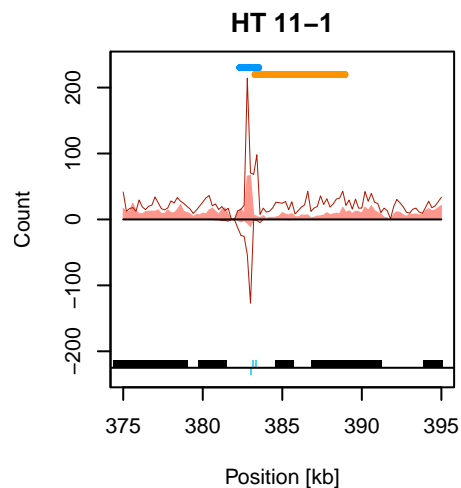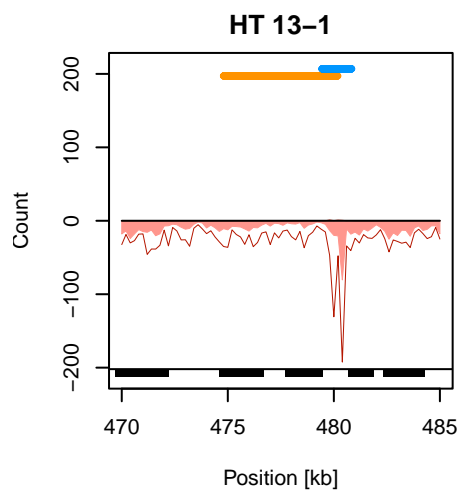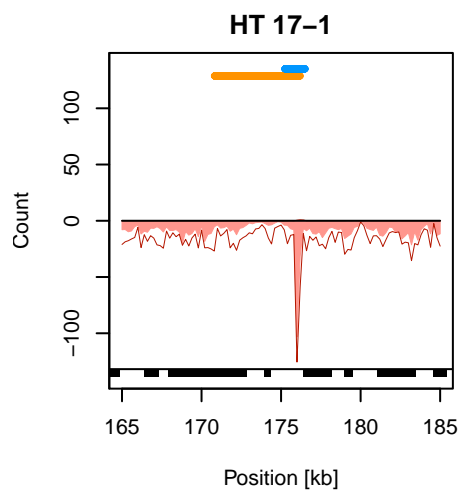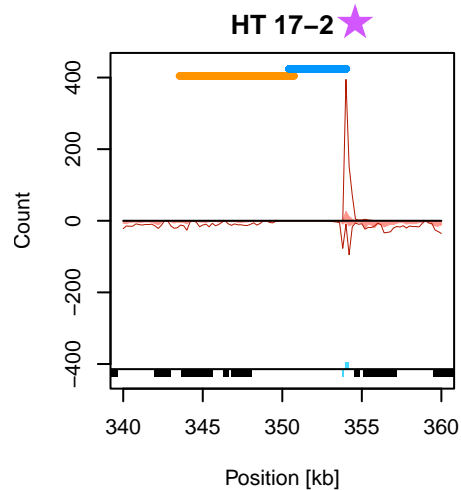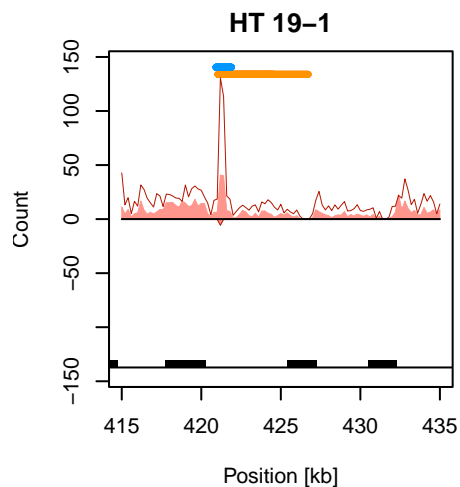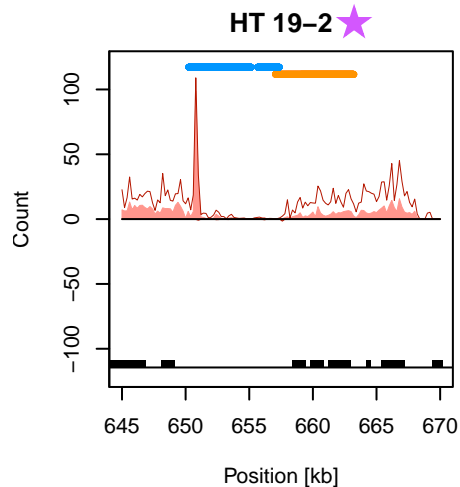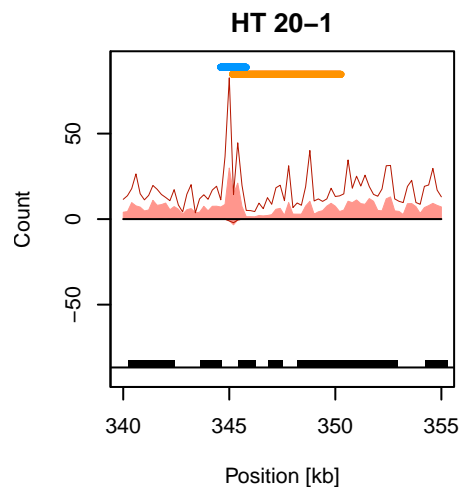

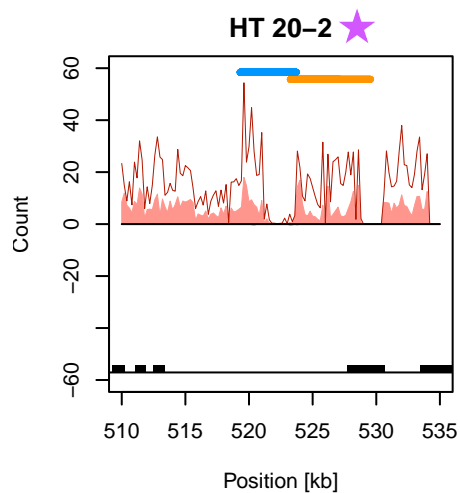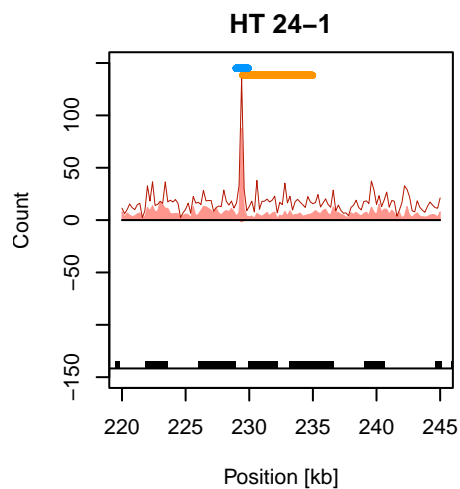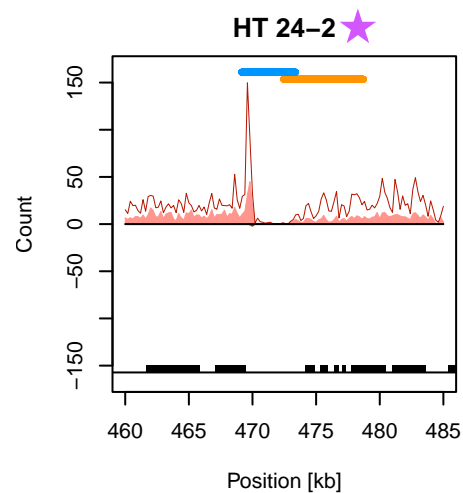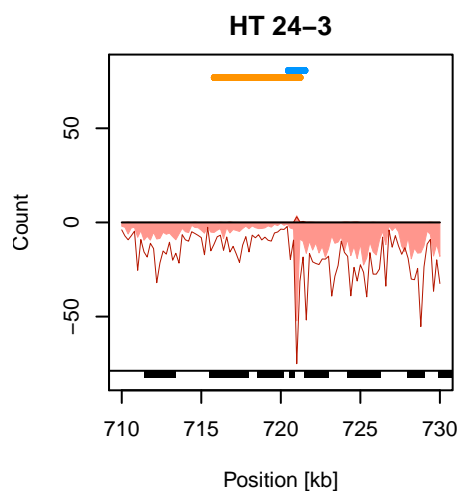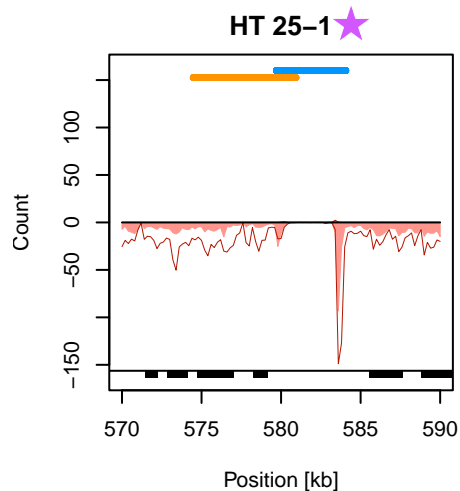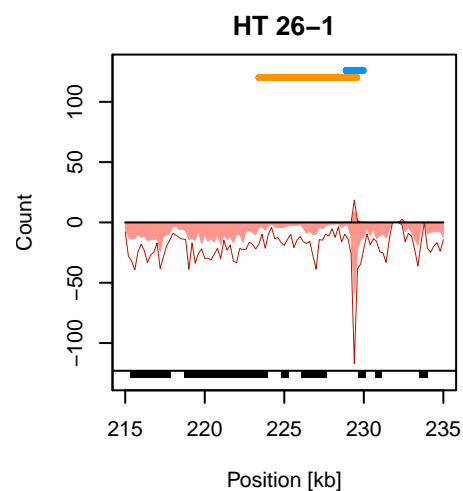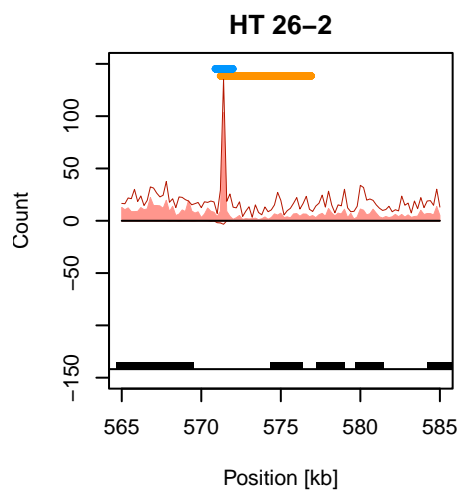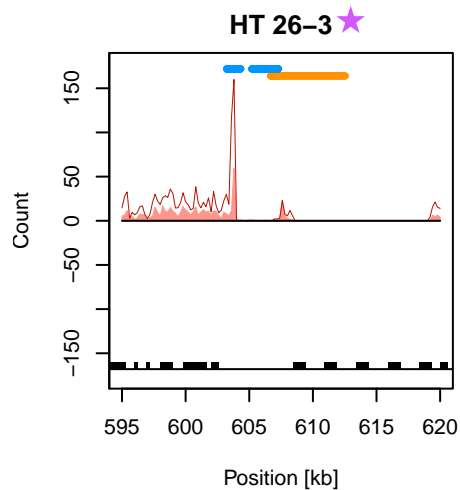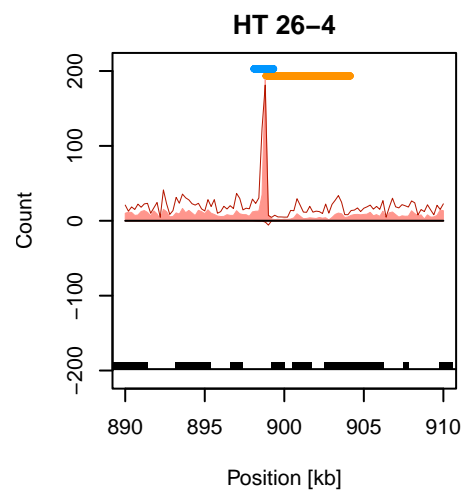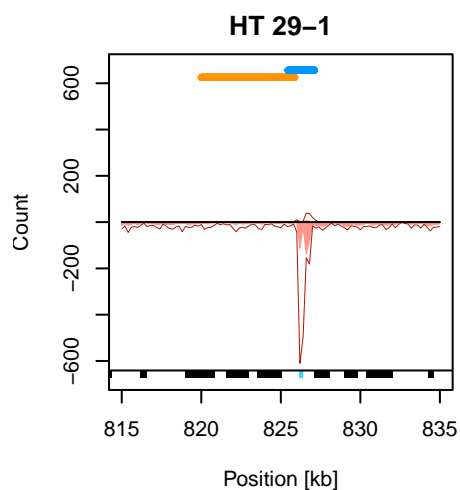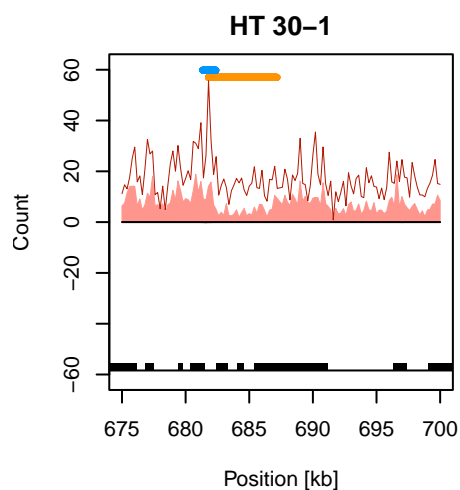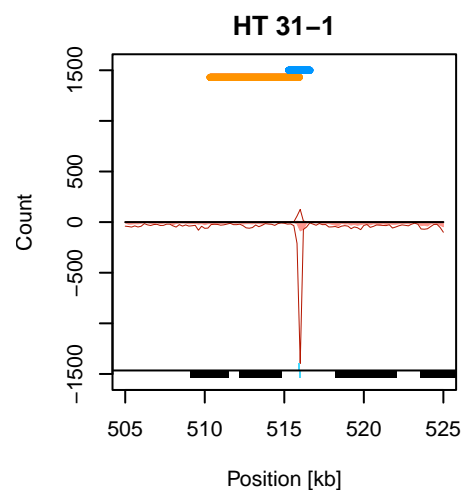

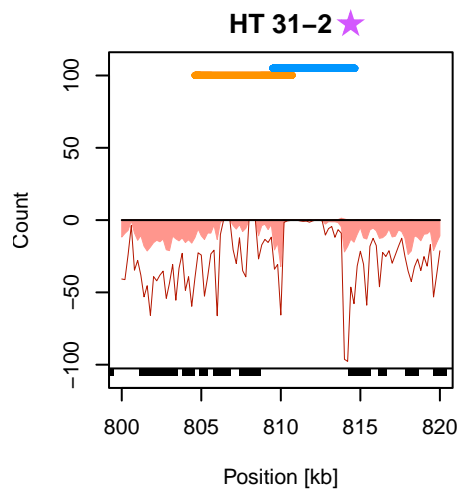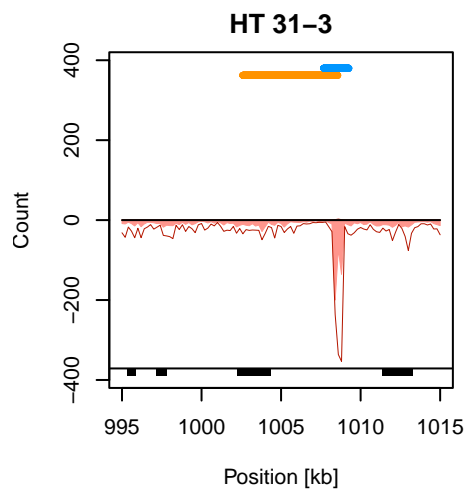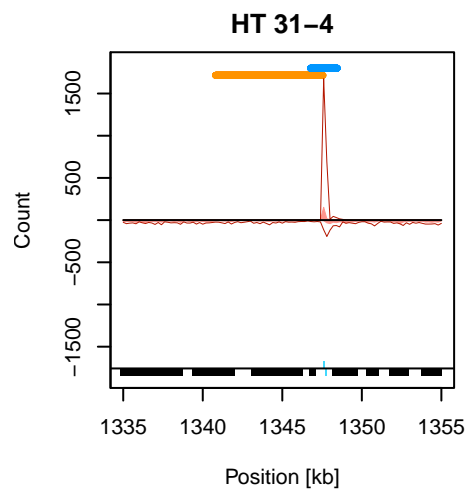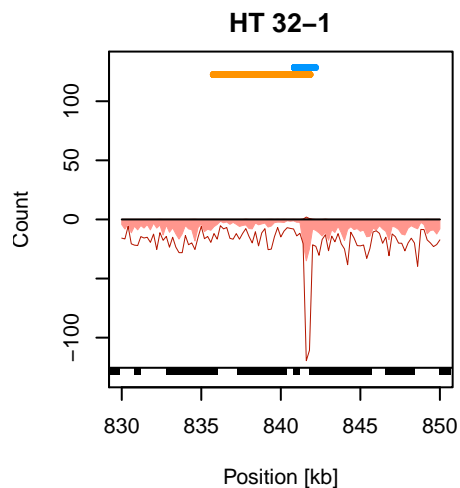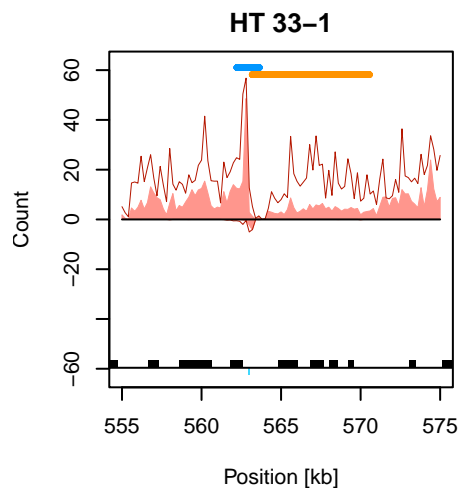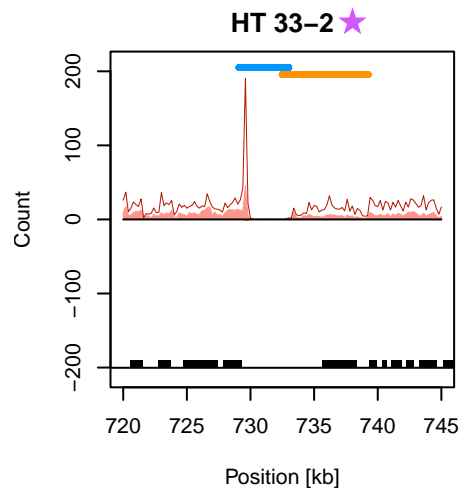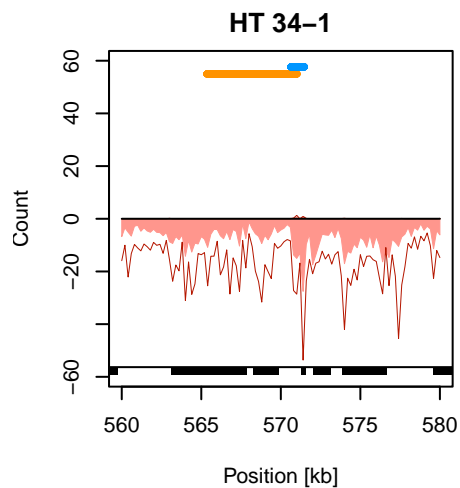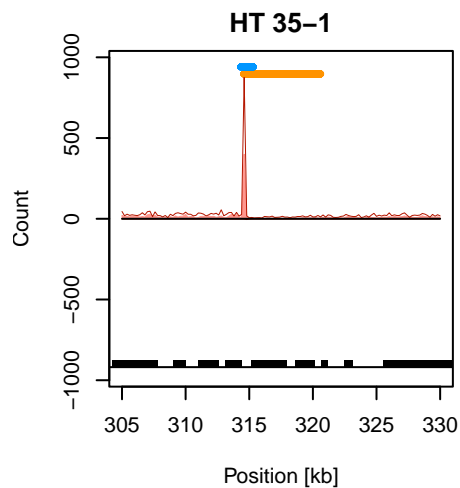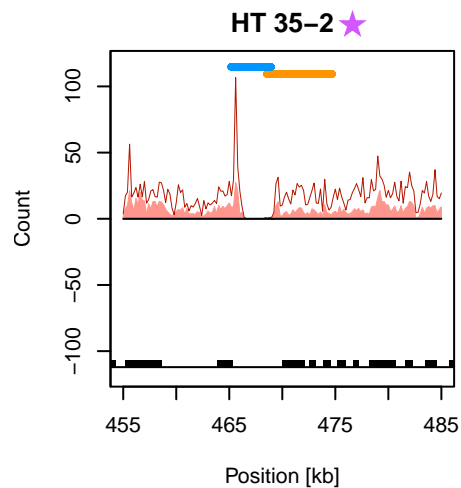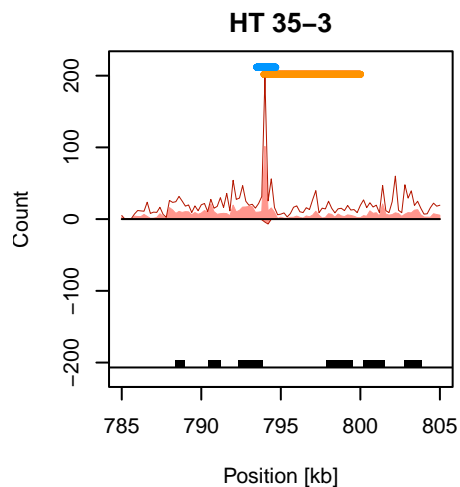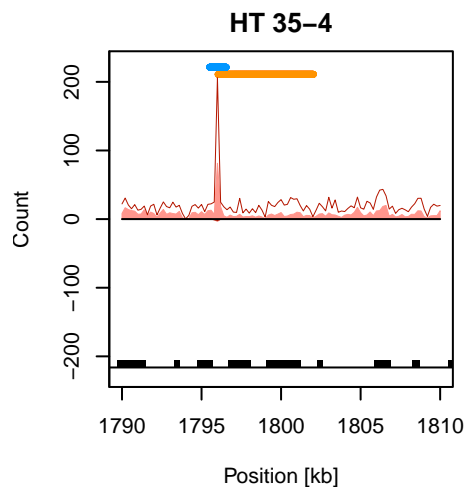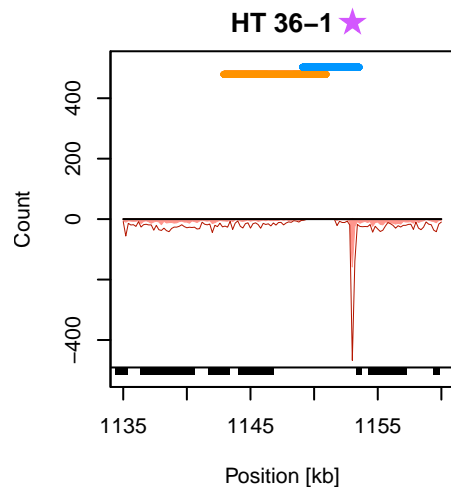

HT 36-2

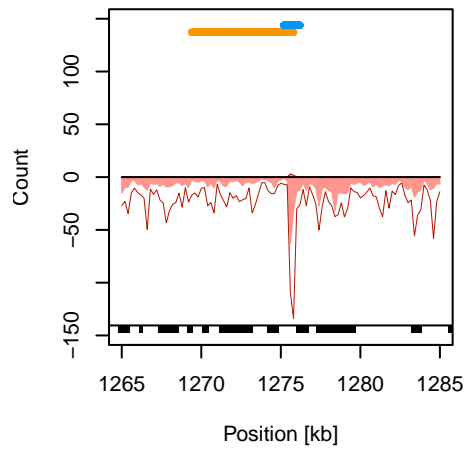

HT 36-3

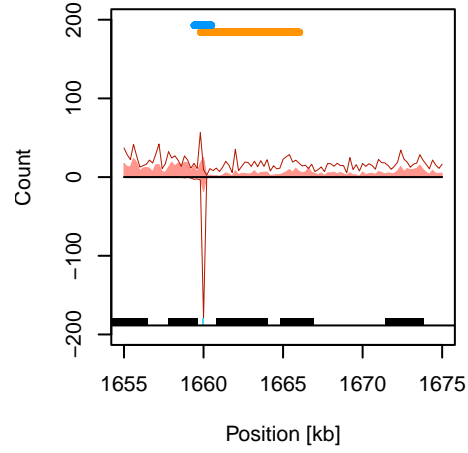

HT 36-4

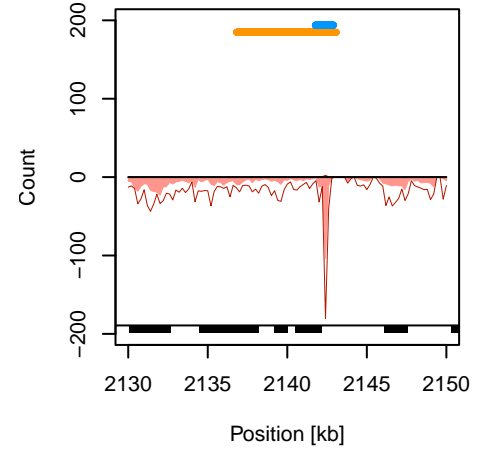

HT 36-5

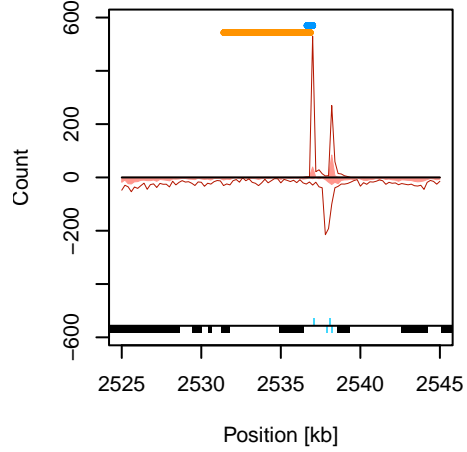

Supplement: Figure S11 — Head tail regions of all 36 chromosomes. [file mbio.02241-24-s0006.pdf]

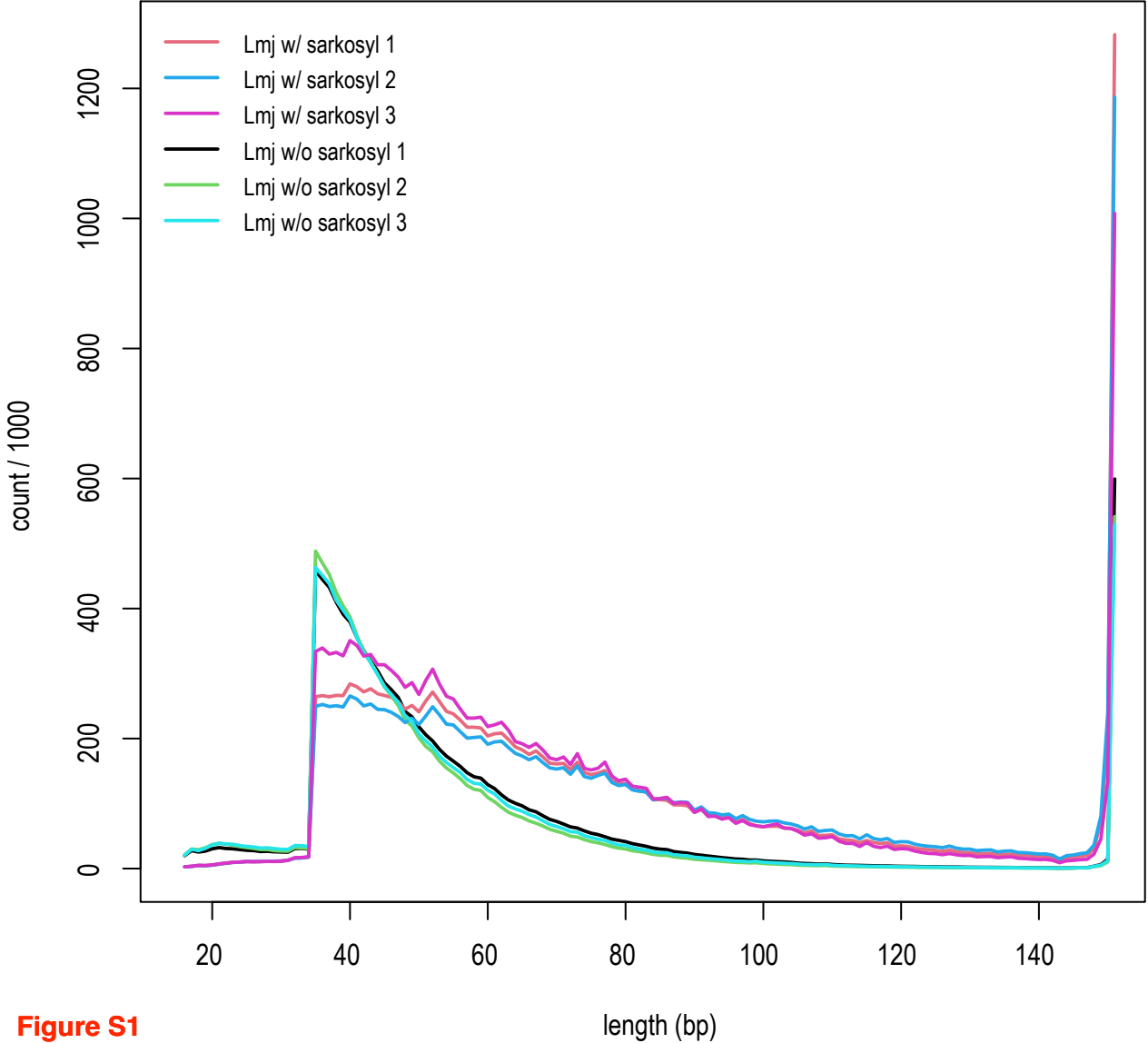

**Figure S1**

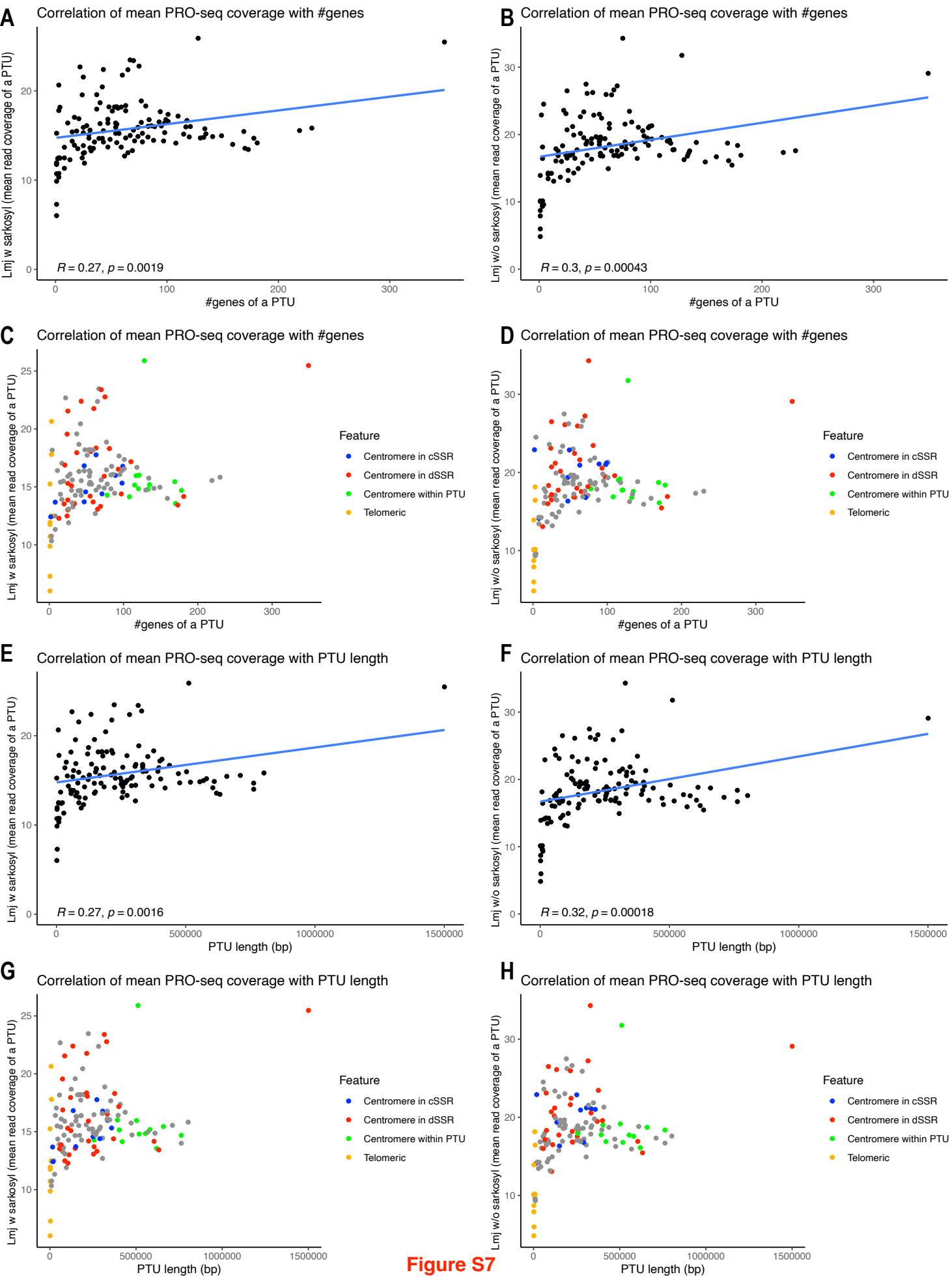

**Figure S7**

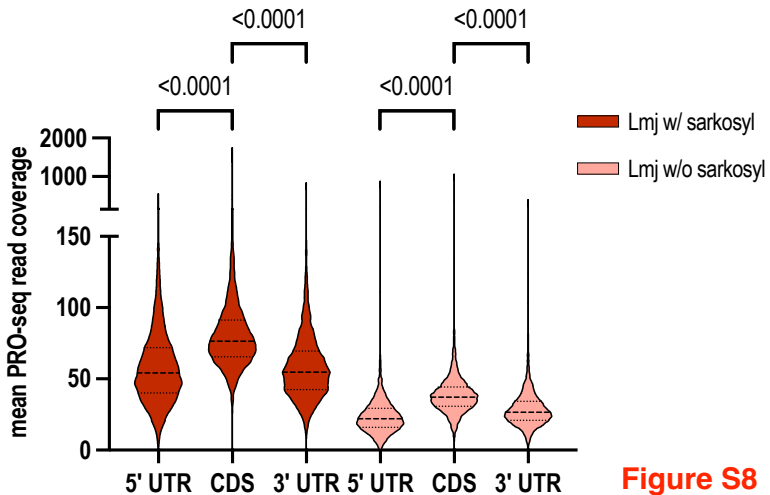

**Figure S8**

# cSSR 26 (Chr 28)

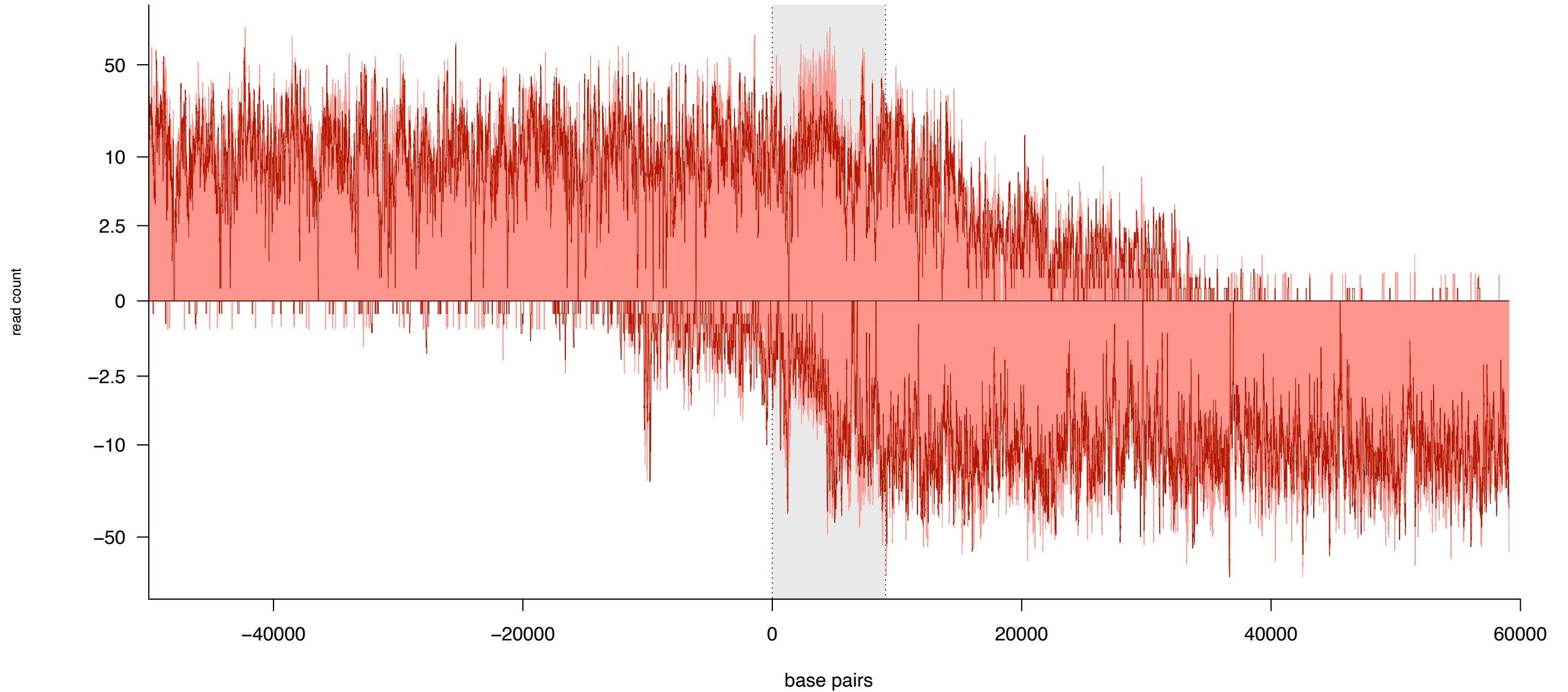

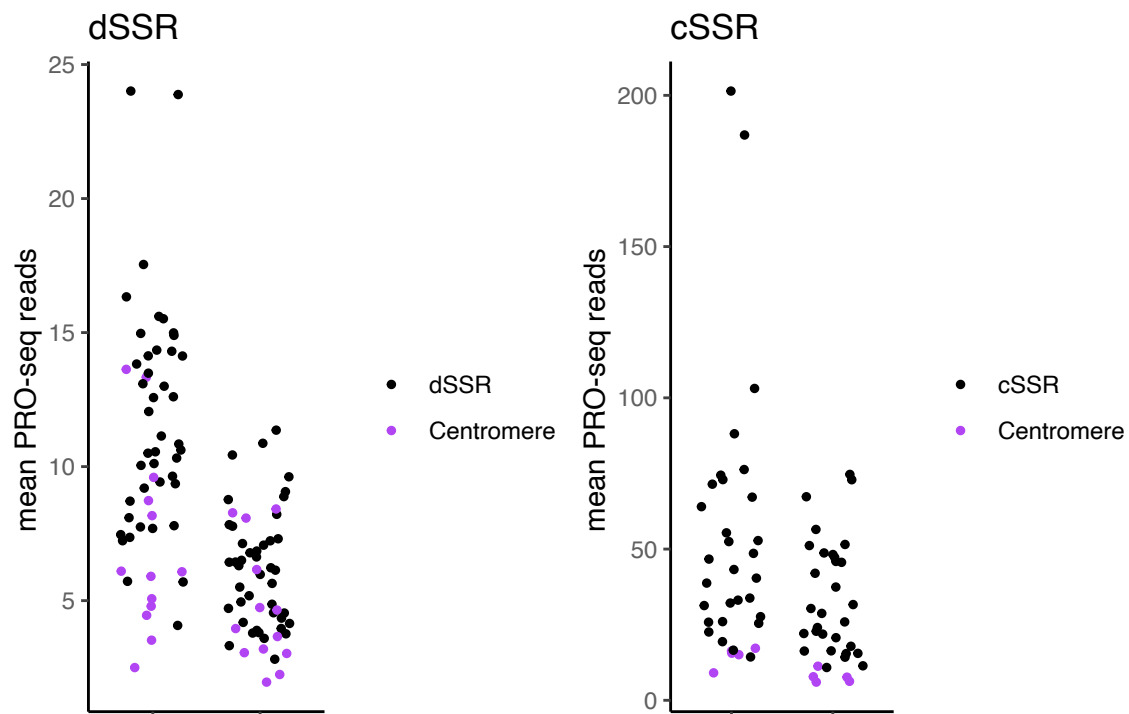

**Figure S10**

Supplement: Figures S1 and S7-S10 — Genome-wide read alignments. [file mbio.02241-24-s0007.pdf]
