# Supplementary material for: Genome-wide quantification of polycistronic transcription in Leishmania major
Source: mBio. 2024 Nov 25;16(1):e02241-24. doi: 10.1128/mbio.02241-24 (PMC11708010; doi:10.1128/mbio.02241-24)
Supplement: Figure S6 — Transcription at 3′-telomere ends. [file mbio.02241-24-s0005.pdf]

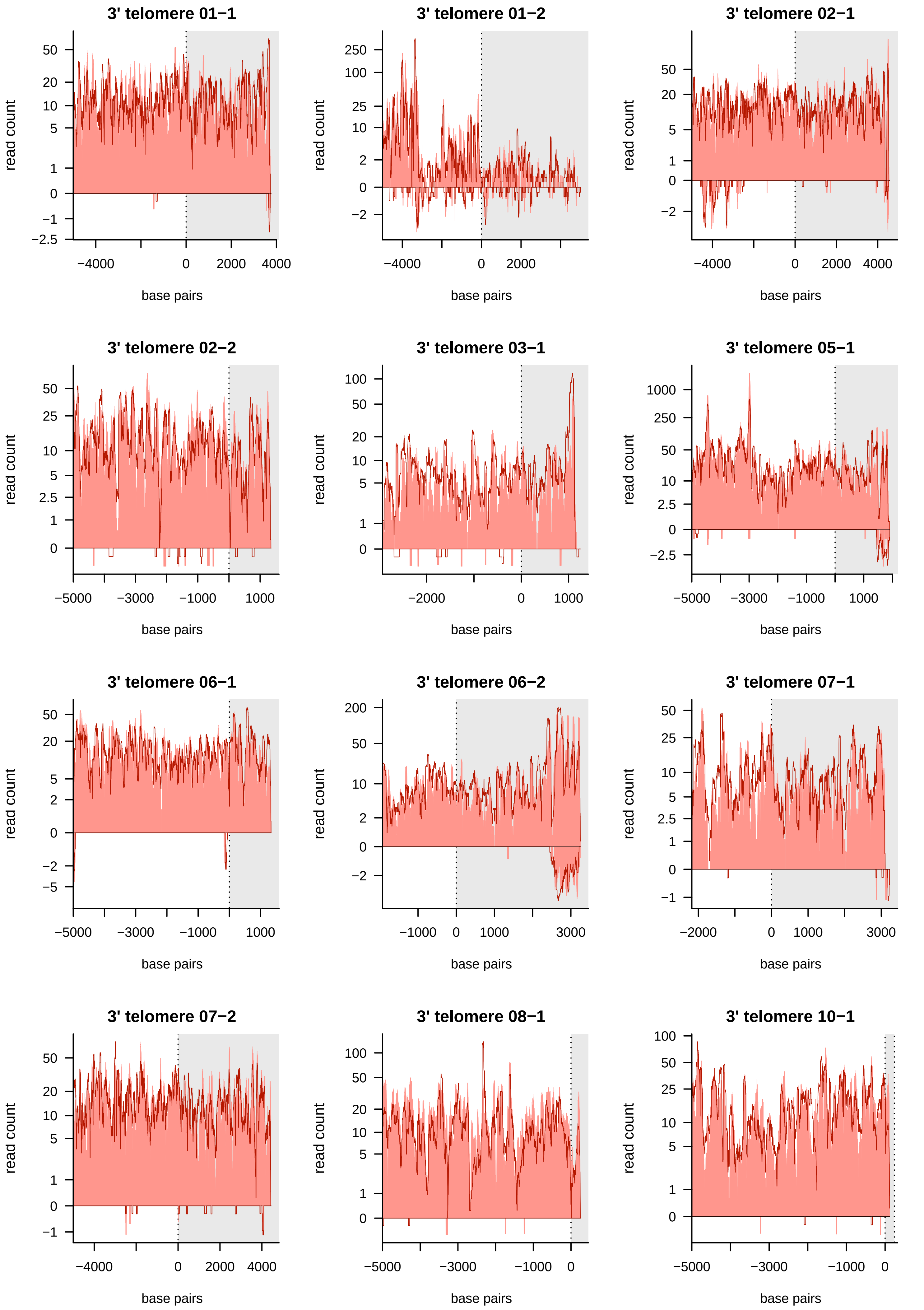

3' telomere 01−1
read count
50
20
10
5
1
0
−1
−2.5
−4000
0
2000
4000
base pairs
3' telomere 01−2
read count
250
100
25
10
2
0
−2
−4000
0
2000
base pairs
3' telomere 02−1
read count
50
20
5
1
0
−2
−4000
0
2000
4000
base pairs
3' telomere 02−2
read count
50
25
10
5
2.5
1
0
−5000
−3000
−1000
1000
base pairs
3' telomere 03−1
read count
100
50
20
10
5
1
0
−2000
0
1000
base pairs
3' telomere 05−1
read count
1000
250
50
10
2.5
0
−2.5
−5000
−3000
−1000
1000
base pairs
3' telomere 06−1
read count
50
20
5
2
0
−2
−5
−5000
−3000
−1000
1000
base pairs
3' telomere 06−2
read count
200
50
10
2
0
−2
−1000
0
1000
3000
base pairs
3' telomere 07−1
read count
50
25
10
5
2.5
1
0
−1
−2000
0
1000
3000
base pairs
3' telomere 07−2
read count
50
20
10
5
1
0
−1
−4000
0
2000
4000
base pairs
3' telomere 08−1
read count
100
50
20
10
5
1
0
−5000
−3000
−1000
0
base pairs
3' telomere 10−1
read count
100
50
25
10
5
1
0
−5000
−3000
−1000
0
base pairs

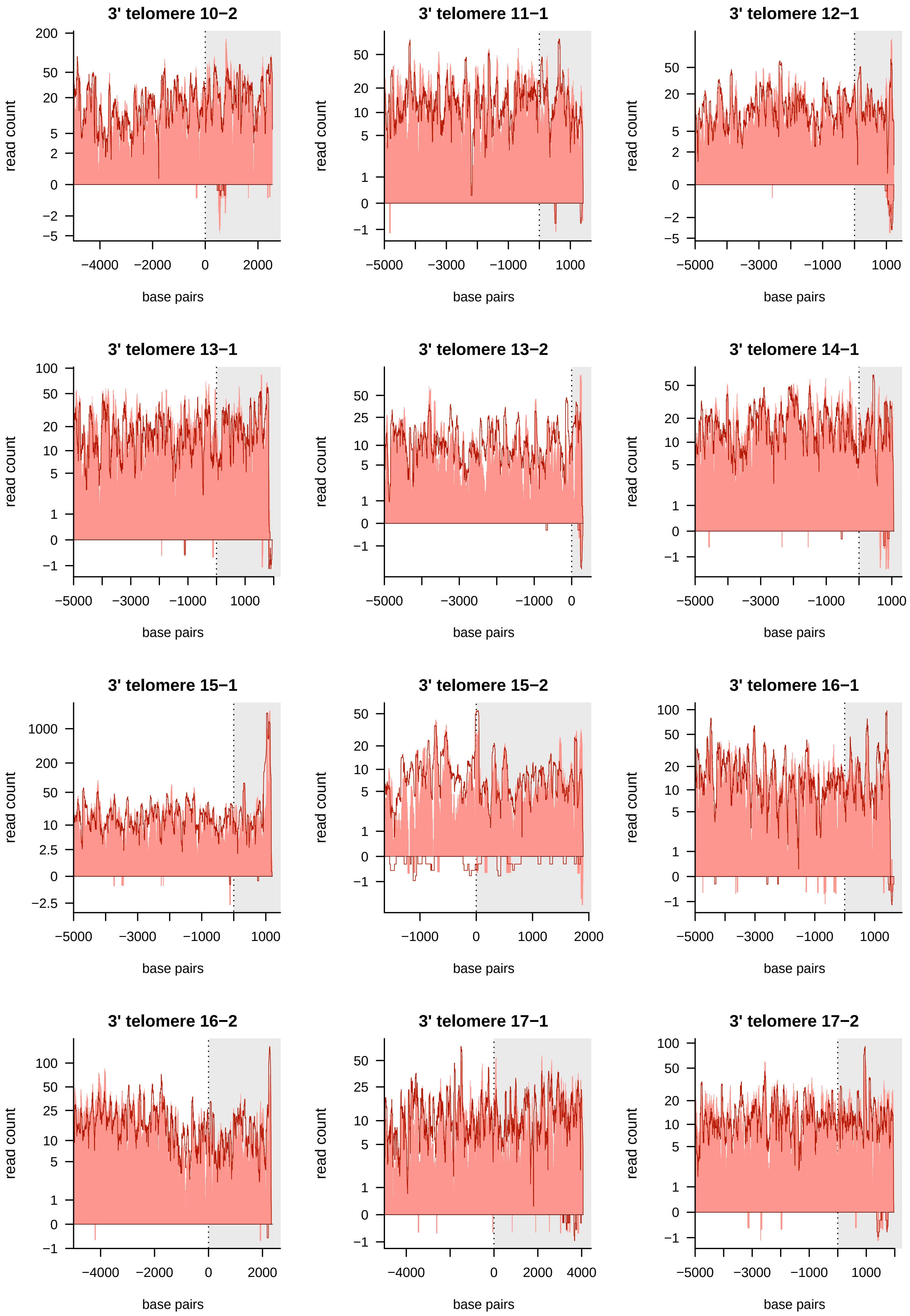
3' telomere 10−2
3' telomere 11−1
3' telomere 12−1
3' telomere 13−1
3' telomere 13−2
3' telomere 14−1
3' telomere 15−1
3' telomere 15−2
3' telomere 16−1
3' telomere 16−2
3' telomere 17−1
3' telomere 17−2
read count
base pairs

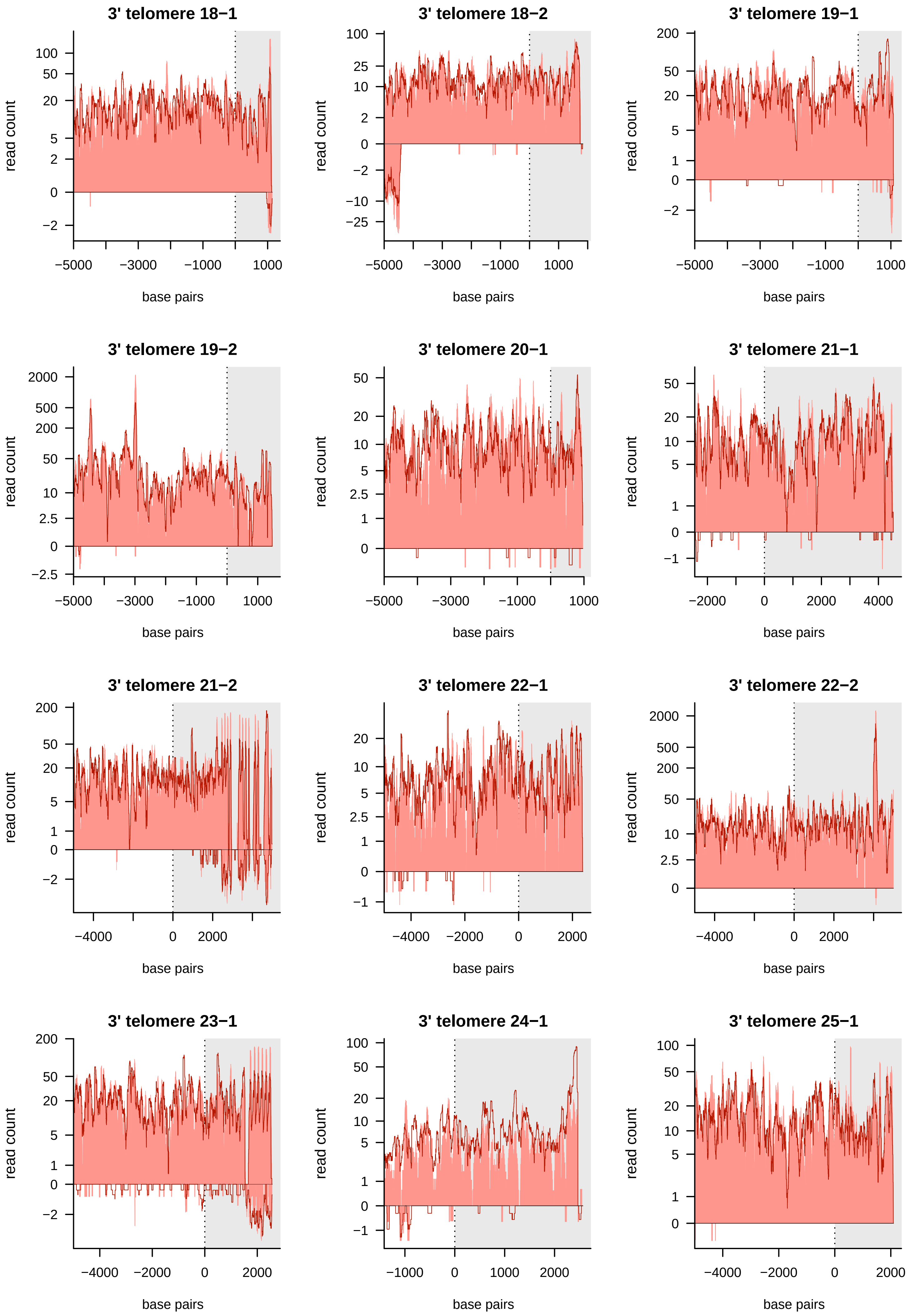

3' telomere 18−1
3' telomere 18−2
3' telomere 19−1
3' telomere 19−2
3' telomere 20−1
3' telomere 21−1
3' telomere 21−2
3' telomere 22−1
3' telomere 22−2
3' telomere 23−1
3' telomere 24−1
3' telomere 25−1
read count
base pairs

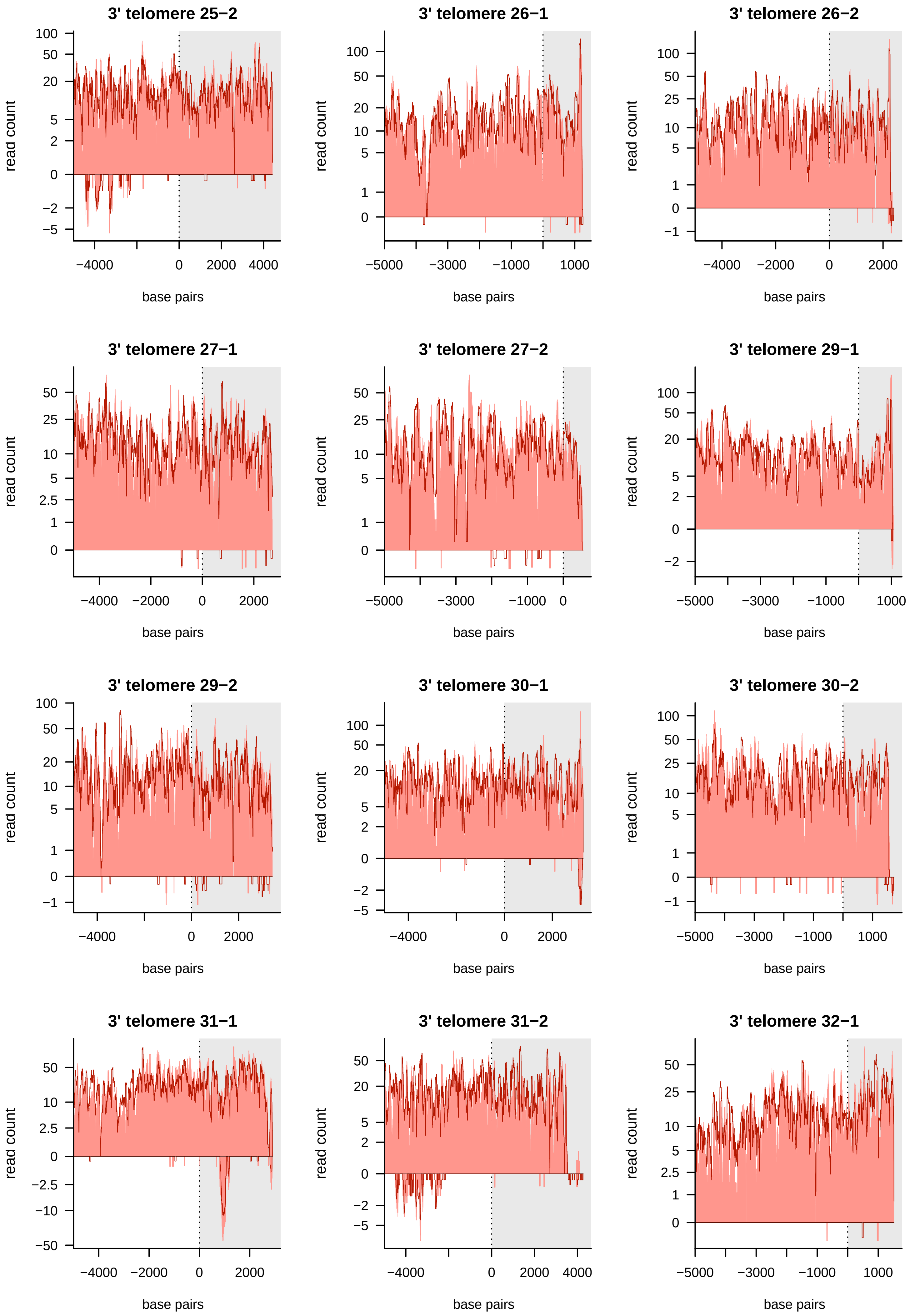

3' telomere 25−2
3' telomere 26−1
3' telomere 26−2
3' telomere 27−1
3' telomere 27−2
3' telomere 29−1
3' telomere 29−2
3' telomere 30−1
3' telomere 30−2
3' telomere 31−1
3' telomere 31−2
3' telomere 32−1
read count
base pairs

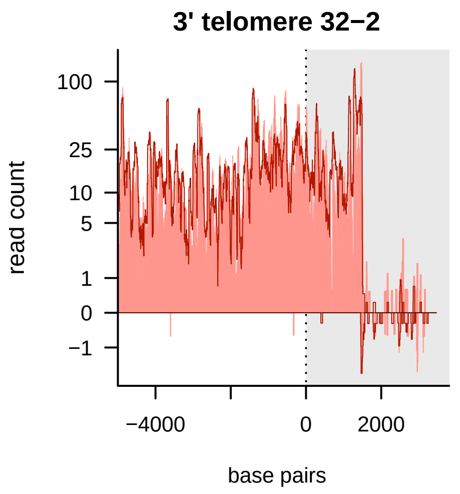
3' telomere 32−2
read count
100
25
10
5
1
0
−1
−4000
0
2000
base pairs

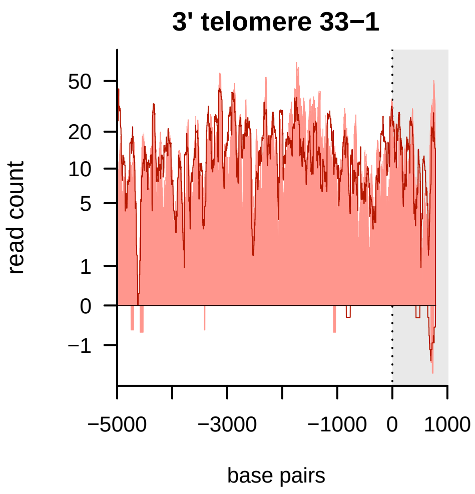
3' telomere 33−1
read count
50
20
10
5
1
0
−1
−5000
−3000
−1000
0
1000
base pairs

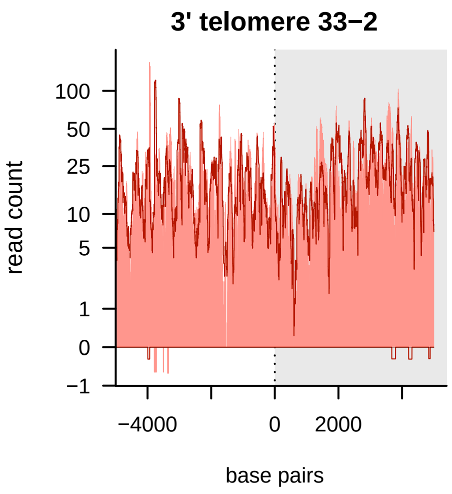
3' telomere 33−2
read count
100
50
25
10
5
1
0
−1
−4000
0
2000
base pairs

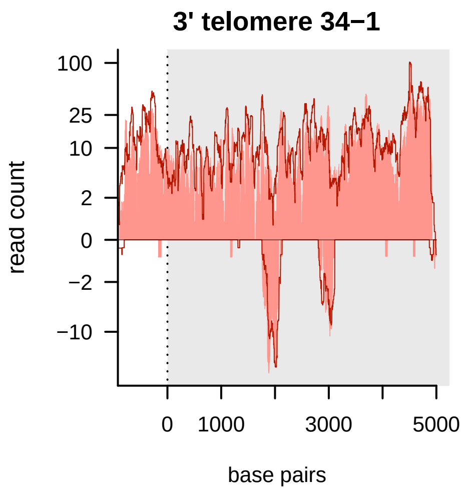
3' telomere 34−1
read count
100
25
10
2
0
−2
−10
0
1000
3000
5000
base pairs

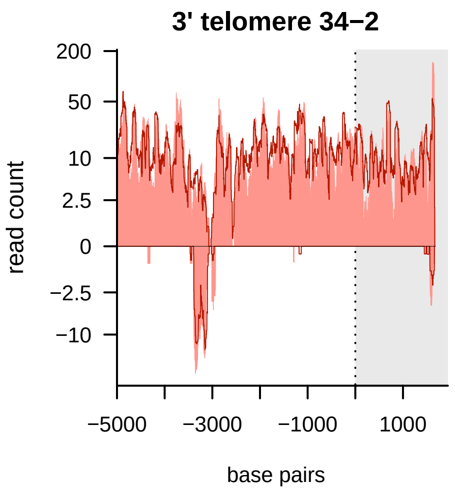
3' telomere 34−2
read count
200
50
10
2.5
0
−2.5
−10
−5000
−3000
−1000
1000
base pairs

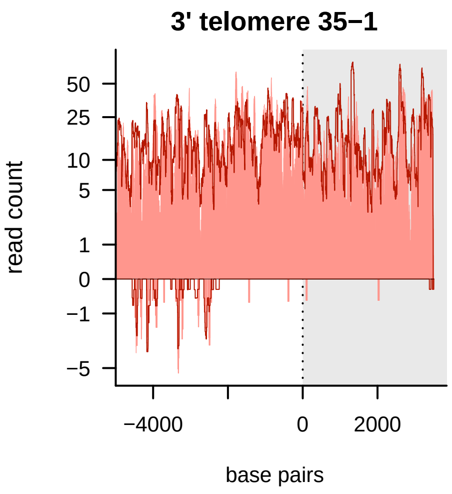
3' telomere 35−1
read count
50
25
10
5
1
0
−1
−5
−4000
0
2000
base pairs

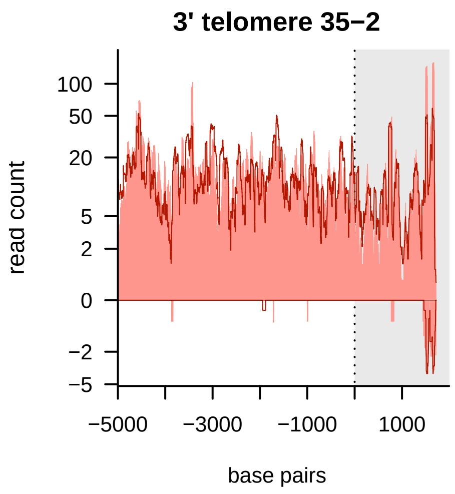
3' telomere 35−2
read count
100
50
20
5
2
0
−2
−5
−5000
−3000
−1000
1000
base pairs

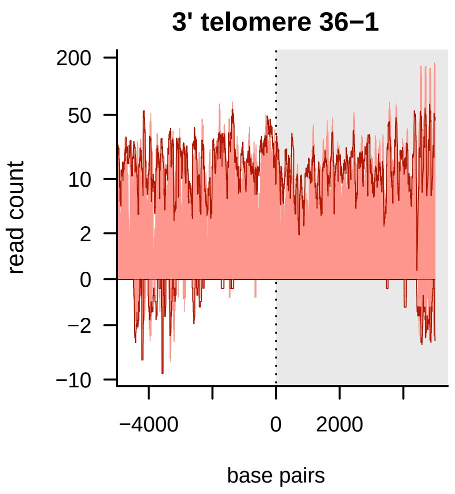
3' telomere 36−1
read count
200
50
10
2
0
−2
−10
−4000
0
2000
base pairs
