# Supplementary material for: Genome-wide quantification of polycistronic transcription in Leishmania major
Source: mBio. 2024 Nov 25;16(1):e02241-24. doi: 10.1128/mbio.02241-24 (PMC11708010; doi:10.1128/mbio.02241-24)
Supplement: Legends — Supplemental figure legends. [file mbio.02241-24-s0008.docx]

**Figure S1:** Read length analysis for PRO-seq reads from 6 reactions.

**Figure S2:** Linear plot of PRO-seq reads against all 36 chromosomes of the *L. major* Friedlin 2021 genome. From top to bottom, the plots show the centromere (purple) (Garcia-Silva et al., 2017) BaseJ (blue) (van Luenen et al., 2012) and H3ac (orange) (Thomas et al., 2009), PRO-seq read densities from nuclei without sarkosyl (upper and lower strands), PRO-seq read densities from nuclei without sarkosyl (upper and lower strands), and *L. major* genes with UTRs at half-height and CDSs at full height. Genes in grey: non coding RNAs, genes in blue: RNA Pol III transcribed genes.

**Figure S3: Transcription starts at dSSRs.** Distribution of normalised PRO-seq reads from *L. major* nuclei incubated with sarkosyl (dark red outline) or without sarkosyl (light red shading) around dSSR regions. For chromosomal locations of dSSRs (grey shade), see Table S2. Blue shade: RNA Pol III transcribed genes.

**Figure S4: Transcription stops at cSSRs.** Distribution of normalised PRO-seq reads from *L. major* nuclei incubated with sarkosyl (dark red outline) or without sarkosyl (light red shading) around cSSR regions. For chromosomal locations of cSSRs (grey shade), see Table S2. Blue shade: RNA Pol III transcribed genes.

**Figure S5: Transcription starts at 5’-telomer ends.** Distribution of normalised PRO-seq reads from *L. major* nuclei incubated with sarkosyl (dark red outline) or without sarkosyl (light red shading) at 5'-telomeric regions. For chromosomal locations of 5' telomers (grey shade), see Table S2.

**Figure S6: Transcription at 3'-telomer ends**. Distribution of normalised PRO-seq reads from *L. major* nuclei incubated with sarkosyl (dark red outline) or without sarkosyl (light red shading) around 3'-telomeric regions. For chromosomal locations of 3' telomers (grey shade), see Table S2.

**Figure S7: Mean PRO-Seq read coverage of PTUs correlated with length of a PTU or number of genes within a PTU. A+B:** Plotted is the number of genes within one PTU against the normalised mean PRO-seq read coverage of Lmj w sarkosyl (A) and Lmj w/o sarkosyl (B). **C+D:** The PTUs are coloured by their feature (telomeric, centromere within PTU, centromere in adjacent dSSR or cSSR). **E+F:** Plotted is the length of a PTU against the mean PRO-seq read coverage of Lmj w sarkosyl and Lmj w/o sarkosyl. The features are coloured in **G+H**. **n=3.**

**Figure S8: Mean PRO-seq coverage at CDS and UTRs.** Violin plots are based on all protein-coding genes. Normalised mean PRO-seq coverage from Lmj w sarkosyl and Lmj w/o sarkosyl from three biological replicates are shown. Significance was tested with a Friedmann test with post-Dunns comparison.

**Figure S9: Transcription termination within convergent SSRs 26 on chromosome 28.** cSSRs 26 is shown in grey and the dashed lines represent the stop of the PTU with the last 50kb of the PTU shown. Normalised PRO-seq reads of *L. major* with sarkosyl are plotted logarithmically as dark red line and reads of *L. major* without sarkosyl as light red background.

**Figure S10: Mean PRO-seq coverage at dSSRs and cSSRs with centromeric locations.** Scatter plot shows the normalised mean PRO-seq read coverage from Lmj w sarkosyl and Lmj w/o sarkosyl, where every dot represents one SSR from three biological replicates. SSRs, where a centromere is located, are coloured in purple. We excluded SSRs with RNA Pol I & IlI transcribed genes.

**Figure S11: Head tail regions of all 36 chromosomes are shown.** Distribution of normalised PRO-seq reads (mean of three replicates) of samples with sarkosyl are plotted as a dark red line and samples without sarkosyl are plotted as a light red background. BaseJ peaks are shown in blue and H3ac peaks in orange. Genes are plotted as a black box at the bottom of the plots. The purple star indicates a centromere is present. The small turqioise bars are RNA Pol III transcribed genes.
